# Global, regional, and national burden of acute myeloid leukemia, 1990–2021: a systematic analysis for the global burden of disease study 2021

**DOI:** 10.1186/s40364-024-00649-y

**Published:** 2024-09-11

**Authors:** Yeming Zhou, Guiqin Huang, Xiaoya Cai, Ying Liu, Bingxin Qian, Dengju Li

**Affiliations:** Department of Hematology, Tongji Hospital, Tongji Medical College, Huazhong University of Science and Technology, Wuhan, China

**Keywords:** Acute myeloid leukemia, Global burden disease, Cancer epidemiology, Cancer statistics, Population aging, Social-demographic index, Age-standardized incidence rate, Age-standardized death rate, Age-standardized DALY rate, Estimated annual percentage change

## Abstract

**Background:**

Acute myeloid leukemia (AML), as the most common subtype of leukemia in adults, is characterised by rapid progression and poor prognosis. In the context of the rapid development of medical technology and the complexity of social factors, a detailed report describing the latest epidemiological patterns of AML is important for decision makers to allocate healthcare resources effectively.

**Methods:**

Our research utilized the latest data sourced from the Global Burden of Disease (GBD) 2021. To delineate the burden of AML, we comprehensively described the incidence, deaths, disability-adjusted life years (DALYs), and the associated age-standardized rates per 100,000 persons (ASR) spanning from 1990 to 2021 stratifies according to age, sex, socio-demographic index (SDI), and nationality. Additionally, we extracted and analyzed data about the risk factors that contribute to AML-related deaths and DALYs.

**Results:**

According to our study, the incidence of AML has continued to rise globally from 79,372 in 1990 to 144,645 in 2021 and AML affected the male and the elderly populations disproportionately. Furthermore, there was a significant positive correlation between the burden of AML and the SDI value. Developed nations generally exhibited higher age-standardized incidence rate, age-standardized death rate, and age-standardized disability-adjusted life year rate than the developing nations. We also analyzed the prevalence of smoking, high body mass index, and occupational benzene and formaldehyde exposure in the AML population in different SDI regions. Moreover, smoking and high body mass index were more prevalent in developed countries, whereas occupational exposure to these chemicals was the predominant risk factor in developing countries.

**Conclusion:**

The global burden of AML has increased over the past 32 years, with rising morbidity and mortality. The incidence of AML is differentially distributed across different SDI countries or regions. AML incidence is higher in the elderly and in men. The proportions of smoking, high body mass index, and occupational exposure to benzene and formaldehyde varied by region. The findings highlight the need for region-specific prevention and call for future research on preventive strategies and new treatments to lower AML incidence and improve patient outcomes.

**Supplementary Information:**

The online version contains supplementary material available at 10.1186/s40364-024-00649-y.

## Introduction

Acute myeloid leukemia (AML) is a genetically heterogeneous clonal disease that originates in the bone marrow [1], marked by abnormal proliferation and arrested differentiation of primitive bone marrow cells, impairing normal hematopoietic function and leading to life-threatening cytopenia and transfusion dependency [2]. AML can affect any age group but is most prevalent among adults, with its incidence increasing with age [3]. Given the trend of population aging, AML incidence is expected to rise annually [4]. With the rapid advances in medical diagnostic, treatment technologies, accelerated population ageing, and the complex influence of social factors such as lifestyle [5, 6] and occupational exposures [7, 8], the global disease burden of AML is undergoing a rapid and profound change. Thus, the global disease burden of AML merits an updated assessment to understand its specific impact on public health better.

AML is notable for its genetic heterogeneity, manifested by a wide range of molecular and cytogenetic abnormalities, including changes in chromosome number and structure, mutations, and the formation of fusion genes [9–11]. The diagnosis of AML requires comprehensive multilevel testing including cytomorphological, cytogenetic and molecular genetic assessment to determine its genetic heterogeneity for subsequent precise medication. For example, AML carrying a PML-RARA fusion gene resulting from a t(15;17) translocation is diagnosed as acute neutrophilic leukemia, and treatment with all-trans retinoic acid and arsenite may be very effective [12]. Other patients may have mutations in genes such as FLT3-ITD, TP53, etc., which usually portend a poorer prognosis and require the addition of appropriate targeted agents or a more aggressive treatment regimen during subsequent therapy [13–15]. Nowadays, the treatment of AML usually consists of induction chemotherapy and consolidation therapy after remission. Depending on prognostic stratification and individual patient factors, hematopoietic stem cell transplantation may be considered to minimize the risk of disease recurrence. The advent of novel targeted therapeutic agents and strategies has markedly improved the remission rates among AML patients [16, 17]. The impact of consolidation therapy and minimal residual disease (MRD) monitoring on the recurrence rate is multifaceted [18]. Despite the emergence of new treatments, conventional intensive chemotherapy remains the predominant modality for AML treatment [19], accompanied by significant complications such as severe infections, anaemia, and thrombocytopenia due to bone marrow suppression. These adverse events are closely associated with patients’ baseline characteristics and necessitate substantial economic resources for supportive care [20–22]. The variability in the disease burden of AML across different countries can be attributed to factors such as the widespread implementation of early screening, the sophistication of diagnostic testing, the availability of novel targeted drugs, chimeric antigen receptor T-cell (CAR-T) therapy, and the disparities in medical resource allocation.

The Global burden of disease (GBD) study is a seminal resource for comprehending the epidemiological landscape of various diseases, encompassing their prevalence, incidence, deaths, and disability-adjusted life years (DALYs). Compared with previous studies on AML using GBD data [23, 24], we gained information from AML-specific data from the latest 2021 GBD study. In addition, the present study provides a more comprehensive breakdown of AML incidence, deaths, DALYs, and risk factors by age, sex, geographic region, and social development index (SDI), with a focus on SDI distributions of disease burden and temporal trends. The present analysis is poised to assist clinicians, epidemiologists, and health policymakers in enhancing the distribution of medical resources and in formulating more robust public health strategies.

## Methods

### Data source

GBD 2021 study meticulously gathers data on the incidence and mortality rates of 288 causes of death from an array of sources. Key data sources encompass vital registration and verbal autopsy, which furnish comprehensive details on all 288 causes. To enhance this, the study also incorporates surveys, censuses, surveillance systems, and cancer registries, providing more nuanced information. Furthermore, data from police records, open-source databases, and minimally invasive tissue sampling are used to capture insights on specific diseases and injuries. For data processing and estimation, GBD 2021 utilizes advanced techniques like the Cause of Death Ensemble Model (CODEm), guaranteeing the precision and robustness of the results. In cases where data is limited or the epidemiology is unusual, the study employs alternative models and approaches. When cancer registry coverage or reliable mortality data is absent, GBD 2021 turns to methods such as predictive modeling, data sharing, and expert consultation to estimate the missing data [25].

Data specific to AML consisting of incidence, deaths, DALYs, and corresponding age-standardized rates, were downloaded from the Global Health Data Exchange (GHDx) website (https://vizhub.healthdata.org/gbd-results/) [26]. Furthermore, the GBD study quantifies the levels and trends of 88 contributory risk factors linked to the disease burden, employing a framework for comparative risk assessment [27].

The incidence and mortality of AML were identified using the International Classification of Diseases, Ninth Revision (ICD-9) and Tenth Revision (ICD-10). Specifically, AML incidence was identified through the ICD-9 codes C92.0-C92.02, C92.3-C92.62, C93.0-C93.02, C94.0-C94.02, C94.2-C94.22, C94.4-C94.5 or ICD-10 codes 205.0205.02, 205.2–205.32, 206.0–206.02, 207.0–207.02, 207.2–207.82. Mortality was identified through the ICD-9 codes C92.0, C92.3-C92.6, C93.0, C94.0, C94.2, C94.4-C94.5 or ICD-10 codes 205.0, 205.2–205.3, 206.0, 207.0, 207.2–207.8.

Background information, such as the SDI, was also gathered for subsequent correlational analyses. The SDI values, ranging from 0 to 1, reflect the level of social development in a country. Summary exposure value (SEV) is the RR-weighted prevalence of exposure and a univariate measure of risk-weighted exposure. A value of SEV = 0 indicates the population is not at excess risk and SEV = 1 indicates the population is at its highest risk. In GBD 2021, final values are multiplied by 100 for a scale of 0–100, with 100 indicating the population is at the highest prevalence and 0 indicating the population is at the lowest prevalence. Furthermore, the GBD 2021 study stratified the global countries into five quintiles based on the SDI (higher, high-middle, middle, low-middle, and lower) and 21 geographical regions.

### Statistical analysis

Prior research has extensively detailed the protocols and methods for GBD research [28]. In this study, we employed annual incidence cases, death cases, DALYs, and their respective age-standardized rates per 100,000 persons (ASR) to illustrate the burden of AML.

The ASR (per 100,000 population) in accordance with the direct method is calculated by summing up the products of the age-specific rates (a.i., where i denotes the ith age class) and the number of persons (or weight) (wi) in the same age subgroup i of the chosen reference standard population, then dividing the sum of standard population weights. By utilizing age-standardized incidence rate (ASIR), age-standardized death rate (ASDR), and age-standardized DALY rate, it is possible to facilitate comparisons across populations of varying age distributions and sizes and to enhance the accuracy of comparisons between populations. DALYs function as a comprehensive metric, encapsulating the reduction in healthy life expectancy due to diseases, encompassing premature mortality and disability.

As in previous GBD rounds, cause-specific mortality rates for most causes were estimated using the Cause of Death Ensemble Model, a modeling tool developed for the GBD that evaluates the out-of-sample predictive validity of different statistical models and covariate permutations and combines these results to produce cause-specific mortality estimates, with alternative strategies to model causes of insufficient data, major changes in reporting during the study period, or epidemiological anomalies.

To analyze changes in ASR over time, we utilized Estimated Annual Percentage Change (EAPC) values. The EAPC was determined using the formula y = α + βx + ε, where y represents the natural logarithm of the ASR and x corresponds to the calendar year. Subsequently, the EAPC was calculated as 100 × (exp(β) − 1). A positive EAPC and its corresponding 95% confidence intervals (CIs) signifies an increasing trend in ASR, whereas a negative EAPC and its corresponding 95% CIs indicate a decreasing trend in ASR.

Moreover, this study employed the Pearson correlation coefficient (ρ) to examine the association between ASIR, ASDR and SDI, to elucidate the impact of socioeconomic determinants on the AML burden. In the meticulous analysis of the GBD 2021 study, the computation of Pearson correlation coefficients is carefully tailored to navigate the extensive disparities in population magnitude and case frequencies across various countries and global regions. This sophisticated process incorporates age-standardization methods and the conversion of raw data into age-standardized rates, utilizing population-weighted averages to ensure each demographic’s equitable contribution. The Pearson correlation coefficient is derived through the product-moment correlation formula, with due consideration to the statistical power and precision of the estimates, while also accounting for ecological inference to prevent erroneous causal interpretations at the individual level. Furthermore, the determination of confidence intervals for these coefficients, along with comprehensive sensitivity analyses, evaluates the stability of the results against potential data biases and quality inconsistencies, ultimately yielding robust and reliable indicators of association within the nuanced domain of international health data.

All statistical procedures and data manipulation were conducted by stringent academic protocols, utilizing R software version 3.6.3. This approach was instrumental in ensuring the academic rigor and dependability of the research findings.

## Results

### Incidence burden of AML

**Fig. 1:**
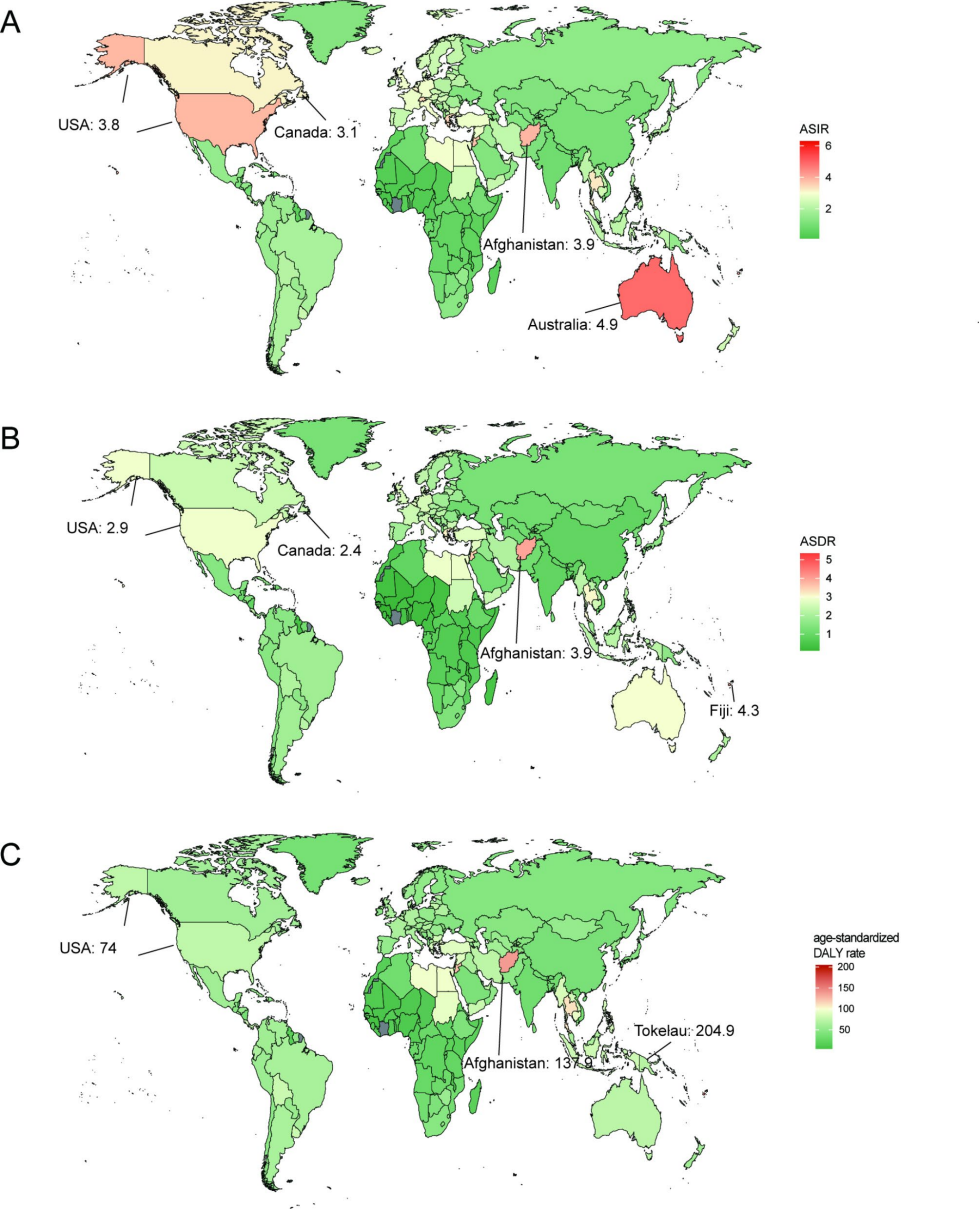
The age-standardized rates of AML in 204 countries or territories in 2021: (**A**) The ASIR of 204 countries or territories in 2021; (**B**) the ASDR of 204 countries or territories in 2021; (**C**) the age-standardized DALY rate of 204 countries or territories in 2021. AML: acute myeloid leukemia; ASIR: age-standardized incidence rate; ASDR: age-standardized death rate; DALYs: disability-adjusted life years

**Fig. 2:**
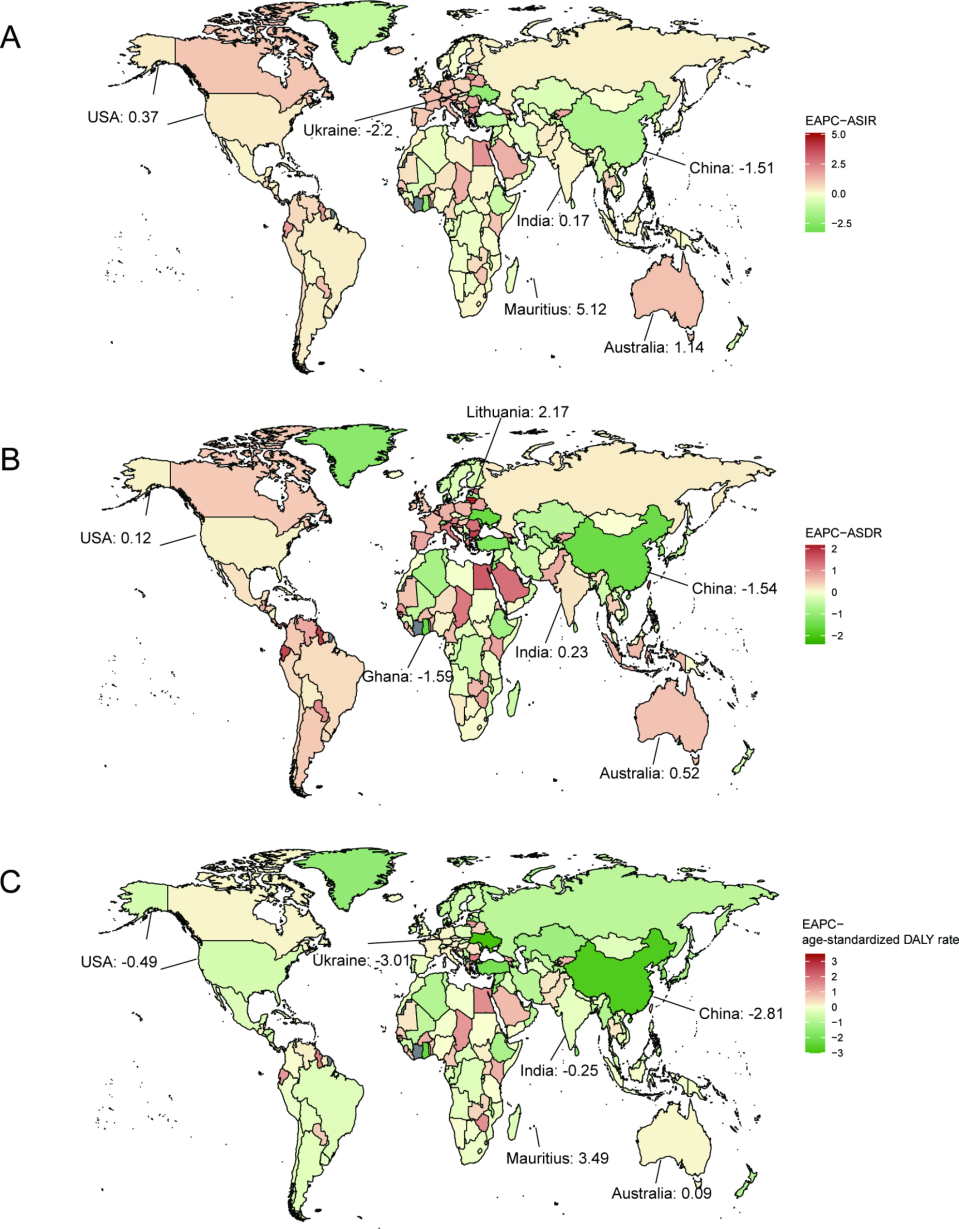
The global EAPCs of AML in 204 countries or territories in 2021: (**A**) The EAPCs of incidence in 2021; (**B**) the EAPCs of deaths in 2021; (**C**) the EAPCs of DALYs in 2021. AML: acute myeloid leukemia; DALYs: disability-adjusted life years; EAPCs: estimated annual percentage changes

**Fig. 3:**
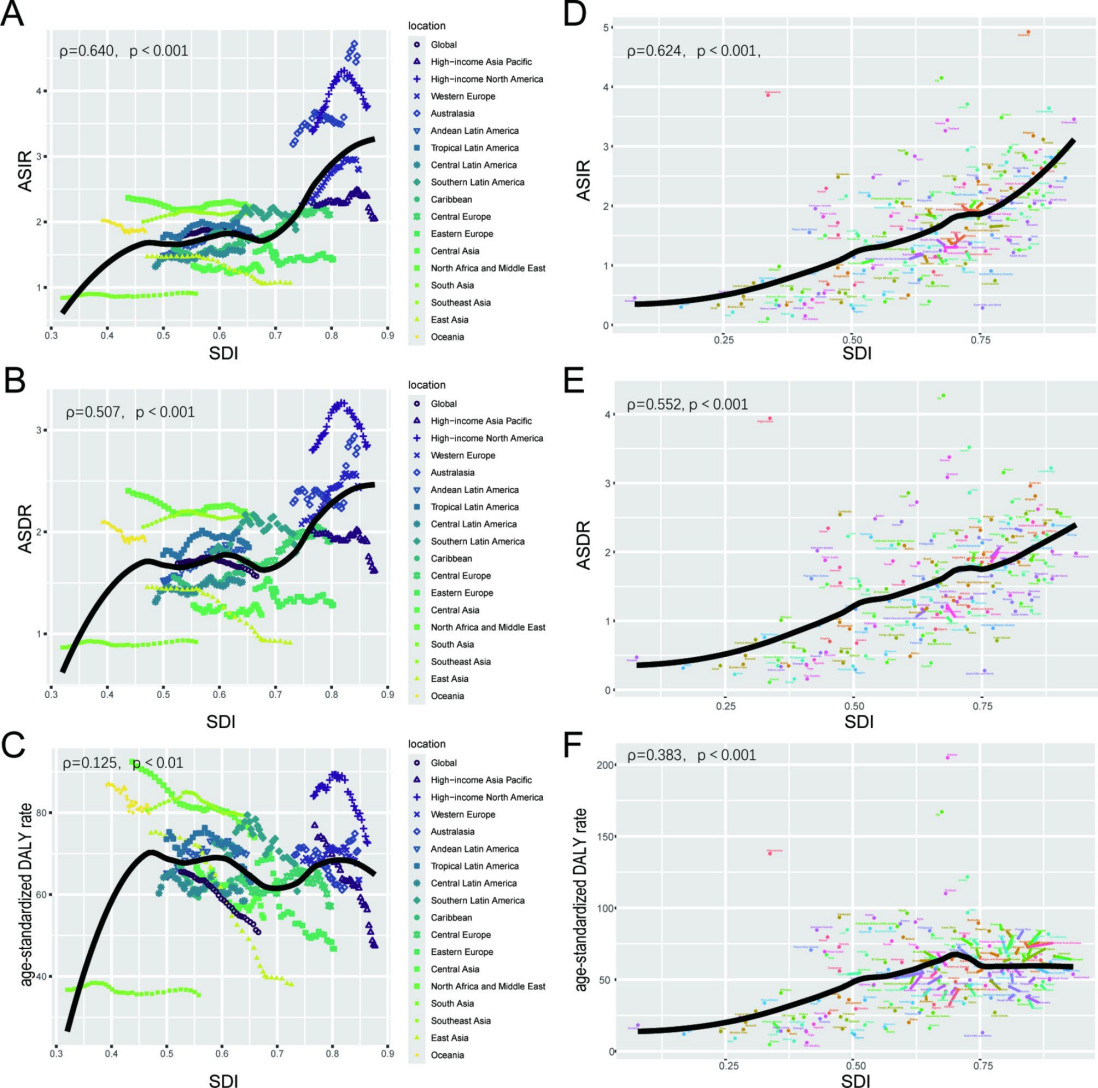
The change trends and correlation analyses of incidence rate and SDI from 1990 to 2021. (**A**) The change trends and correlation of ASIR and SDI from 1990 to 2021 in 18 regions. (**B**) The change trends and correlation of ASDR and SDI from 1990 to 2021 in 18 regions. (**C**) The change trends and correlation of age-standardized DALY rate and SDI from 1990 to 2021 in 18 regions. SDI, socio-demographic index. (**D**) The change trends and correlation of ASIR and SDI from 1990 to 2021 in 204 countries. (**E**) The change trends and correlation of ASDR and SDI from 1990 to 2021 in 204 countries. (**F**) The change trends and correlation of age-standardized DALY rate and SDI from 1990 to 2021 in 204 countries. SDI: socio-demographic index

The global incidence of AML increased from 79,372 in 1990 to 144,645 in 2021. However, the ASIR decreased from 1.77 per 100,000 persons in 1990 to 1.73 per 100,000 population in 2021. There has been an increase in incidence cases across all SDI quintiles, with the highest increase observed in the high SDI quintile at 1.04% (95%CI: -0.93 to 1.11). The high SDI quintile had the highest ASIR from 1990 to 2021, reaching 2.88 per 100,000 persons in 2021 and 2.65 per 100,000 persons in 1990. The ASIR exhibited an upward trend in the high and low-middle SDI quintiles while showed a decrease in the other three quintiles. Specifically, high SDI quintile had the most significant increase in ASIR with EAPC at 0.41 (95%CI: 0.25 to 0.56), whereas middle SDI quintile had the most significant decrease in ASIR with EAPC at -0.4 (95%CI: -0.46 to 0.34). Geographically, in 2021, High-income North America, with 23,676 cases, Western Europe, with 24,638 cases, and East Asia, with 19,156 cases, were the top 3 regions with the highest incidence cases. From 1990 to 2021, most regions showed an increasing trend in ASIR. Australasia (0.95 (95%CI: 0.69 to 1.21)), Central Europe (0.92 (95%CI: 0.78 to 1.07)), and Western Europe (0.87 (95%CI: 0.74 to 1.01)) exhibited the most significant increase, while East Asia had a noticeable decreasing trend in ASIR (-1.39 (95%CI: -1.57 to 1.2)). Furthermore, a significant positive correlation existed between SDI and ASIR (ρ = 0.640, *p*< 0.001) (Table 1). For country and regional observations, USA, China and India showed the highest disease burden, with 21,533, 17,835 and 11,040 incidence cases in 2021 (Table S1). Meanwhile, Australia recorded the highest ASIR at 4.9 per 100,000 persons in the same year (Fig. 1A). From 1990 to 2021, Mauritius showed the highest increase in ASIR with an EAPC of 5.12 (95%CI: 1.91 to 8.43), while the Northern Mariana Islands experienced the most significant decline with an EAPC of -3.23 (95%CI: -3.53 to -2.93) (Fig. 2A). China underwent an apparent decrease in ASIR with an EAPC of -1.51 (95%CI: -1.7 to -1.32), while USA(EAPC: 0.37 (95%CI: 0.14 to 0.61)) and India(EAPC:0.17 (95%CI: 0.07 to 0.27)) had an increase. Moreover, ASIR demonstrated an upward trend as the regional SDI increased (ρ = 0.624, *p*< 0.001) (Fig. 3A).

**Table 1:** The incidence cases and ASIR of AML in 1990 and 2021, and its temporal trends from 1990 to 2021

	Incident cases (95% CI)		Case change	ASIR (95% CI)		1990–2021 EAPCs (95% CI)
	1990	2021		1990	2021	
**Global**	79,372(62805–99428)	144,645 (126237–164851)	0.82% (0.46–1.12)	1.77 (1.43–2.16)	1.73 (1.51–1.98)	-0.03 (-0.12 to 0.06)
**SDI**						
High SDI	27,830 (26575–28799)	56,646 (51611–59717)	1.04% (0.93–1.11)	2.65 (2.54–2.75)	2.88 (2.66–3.02)	0.41 (0.25–0.56)
High-middle SDI	18,663 (14113–22745)	29,327 (24601–33129)	0.57% (0.28–0.92)	1.83 (1.39–2.23)	1.69 (1.4–1.92)	-0.31 (-0.38 to -0.23)
Middle SDI	20,167 (13539–29687)	33,429 (27391–41622)	0.66% (0.18–1.19)	1.41 (0.98–2.01)	1.29 (1.06–1.61)	-0.4 (-0.46 to -0.34)
Low-middle SDI	9631 (6241–15986)	19,061 (14795–25945)	0.98% (0.32–1.62)	1.06 (0.71–1.62)	1.17 (0.92–1.6)	0.34 (0.31–0.38)
Low SDI	2992 (1361–5453)	6027 (3672–7907)	1.01% (0.2–1.84)	0.81 (0.41–1.25)	0.79 (0.5–1.04)	-0.1 (-0.17 to -0.04)
**Central Europe**,** eastern Europe**,** and central Asia**						
Central Asia	861 (740–999)	1155 (1006–1351)	0.34% (0.15–0.6)	1.33 (1.16–1.54)	1.25 (1.09–1.46)	-0.05 (-0.15 to 0.06)
Central Europe	2515 (2348–2711)	4234 (3853–4594)	0.68% (0.55–0.83)	1.78 (1.66–1.91)	2.11 (1.92–2.29)	0.92 (0.78–1.07)
Eastern Europe	3824 (3527–4209)	4278 (3918–4654)	0.12% (-0.03 to 0.26)	1.54 (1.43–1.69)	1.43 (1.32–1.55)	-0.3 (-0.46 to -0.13)
**High income region**						
High-income Asia Pacific	4400 (4143–4754)	8361 (7251–9140)	0.9% (0.64–1.08)	2.28 (2.14–2.46)	2.05 (1.81–2.22)	-0.09 (-0.27 to 0.1)
High-income North America	11,533 (10888–11875)	23,676 (21534–24730)	1.05% (0.96–1.12)	3.38 (3.21–3.48)	3.77 (3.47–3.92)	0.42 (0.19–0.65)
Western Europe	11,791 (11278–12196)	24,638 (22110–26158)	1.09% (0.94–1.2)	2.25 (2.17–2.32)	2.79 (2.57–2.93)	0.87 (0.74–1.01)
Australasia	729 (682–781)	2333 (2074–2583)	2.2% (1.87–2.55)	3.19 (2.98–3.41)	4.53 (4.06–5.02)	0.95 (0.69–1.21)
**Latin America and Caribbean**						
Andean Latin America	452. (325–648)	1067 (744–1343)	1.36% (0.57–2.13)	1.49 (1.12–2.07)	1.72 (1.2–2.17)	0.8 (0.67–0.92)
Caribbean	494 (430–617)	840 (712–991)	0.7% (0.47–0.96)	1.59 (1.41–1.91)	1.65 (1.39–1.99)	0.31 (0.2–0.42)
Southern Latin America	872 (809–943)	1529 (1419–1664)	0.75% (0.58–0.93)	1.82 (1.69–1.97)	1.88 (1.75–2.05)	0.34 (0.12–0.56)
Tropical Latin America	2140 (2068–2218)	4630 (4336–4863)	1.16% (1.04–1.28)	1.76 (1.7–1.82)	1.86 (1.74–1.96)	0.3 (0.17–0.42)
Central Latin America	1779 (1706–1860.)	3946 (3515–4405)	1.22% (0.95–1.48)	1.32 (1.27–1.37)	1.56 (1.39–1.74)	0.4 (0.31–0.49)
**North Africa and Middle East**						
North Africa and Middle East	5642 (3743–8176)	11,358 (8396–15191)	1.01% (0.58–1.57)	2.37 (1.62–3.26)	2.22 (1.66-3)	-0.07 (-0.15 to 0.02)
**South Asia**						
South Asia	7185 (4451–11929)	14,665 (11306–20276)	1.04% (0.23–1.97)	0.84 (0.54–1.29)	0.91 (0.68–1.25)	0.15 (0.08–0.23)
**Southeast Asia**,** east Asia**,** and Oceania**						
East Asia	15,919 (8702–24823)	19,156 (13181–26300)	0.2% (-0.27 to 1.03)	1.47 (0.82–2.23)	1.07 (0.74–1.48)	-1.39 (-1.57 to -1.2)
Oceania	96 (47–133)	198 (103–280)	1.06% (0.62–1.53)	2.02 (0.98–2.81)	1.85 (0.95–2.63)	-0.28 (-0.35 to -0.21)
Southeast Asia	6957 (4745–10404)	14,234 (10092–17323)	1.05% (0.45–1.6)	2 (1.35–2.82)	2.13 (1.52–2.59)	0.12 (0.05–0.18)
**Sub-Saharan Africa**						
Central Sub-Saharan Africa	250 (125–431)	541 (297–750)	1.16% (0.26–2.32)	0.69 (0.35–1.01)	0.64 (0.37–0.91)	-0.23 (-0.32 to -0.13)
Eastern Sub-Saharan Africa	1011 (439–1819)	1882 (1009–2672)	0.86% (-0.11 to 2.03)	0.68 (0.32–1.05)	0.63 (0.36–0.89)	-0.33 (-0.39 to -0.26)
Southern Sub-Saharan Africa	440 (280–614)	889 (612–1236)	1.02% (0.68–1.44)	1.25 (0.81–1.75)	1.37 (0.92–1.88)	0.36 (0.2–0.52)
Western Sub-Saharan Africa	473 (227–868)	1026 (577–1352)	1.17% (0.35–1.93)	0.3 (0.16–0.45)	0.3 (0.19–0.38)	0.04 (-0.03 to 0.11)

### Deaths and DALYs burden of AML

The death cases also showed an upward trend globally, with 130,189 cases in 2021 and DALYs increased slightly to 4,135,056 in 2021. However, from 1990 to 2021, the global ASDR and age-standardized DALY rate showed a clear downward trend. At the SDI quintiles level, from 1990 to 2021, death cases and DALYs in all quintiles showed an upward trend, except for a slight decrease on DALYs in the high-middle SDI quintile (DALYs in 1990: 823,157; DALYs in 2021: 785,872). Moreover, except for an increase in ASDR in the low-middle SDI quintile (ASDR in 1990:1.09 (95%CI: 0.74 to 1.65); ASDR in 2021:1.18 (95%CI: 0.93 to 1.62)), the ASDR and age-standardized DALY rate of AML is significantly reduced in other quintiles. In 2021, the high SDI quintile had the highest death cases, ASDR, DALYs and age-standardized DALY rate, while the high-middle SDI quintile had the highest DALYs and age-standardized DALY rate in 1990. The ASDR for the high-middle SDI quintile declined significantly, with the EAPC of -0.36 (95%CI: -0.44 to -0.27) and the age-standardized DALY rate of -1.59 (95%CI: -1.7 to -1.48). As for geographical regions, Western Europe and High-income North America were the top two regions with the most death cases in 2021 (Western Europe: 22,485; High-income North American: 18,371). And the DALYs in East Asia were the highest in 2021 (586,467). At the same time, there was a statistically significant positive correlation between SDI, ASDR and age-standardized DALY rate (ASDR: ρ = 0.507, *p* < 0.001; age-standardized DALY rate: ρ = 0.125, *p* < 0.01) (Tables 2 and 3).

**Table 2:** The death cases and ASDR of AML in 1990 and 2021, and its temporal trends from 1990 to 2021

	Death cases (95% CI)		Case change	ASDR (95% CI)		1990–2021 EAPCs (95% CI)
	1990	2021		1990	2021	
**Global**	74,917 (58730–94714)	130,189 (113625–149385)	0.74% (0.39–1.03)	1.69 (1.36–2.08)	1.57 (1.37–1.8)	-0.15 (-0.2 to -0.1)
**SDI**						
High SDI	24,617 (23487–25533)	47,188 (42904–49837)	0.92% (0.81–0.99)	2.33 (2.22–2.41)	2.31 (2.13–2.43)	0.05 (-0.03 to 0.13)
High-middle SDI	17,930 (13566–21775)	26,275 (22369–29543)	0.47% (0.2–0.79)	1.77 (1.35–2.15)	1.49 (1.26–1.69)	-0.36 (-0.44 to -0.27)
Middle SDI	19,742 (13362–28731)	31,807 (26106–39669)	0.61% (0.15–1.12)	1.42 (1.01-2)	1.24 (1.02–1.55)	-0.36 (-0.42 to -0.29)
Low-middle SDI	9555 (6251–15762)	18,801 (14556–25892)	0.97% (0.33–1.61)	1.09 (0.74–1.65)	1.18 (0.93–1.62)	0.35 (0.32–0.38)
Low SDI	2987 (1371–5392)	5971 (3645–7889)	1% (0.2–1.82)	0.83 (0.43–1.29)	0.81 (0.51–1.08)	-0.14 (-0.17 to -0.1)
**Central Europe**,** eastern Europe**,** and central Asia**						
Central Asia	822 (708–956)	1080 (940–1269)	0.31% (0.12–0.57)	1.28 (1.12–1.48)	1.19 (1.04–1.39)	-0.28 (-0.36 to -0.2)
Central Europe	2446 (2287–2642)	3939 (3590–4275)	0.61% (0.48–0.75)	1.73 (1.61–1.86)	1.91 (1.74–2.08)	0.57 (0.48–0.65)
Eastern Europe	3586 (3306–3955)	3913 (3587–4265)	0.09% (-0.05 to 0.23)	1.44 (1.33–1.58)	1.28 (1.18–1.39)	-0.26 (-0.36 to -0.17)
**High income region**						
High-income Asia Pacific	3861 (3626–4208)	7072 (6112–7719)	0.83% (0.58-1)	2.01 (1.87–2.2)	1.62 (1.42–1.75)	-0.58 (-0.7 to -0.47)
High-income North America	9677 (9127–9984)	18,371 (16623–19211)	0.9% (0.81–0.95)	2.8 (2.66–2.89)	2.85 (2.61–2.97)	0.13 (0.02–0.25)
Western Europe	11,122 (10603–11519)	22,485 (20222–23904)	1.02% (0.88–1.13)	2.08 (1.99–2.14)	2.43 (2.23–2.56)	0.6 (0.52–0.68)
Australasia	525 (490–561)	1519 (1358–1668)	1.89% (1.62–2.18)	2.29 (2.13–2.43)	2.82 (2.55–3.09)	0.4 (0.23–0.58)
**Latin America and Caribbean**						
Andean Latin America	450 (326–637)	1037 (727–1306)	1.3% (0.55–2.05)	1.53 (1.15–2.09)	1.69 (1.19–2.13)	0.67 (0.59–0.75)
Central Latin America	1732 (1660–1809)	3792 (3378–4222)	1.19% (0.93–1.44)	1.31 (1.27–1.37)	1.51 (1.34–1.68)	0.39 (0.32–0.45)
Southern Latin America	861 (798–931)	1480 (1371–1609)	0.72% (0.55–0.9)	1.81 (1.67–1.96)	1.8 (1.67–1.96)	0.37 (0.22–0.52)
Tropical Latin America	2098 (2026–2175)	4589 (4273–4835)	1.19% (1.06–1.3)	1.78 (1.71–1.84)	1.85 (1.72–1.95)	0.3 (0.22–0.38)
**North Africa and Middle East**						
North Africa and Middle East	5540 (3722–7974)	10,653 (7890–14414)	0.92% (0.51–1.45)	2.4 (1.68–3.3)	2.15 (1.6–2.94)	-0.28 (-0.34 to -0.23)
**South Asia**						
South Asia	7145 (4461–11772)	14,565 (11115–20178)	1.04% (0.24–1.95)	0.87 (0.55–1.32)	0.92 (0.69–1.28)	0.23 (0.17–0.28)
**Southeast Asia**,** east Asia**,** and Oceania**						
East Asia	15,429 (8480–24004)	16,505 (11574–22715)	0.07% (-0.35 to 0.82)	1.45 (0.82–2.19)	0.91 (0.64–1.27)	-1.41 (-1.6 to -1.23)
Oceania	94 (45–132)	195 (101–277)	1.07% (0.63–1.55)	2.09 (1.01–2.93)	1.91 (0.97–2.73)	-0.28 (-0.33 to -0.24)
Southeast Asia	6870 (4692–10215)	13,883 (9869–16854)	1.02% (0.46–1.54)	2.05 (1.38–2.9)	2.12 (1.53–2.58)	0.25 (0.18–0.32)
**Sub-Saharan Africa**						
Eastern Sub-Saharan Africa	1007 (439–1805)	1858 (993–2669)	0.84% (-0.12 to 2)	0.7 (0.33–1.07)	0.64 (0.37–0.91)	-0.3 (-0.34 to -0.26)
Central Sub-Saharan Africa	249 (122–432)	532 (294–749)	1.14% (0.25–2.28)	0.72 (0.36–1.06)	0.65 (0.38–0.95)	-0.34 (-0.39 to -0.28)
Southern Sub-Saharan Africa	437 (279–612)	880 (602–1214)	1.01% (0.67–1.46)	1.28 (0.83–1.79)	1.39 (0.94–1.92)	0.23 (0.12–0.35)
Western Sub-Saharan Africa	475 (229–862)	1021 (570–1357)	1.15% (0.35–1.9)	0.32 (0.17–0.48)	0.31 (0.2–0.4)	0.06 (0.02–0.1)

**Table 3:** The DALYs and age-standardized DALY rate of AML in 1990 and 2021, and its temporal trends from 1990 to 2021

	DALYs (95% CI)			Age-standardized DALY rate (95% CI)		1990–2021 EAPCs (95% CI)
	1990	2021	Cases change	1990	2021	
**Global**	3,342,913 (2394171–4709119)	4,135,056 (3446487–4895237)	0.24% (-0.11 to 0.54)	65.55 (48.34–88.77)	50.79 (42.16–60.37)	-0.84 (-0.89 to -0.78)
**SDI**						
High SDI	740,178 (702920–769154)	1,032,409 (965057–1079433)	0.39% (0.32–0.45)	76.2 (71.83–79.48)	62.3 (58.11–64.96)	-0.51 (-0.62 to -0.4)
High-middle SDI	823,157 (577462–1089140)	785,872 (649177–901219)	-0.05% (-0.28 to 0.26)	79.8 (55.54–106.6)	51.86 (41.91–60.89)	-1.59 (-1.7 to -1.48)
Middle SDI	1,079,811 (697189–1664662)	1,194,869 (950401–1478134)	0.11% (-0.27 to 0.52)	63.93 (42.14–95.56)	47.54 (37.56–58.82)	-1.08 (-1.15 to -1.01)
Low-middle SDI	525,418 (334721–947996)	806,026 (618152–1096894)	0.53% (-0.06 to 1.15)	46.04 (29.78–76.64)	44.66 (34.15–60.88)	-0.04 (-0.08 to 0)
Low SDI	170,883 (75214–349811)	311,291 (182874–415147)	0.82% (0-1.82)	34.03 (15.84–59.9)	31.16 (18.99–41.21)	-0.31 (-0.37 to -0.25)
**Central Europe**,** eastern Europe**,** and central Asia**						
Central Asia	47,903 (40103–56170)	51,907 (44470–61107)	0.08% (-0.08 to 0.32)	66.45 (56.72–77.35)	54.29 (46.6-63.86)	-0.59 (-0.69 to -0.48)
Central Europe	84,817 (79025–90658)	98,146 (89395–106239)	0.16% (0.06–0.26)	63.42 (59.05–67.78)	57.15 (52.15–62.16)	-0.01 (-0.15 to 0.13)
Eastern Europe	154,572 (143854–170044)	121,067 (111218–131577)	-0.22% (-0.32 to -0.12)	67.73 (62.51–74.96)	46.75 (43.27–50.48)	-1.36 (-1.51 to -1.22)
**High income region**						
High-income Asia Pacific	141,027 (127597–154056)	145,958 (127828–157753)	0.03% (-0.11 to 0.12)	76.85 (68.39–84.42)	47.44 (41.64-51)	-1.32 (-1.45 to -1.18)
High-income North America	266,693 (257924–272157)	402,417 (377763–415932)	0.51% (0.45–0.55)	84.03 (81.65–85.57)	72.6 (68.88–74.87)	-0.45 (-0.62 to -0.28)
Western Europe	312,772 (302893–320972)	460,404 (427277–482067)	0.47% (0.39–0.54)	68.14 (66.39–69.69)	63.6 (60.57–66.12)	-0.04 (-0.15 to 0.07)
Australasia	15,273 (14500–16169)	32,225 (29401–35137)	1.11% (0.92–1.31)	69.63 (66.1-73.65)	70.69 (65.04–76.97)	-0.07 (-0.29 to 0.14)
**Latin America and Caribbean**						
Andean Latin America	24,723 (16757–36724)	40,588 (27527–50642)	0.64% (-0.02 to 1.25)	65.31 (46.24–93.31)	63.28 (42.95–79.01)	0.22 (0.07–0.37)
Southern Latin America	35,655 (33452–38325)	45,343 (42345–49082)	0.27% (0.16–0.41)	72.51 (68.03–78.01)	60.8 (56.76–65.99)	-0.33 (-0.52 to -0.13)
Tropical Latin America	104,943 (100363–109959)	152,687 (145511–159602)	0.45% (0.37–0.55)	73.52 (70.8-76.64)	63.07 (60.06–66.06)	-0.33 (-0.47 to -0.18)
Central Latin America	99,743 (94311–105474)	148,601 (131941–167202)	0.49% (0.31–0.68)	60.3 (57.67–63.19)	58.45 (51.73–65.95)	-0.18 (-0.27 to -0.08)
**North Africa and Middle East**						
North Africa and Middle East	279,114 (176664–427236)	422,232 (309214–574872)	0.51% (0.09–0.99)	92.41 (61.58–133.5)	73.97 (54.53-100.93)	-0.59 (-0.65 to -0.52)
**South Asia**						
South Asia	396,566 (237248–733586)	619,341 (460676–830678)	0.56% (-0.16 to 1.38)	36.76 (22.66–61.95)	35.44 (26.75–47.89)	-0.15 (-0.23 to -0.08)
**Southeast Asia**,** east Asia**,** and Oceania**						
East Asia	880,700 (442136–1483910)	586,467 (412970–820004)	-0.33% (-0.63 to 0.2)	75.01 (38.21-125.58)	38 (26.53–53.49)	-2.68 (-2.93 to -2.44)
Oceania	5480 (2669–7786)	10,743 (5836–14800)	0.96% (0.54–1.45)	86.71 (41.44-121.11)	79.86 (42.08-111.92)	-0.27 (-0.32 to -0.21)
Southeast Asia	343,604 (224321–572036)	537,644 (379223–664179)	0.56% (0.02–1.08)	80.56 (54.7-123.54)	77.3 (54.85–96.68)	-0.18 (-0.26 to -0.1)
**Sub-Saharan Africa**						
Central Sub-Saharan Africa	13,743 (6415–28270)	27,158 (14714–37579)	0.98% (0.04–2.29)	27.37 (13.58–45.72)	24.25 (13.5-34.31)	-0.3 (-0.39 to -0.21)
Eastern Sub-Saharan Africa	63,027 (26296–123767)	106,794 (56642–150397)	0.69% (-0.25 to 2.03)	30.72 (13.57–53.21)	26.61 (14.25–38.29)	-0.48 (-0.57 to -0.39)
Southern Sub-Saharan Africa	19,785 (12275–28762)	34,642 (23598–49750)	0.75% (0.41–1.15)	45.82 (28.77–65.49)	47.56 (32.42–67.27)	0.21 (0.03–0.38)
Western Sub-Saharan Africa	29,196 (12936–61930)	60,400 (31373–85648)	1.07% (0.18–1.93)	13.16 (6.48–23.27)	12.32 (7-16.31)	-0.08 (-0.17 to 0.02)

Regarding the observation of countries and territories, USA had the highest death cases in 2021 (16,648 (95%CI: 15097 to 17423)), while China had the highest DALYs (548,555 (95%CI: 373,859 to 778,262)) (Table S2, Table S3, Table S4). Meanwhile, Fiji had the highest ASDR with 4.3/100,000 persons, and Tokelau had the highest age-standardized DALY rate at 204.9/100,000 persons in 2021 (Fig. 1B, C). From 1990 to 2021, Lithuania (EAPC: 2.17 (95%CI: 1.94 to 2.4)) and Northern Mariana Islands (EAPC: -2.4 (95%CI: -2.7 to 2.09)) had the most increase and decline in ASDR, respectively. Mauritius (EAPC: 3.49 (95%CI: 0.82 to 6.22)) and Ukraine (EAPC: -3.01 (95%CI: -3.32 to 2.71)) had the most increase and decline in age-standardized DALY rate (Fig. 2B, C). In addition, correlation analysis found that as the SDI of countries and territories increased, their corresponding ASDR and age-standardized DALY rate would showed an upward trend (ASDR: ρ = 0.552, *p*< 0.001; age-standardized DALY rate: ρ = 0.383, *p*< 0.001) (Fig. 3B, C).

### Sex and age distribution of incidence, deaths and DALYs

Globally, both ASIR and ASDR have shown a fluctuating trend of first increasing and then decreasing from 1990 to 2021. Overall, the ASIR and ASDR of males are significantly higher than those of females. Meanwhile, age-standardized DALY rate showed a significant downward trend in both males and females.

**Fig. 4:**
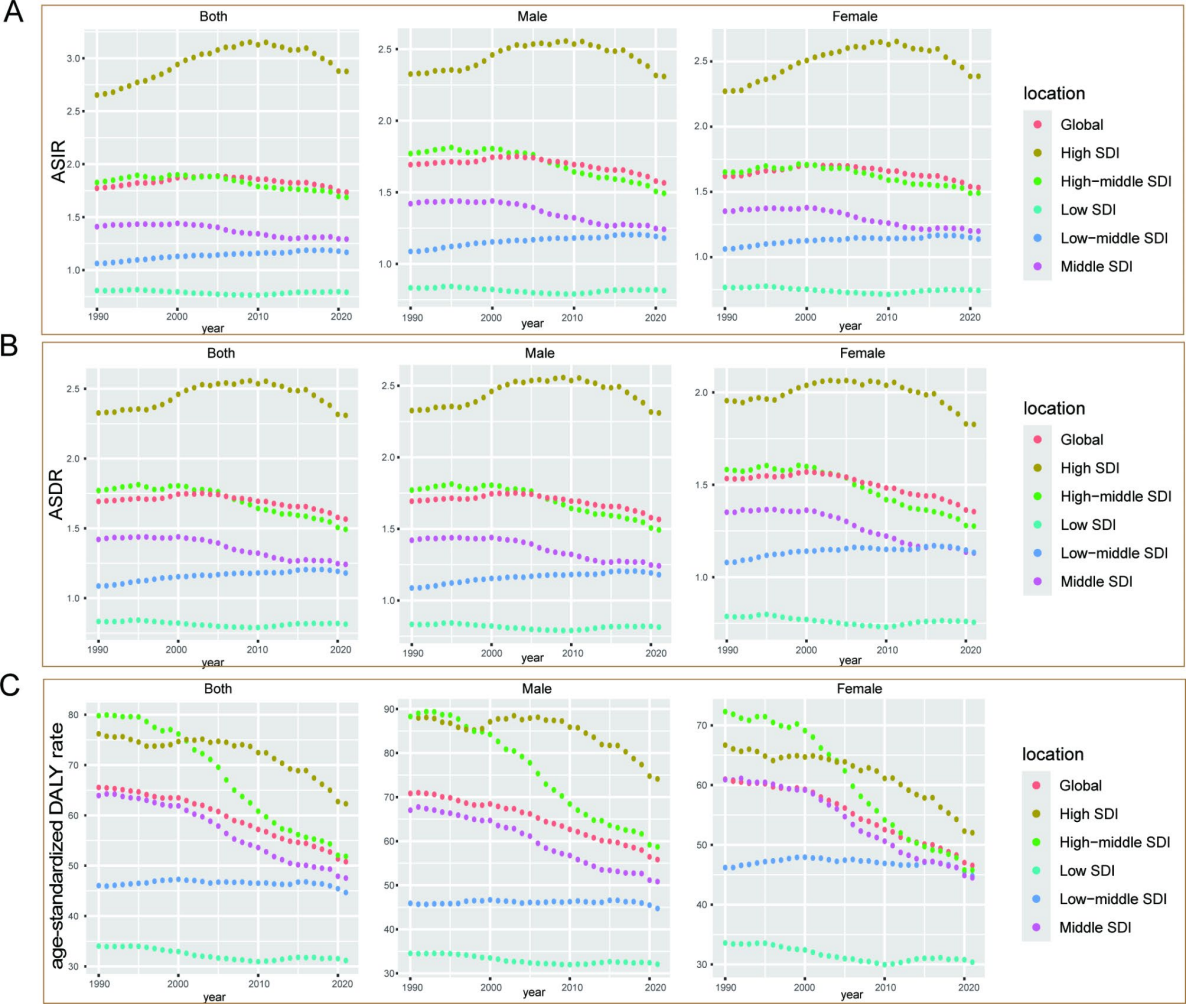
The change trends of age-standardized rates of AML among different SDI quintiles and sex: (**A**) the ASIR from 1990 to 2021; (**B**) The ASDR from 1990 to 2021; (**C**) The age-standardized DALY rate from 1990 to 2021. AML: acute myeloid leukemia; ASIR: age-standardized incidence rate; ASDR: age-standardized death rate; DALYs: disability-adjusted life years; SDI, socio-demographic index

For males, ASIR and ASDR were highest in the high SDI quintile and lowest in the low SDI quintile between 1990 and 2021. The difference in age-standardized DALY rate between the high SDI quintile and the high-middle SDI quintile was slight in 1990 but the decline from 1990 to 2021 was significantly greater in the high-middle SDI quintile than in the high SDI quintile. For females, the ASIR and ASDR were generally lower than those of males, but the trends from 1990 to 2021 were roughly the same as for males. In terms of age-standardized DALY rate in female, the highest age-standardized DALY rate was found in the high-middle quintile in 1990, but by 2021, the high SDI quintile has become the region with the highest age-standardized DALY rate. From 1990 to 2021, the number of age-standardized DALY rate for female declined significantly in all three quintiles except for the low SDI quintile and the low-middle quintile (Fig. 4).

**Fig. 5:**
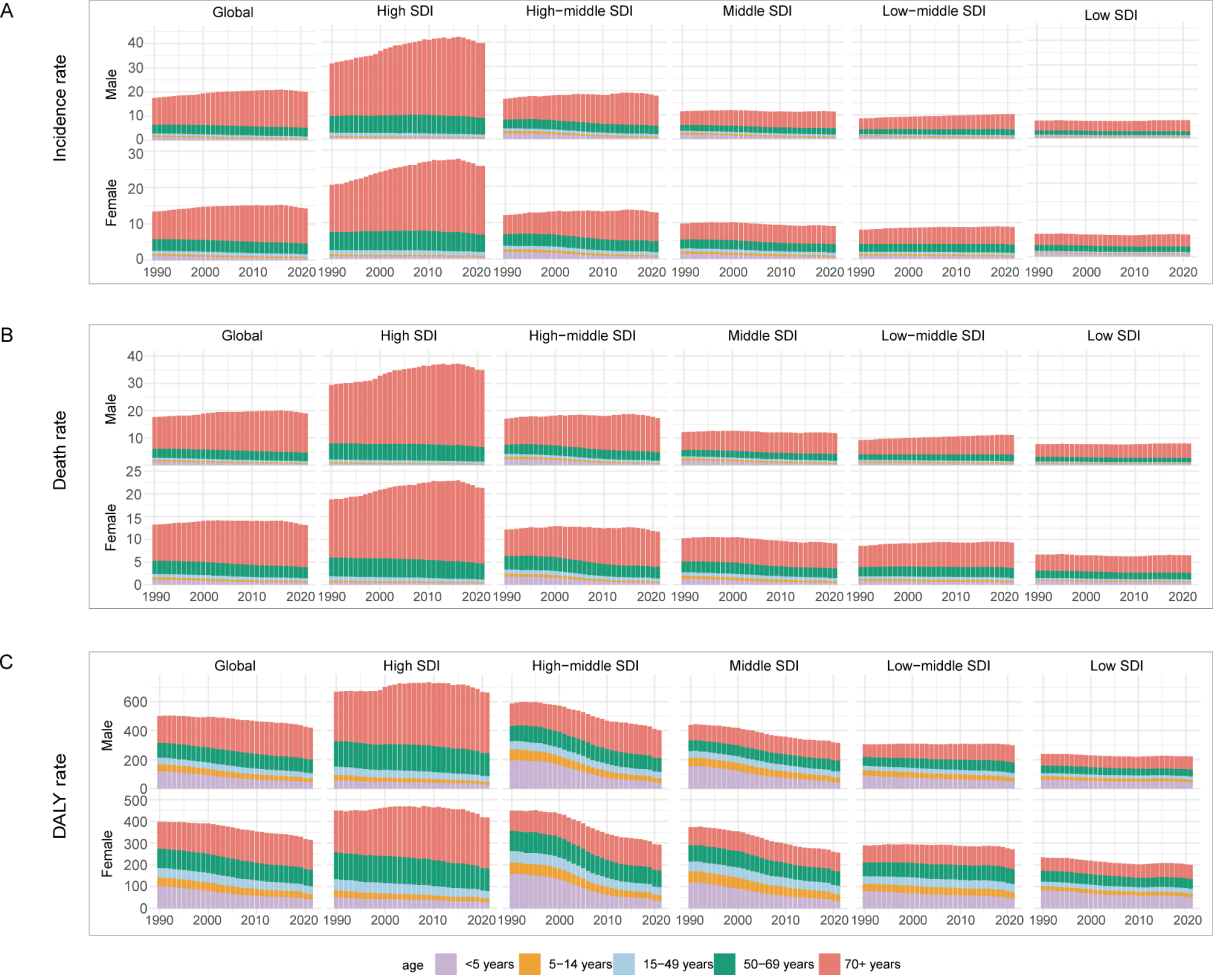
The incidence rate, death rate and DALY rates of AML of five age groups in different SDI quintiles from 1990 to 2021: (**A**) The incidence rate of AML of five age groups in different SDI quintiles from 1990 to 2021; (**B**) The death rate of AML of five age groups in different SDI quintiles from 1990 to 2021; (**C**) The DALY rate of AML of five age groups in different SDI quintiles from 1990 to 2021. The five age groups included < 5 years, 5–14 years, 15–49 years, 50–69 years, and 70 + years; SDI: socio-demographic index. The influential factors for EAPC

We categorized AML patients into five groups: <5 years, 5–14 years, 15–49 years, 50–69 years, and 70 + years. From 1990 to 2021, the incidence rates remained basically stable and the 70 + age group had the highest incidence rates. Death rates were highest among the 70 + age group in the high SDI quintile and among 15–49 years old in the low-middle SDI quintile. Moreover, death rates were higher among children (< 5 years, 5–14 years) in the low SDI quintile compared to other regions (Fig. 5A, B). Global DALY rates for males and females were decreasing, with the most significant declines in the high-middle SDI quintile (Fig. 5C). The number of incidence cases for both genders was rising, while males experienced a greater increase, especially in high-middle SDI countries and in the age group of 70 years and older (Figure S1). The number of death cases was also significantly higher in age groups above 70 years. Moreover, the number of deaths is higher in males than those in females despite age and SDI groups. As the population ages, the number of DALYs increased in older groups (50–69 and 70+).

**Fig. 6:**
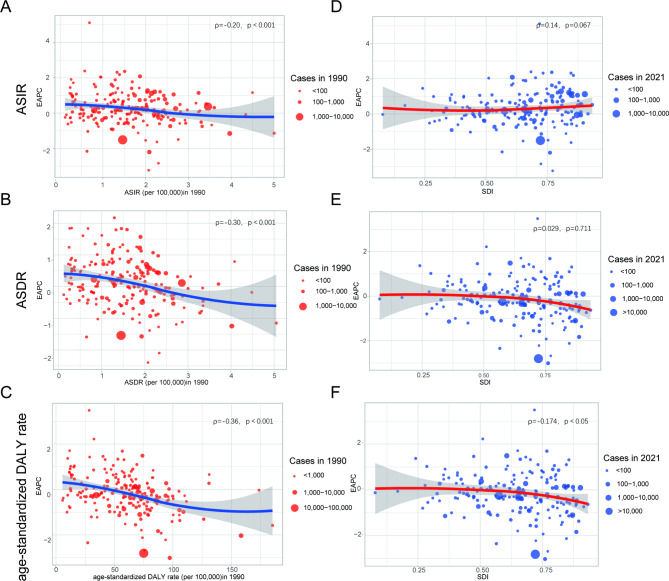
The correlation between EAPCs and age-standardized rate in 1990, and SDI in 2021: The correlation between EAPCs and ASIR in 1990 (**A**), and SDI in 2021 (**D**); The correlation between EAPCs and ASDR in 1990 (**B**), and SDI in 2021 (**E**); The correlation between EAPCs and age-standardized DALY rate in 1990 (**C**), and SDI in 2021 (**F**). The circles represent 204 countries or territories and the size of circle represents the number of AML patients ρ: Pearson’s correlation coefficient; AML: acute myeloid leukemia; ASIR: age-standardized incidence rate; ASDR: age-standardized death rate; DALYs: disability-adjusted life years; SDI: socio-demographic index; EAPCs: estimated annual percentage changes

We evaluated the correlation coefficient between the EAPC and ASR in 1990 and the SDI in 2021. We found that ASIR (ρ = − 0.20, *p*< 0.001), ASDR (ρ = − 0.30, *p*<0.001), and age-standardized DALY rate (ρ = − 0.36, *p*< 0.001) correlated negatively with the corresponding EAPC in 1990 (Fig. 6A, B, C). Meanwhile, correlations between SDI and EAPC of ASIR (ρ = 0.14, *p* = 0.067), ASDR (ρ = 0.029, *p* = 0.711), and age-standardized DALY rate (ρ = − 0.174, *p*< 0.05) were not significant (Fig. 6D, E, F). The results of these studies suggest that ASR in countries with a larger baseline disease burden in 1990 may show a more rapid downward trend. However, in 2021, countries with higher SDI will have an upward trend, which is consistent with the previous research results in different SDI regions.

### AML burden attributable to risk factors

**Fig. 7:**
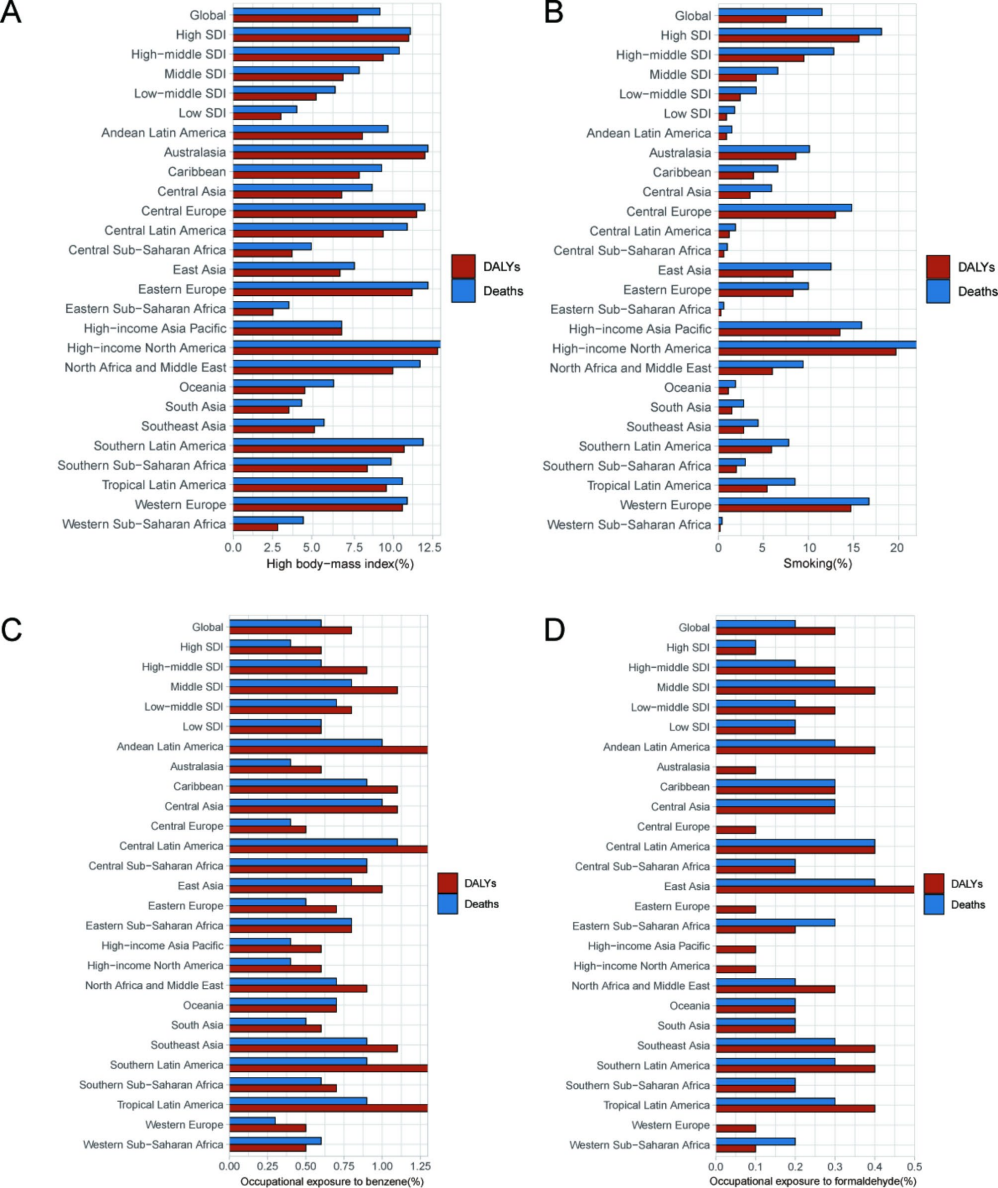
The proportions of four risk factors contributing patients with AML deaths and DALYs vary across the 27 global regions. (**A**) Proportion of high body mass index-related risk. (**B**) Proportion of smoking-associated risk. (**C**) Proportion of benzene occupational exposure-related risk. (**D**) Proportion of formaldehyde occupational exposure-related risk

From 1990 to 2021, smoking, high body mass index, and occupational exposure to benzene or formaldehyde were the most common potential risk factors related to AML in the GBD study, of which smoking was the most significant contributor. In 2021, Among the 21 geographical regions considered, High-income North America had the highest proportion of death cases and DALYs attributed to smoking, accounting for 22% and 19.70%. Conversely, Western Sub-Saharan Africa had the lowest percentages, with only 0.4% of death cases and 0.2% of DALYs (Fig. 7A). Meanwhile, High-income North America had the highest proportion of death cases and DALYs attributed to high BMI among the geographical regions, accounting for 13% and 12.8%. Conversely, Eastern Sub-Saharan Africa had the lowest percentages, with only 3.5% of death cases and 2.5% of DALYs associated with high BMI (Fig. 7B). Among the countries and territories considered, Andean Latin America had the highest proportion of DALYs attributed to occupational exposure to benzene, accounting for 1.3%. Central Latin America had the highest proportion of deaths attributed to occupational exposure to benzene, accounting for 1.1% and Western Europe had the lowest proportion of death cases, only 0.3%. Meanwhile, Central Europe had the lowest proportion of DALYs attributed to occupational exposure to benzene, accounting for 0.5% (Fig. 7C). In 2021, Among the 21 geographical regions considered, East Asia had the highest proportion of death cases and DALYs attributed to occupational exposure to formaldehyde, accounting for 0.4% and 0.5% (Fig. 7D).

## Discussion

AML is a prevalent hematological malignancy characterized by rapid disease progression, acquired treatment resistance, and frequent relapses [29, 30]. It poses complex challenges to global public health, spurring ongoing discussions regarding its worldwide burden [23, 31, 32]. In this study, we explore the disease burden across various populations and periods using the latest GBD database and assess the epidemiological trends in AML through EAPC calculations over the past 32 years. From 1990 to 2021, the incidence cases of AML surged from 79,372 to 144,645, reflecting a significant global increase. The result potentially attributed to population expansion, aging, and enhanced diagnostic technologies [33]. Furthermore, there was a global increase in AML-related deaths, while the ASDR and age-standardized DALYs were decreasing. The rise in fatalities may be associated with the growing number of cases, but the declining ASDR suggested that there may have been improvements in the medical treatment and management of AML patients. Similarly, the reduction in age-standardized DALYs implied a decrease in disease burden per unit of the population. In addition, we also examined the relationship between the incidence, mortality, and DALYs of AML and SDI. We found that the incidence increased the most and the ASIR was the highest in high-SDI quintile, which may be related to the improvement of diagnostic efficiency. In addition, factors such as increased environmental pollution, faster pace of life, greater mental stress, and widespread obesity in high-SDI quintile may also lead to increased incidence [34]. In addition, the number of deaths and DALYs in high-SDI quintile was the highest, and their corresponding ASDR and age-standardized DALYs showed an upward trend. This result may be related to the aggravation of the aging of the population in these regions. The increase in ASIR in middle and low SDI quintile may be related to lifestyle, genetic factors, and environmental factors. The ASDR increased in middle and low SDI quintile, but it decreased in other SDI quintiles, this phenomenon may reflect the lack of medical resources in middle and low SDI quintiles [35]. The decrease of incidence in low SDI quintile may be related to low diagnostic efficiency and incomplete data collection. Among them, both ASIR and ASDR in East Asia have decreased significantly, reflecting the continuous improvement of public health measures and medical care systems in the region. The occurrence and death rates of AML globally show intricate patterns, influenced by various factors such as population demographics, economic advancement, and healthcare quality [36]. Tailored prevention and control measures are essential for different SDI quintiles to lessen the overall impact of AML on a global scale.

The escalating global burden of AML attributable to population ageing is multifaceted, encompassing not only an increase in the incidence rate but also numerous challenges associated with its therapeutic management [4, 37]. Our findings indicate that across all geographical regions, there is a concomitant increase in the incidence rate, death, and DALYs for AML with growing age. Notably, the incidence of AML climbs with age, and the death rate for patients diagnosed beyond the age of 65 exceeds 90% [3]. Ageing is a pivotal factor contributing to the pathogenesis of leukemia, with the accumulation of environmental exposures over time, the build-up of genetic mutations, immune system dysregulation, aberrant bone marrow hematopoiesis, and heightened levels of chronic inflammation [38–40] all of which incrementing the risk of AML. This is intrinsically linked to the association between AML incidence and age-related clonal hematopoiesis [41, 42]. Current AML treatment guidelines recommend the high-dose cytarabine consolidation chemotherapy or allogeneic stem cell transplantation (Allo-HSCT) based on the individual’s disease risk profile to mitigate the risk of relapse [11]. However, the efficacy of this therapeutic approach is limited, with a curative rate ranging from approximately 35–45% in patients under 60, while in those over 60, the cure rate diminishes to less than 15% [43]. On one front, the combination of demethylating agents with the BCL-2 inhibitor Venetoclax has demonstrated benefit in the elderly and those unsuitable for intensive therapy [44, 45], and a multitude of clinical trials are investigating the safety and efficacy of multi-targeted drug combinations for the treatment of relapsed and refractory AML [46, 47]. On the other hand, over the past five decades, the pace of progress in AML research and treatment has been relatively modest. The intensified induction regimen comprising cytarabine and anthracycline drugs, commonly referred to as the “7 + 3 regimen,” which was introduced in the 1970s for AML treatment, remains the cornerstone of therapy [48]. Nonetheless, chemotherapy resistance, disease recurrence, and limited therapeutic options for elderly or ineligible patients for intensive treatment persist as formidable challenges in the management of AML [49, 50].

Our study also investigated potential risk factors for AML-related death [51]. Lifestyle, occupational exposure, and carcinogens in the living environment are closely related to the risk of AML [52]. Specifically, high body mass index (BMI), occupational exposure to benzene, formaldehyde, and smoking are key factors in the occurrence of AML [53]. Overweight and obesity significantly increase AML risk, with a meta-analysis has demonstrated that individuals with overweight and obesity are at a 23% higher risk of developing AML compared to normal-weight individuals [54]. However, high BMI does not significantly impact the overall survival or disease-free survival in non-APL AML patients. Occupational exposure to formaldehyde and benzene also significantly raises AML risk [55, 56]. For instance, a study assessed the risk of death from AML and other lymphohematopoietic malignancies due to formaldehyde exposure and found an increased risk associated with certain types of lymphohematopoietic malignancies. However, there is no significant correlation with AML itself [57]. Another study evaluated the cancer risk from formaldehyde exposure within anatomical laboratory settings, revealing that the assessed cancer risk for the staff exceeded the limits recommended by the United States Environmental Protection Agency (EPA) [58]. Regarding the relationship between occupational exposure to benzene and AML, current research has not definitively established a direct link between benzene exposure and AML. However, as a known carcinogen, benzene remains a significant consideration in the occupational environment for the incidence of AML [23]. Smoking is a well-documented risk factor with multiple meta-analyses indicating an increased risk of AML [59]. For example, a systematic review and meta-analysis included 20 case-control studies involving 7,538 AML patients and 137,924 healthy controls [60]. Another updated meta-analysis covered 23 studies published from 1993 to 2013, involving 7,746 cases of AML [61]. Additionally, a pooled analysis of nine cohort studies conducted in Japan showed an increased risk of AML associated with smoking [62]. In conclusion, high BMI, occupational exposure to formaldehyde and benzene, and smoking are pivotal risk factors for the incidence and mortality of AML. These factors are particularly evident in developed regions with high AML morbidity and mortality. Consequently, effective intervention strategies targeting these risk factors are imperative for reducing the burden of AML.

Our research also has several limitations. Firstly, clinical processes such as data collection and patient care in AML in 2020–2021 may be affected due to the COVID-19 pandemic. The GBD research process corrects for these potential limitations through a variety of methods, such as the use of modelling to estimate missing data or adjusting analytical strategies to account for the influence of the epidemic [63]. However, these adjustments may themselves introduce a degree of uncertainty. While the GBD database encompasses many countries and regions globally, the accuracy and completeness of data may differ across regions. Particularly in low- and middle-income countries, the data may be less detailed or subject to bias. The classification and definitions of diseases in the GBD database may differ from those in other international databases or studies, potentially compromising the accuracy of disease burden assessments. Furthermore, while data from various periods and regions may be influenced by differing medical practices, diagnostic techniques, and reporting systems, leading to biases when analyzing long-term trends. Additionally, while the GBD database employs various epidemiological indicators to assess disease burden comprehensively, these indicators may not fully capture the complete impact of AML on patients’ quality of life and socioeconomic status. Finally, the GBD database offers population-level data, which may not include detailed individual patient information, thus limiting in-depth analysis of the epidemiological characteristics of specific subtypes or patient groups in AML.

## Conclusion

This study reveals that from 1990 to 2021, there was a rising trend in the incidence and deaths of AML globally, with a more pronounced disease burden observed in males and the elderly population. We found a positive correlation between the incidence of AML and SDI values. Smoking, high body mass index (BMI), and occupational exposure to benzene and formaldehyde are the main risk factors for AML-related deaths. Smoking and high body mass index predominated in developed countries, while occupational exposure was the predominant risk factor in developing countries. These findings underscore the importance of developing preventive measures tailored to different regions and populations. Future research should focus on developing effective prevention strategies and exploring novel therapeutic approaches to reduce the incidence of AML and improve patient prognosis.

## Electronic Supplementary Material

Below is the link to the electronic supplementary material.

Supplementary Material 1

Supplementary Material 2

Supplementary Material 3

Supplementary Material 4

Supplementary Material 5

## Data Availability

No datasets were generated or analysed during the current study.

## References

[1] Papaemmanuil E, Gerstung M, Bullinger L, Gaidzik VI, Paschka P, Roberts ND, et al. Genomic classification and prognosis in acute myeloid leukemia. N Engl J Med. 2016;374:2209–21. 27276561 10.1056/NEJMoa1516192 PMC4979995

[2] DiNardo CD, Erba HP, Freeman SD, Wei AH. Acute myeloid leukaemia. Lancet. 2023;401:2073–86. 37068505 10.1016/S0140-6736(23)00108-3

[3] Abelson S, Collord G, Ng SWK, Weissbrod O, Mendelson Cohen N, Niemeyer E, et al. Prediction of acute myeloid leukaemia risk in healthy individuals. Nature. 2018;559:400–4. 29988082 10.1038/s41586-018-0317-6 PMC6485381

[4] Komp-Leukkunen K, Sarasma J. Social sustainability in aging populations: a systematic literature review. Gerontologist. 2024;64. 10.1093/geront/gnad097 PMC11036160 37526564

[5] Kristensen D, Nielsen LB, Roug AS, Kristensen TC, El-Galaly TC, Nørgaard JM, et al. The prognostic effect of smoking status on intensively treated acute myeloid leukaemia - a Danish nationwide cohort study. Br J Haematol. 2020;190:236–43. 32316076 10.1111/bjh.16667 PMC7496881

[6] Castillo JJ, Reagan JL, Ingham RR, Furman M, Dalia S, Merhi B, et al. Obesity but not overweight increases the incidence and mortality of leukemia in adults: a meta-analysis of prospective cohort studies. Leuk Res. 2012;36:868–75. 22285508 10.1016/j.leukres.2011.12.020

[7] Khalade A, Jaakkola MS, Pukkala E, Jaakkola JJ. Exposure to benzene at work and the risk of leukemia: a systematic review and meta-analysis. Environ Health. 2010;9:31. 20584305 10.1186/1476-069X-9-31 PMC2903550

[8] Lan Q, Smith MT, Tang X, Guo W, Vermeulen R, Ji Z, et al. Chromosome-wide aneuploidy study of cultured circulating myeloid progenitor cells from workers occupationally exposed to formaldehyde. Carcinogenesis. 2015;36:160–7. 25391402 10.1093/carcin/bgu229 PMC4291049

[9] Yu Z, Li J, Wen X, Han Y, Jiang P, Zhu M, et al. AMLnet, a deep-learning pipeline for the differential diagnosis of acute myeloid leukemia from bone marrow smears. J Hematol Oncol. 2023;16:27. 36945063 10.1186/s13045-023-01419-3 PMC10031907

[10] Arber DA, Orazi A, Hasserjian R, Thiele J, Borowitz MJ, Le Beau MM, et al. The 2016 revision to the World Health Organization classification of myeloid neoplasms and acute leukemia. Blood. 2016;127:2391–405. 27069254 10.1182/blood-2016-03-643544

[11] Döhner H, Wei AH, Appelbaum FR, Craddock C, DiNardo CD, Dombret H, et al. Diagnosis and management of AML in adults: 2022 recommendations from an international expert panel on behalf of the ELN. Blood. 2022;140:1345–77. 35797463 10.1182/blood.2022016867

[12] Yilmaz M, Kantarjian H, Ravandi F. Acute promyelocytic leukemia current treatment algorithms. Blood Cancer J. 2021;11:123. 34193815 10.1038/s41408-021-00514-3 PMC8245494

[13] Kottaridis PD, Gale RE, Frew ME, Harrison G, Langabeer SE, Belton AA, et al. The presence of a FLT3 internal tandem duplication in patients with acute myeloid leukemia (AML) adds important prognostic information to cytogenetic risk group and response to the first cycle of chemotherapy: analysis of 854 patients from the United Kingdom Medical Research Council AML 10 and 12 trials. Blood. 2001;98:1752–9. 11535508 10.1182/blood.V98.6.1752

[14] Stirewalt DL, Kopecky KJ, Meshinchi S, Appelbaum FR, Slovak ML, Willman CL, Radich JP. FLT3, RAS, and TP53 mutations in elderly patients with acute myeloid leukemia. Blood. 2001;97:3589–95. 11369655 10.1182/blood.V97.11.3589

[15] Grob T, Sanders MA, Vonk CM, Kavelaars FG, Rijken M, Hanekamp DW, et al. Prognostic value of FLT3-internal tandem duplication residual disease in acute myeloid leukemia. J Clin Oncol. 2023;41:756–65. 36315929 10.1200/JCO.22.00715 PMC9901965

[16] Bazinet A, Kantarjian HM. Moving toward individualized target-based therapies in acute myeloid leukemia. Ann Oncol. 2023;34:141–51. 36423744 10.1016/j.annonc.2022.11.004

[17] DiNardo KW, LeBlanc TW, Chen H. Novel agents and regimens in acute myeloid leukemia: latest updates from 2022 ASH annual meeting. J Hematol Oncol. 2023;16:17. 10.1186/s13045-023-01411-x PMC9983204 36869366

[18] Wienecke CP, Heida B, Venturini L, Gabdoulline R, Krüger K, Teich K et al. Clonal relapse dynamics in acute myeloid leukemia following allogeneic hematopoietic cell transplantation. Blood. 2024. 10.1182/blood.2023022697 38669617

[19] Forsberg M, Konopleva M. AML treatment: conventional chemotherapy and emerging novel agents. Trends Pharmacol Sci. 2024;45:430–48. 38643058 10.1016/j.tips.2024.03.005

[20] Ning L, Li D, Lu P, Que Y. Exploring the determinants that influence hospital costs of induction therapy for acute myeloid leukemia. Leuk Lymphoma. 2021;62:1211–8. 33300383 10.1080/10428194.2020.1855339

[21] Peseski AM, McClean M, Green SD, Beeler C, Konig H. Management of fever and neutropenia in the adult patient with acute myeloid leukemia. Expert Rev Anti Infect Ther. 2021;19:359–78. 32892669 10.1080/14787210.2020.1820863

[22] Mittelman M, Platzbecker U, Afanasyev B, Grosicki S, Wong RSM, Anagnostopoulos A, et al. Eltrombopag for advanced myelodysplastic syndromes or acute myeloid leukaemia and severe thrombocytopenia (ASPIRE): a randomised, placebo-controlled, phase 2 trial. Lancet Haematol. 2018;5:e34–43. 29241762 10.1016/S2352-3026(17)30228-4

[23] Yi M, Li A, Zhou L, Chu Q, Song Y, Wu K. The global burden and attributable risk factor analysis of acute myeloid leukemia in 195 countries and territories from 1990 to 2017: estimates based on the global burden of disease study 2017. J Hematol Oncol. 2020;13:72. 32513227 10.1186/s13045-020-00908-z PMC7282046

[24] Jani CT, Ahmed A, Singh H, Mouchati C, Al Omari O, Bhatt PS, et al. Burden of AML, 1990–2019: estimates from the global burden of disease study. JCO Glob Oncol. 2023;9:e2300229. 37992271 10.1200/GO.23.00229 PMC10681472

[25] Global burden. Of 288 causes of death and life expectancy decomposition in 204 countries and territories and 811 subnational locations, 1990–2021: a systematic analysis for the global burden of disease study 2021. Lancet. 2024;403:2100–32. 38582094 10.1016/S0140-6736(24)00367-2 PMC11126520

[26] Global incidence. Prevalence, years lived with disability (YLDs), disability-adjusted life-years (DALYs), and healthy life expectancy (HALE) for 371 diseases and injuries in 204 countries and territories and 811 subnational locations, 1990–2021: a systematic analysis for the global burden of disease study 2021. Lancet. 2024;403:2133–61. 38642570 10.1016/S0140-6736(24)00757-8 PMC11122111

[27] Global burden and strength of evidence for. 88 risk factors in 204 countries and 811 subnational locations, 1990–2021: a systematic analysis for the global burden of disease study 2021. Lancet. 2024;403:2162–203. 38762324 10.1016/S0140-6736(24)00933-4 PMC11120204

[28] Fitzmaurice C, Allen C, Barber RM, Barregard L, Bhutta ZA, Brenner H, et al. Global, regional, and national cancer incidence, mortality, years of life lost, years lived with disability, and disability-adjusted life-years for 32 cancer groups, 1990 to 2015: a systematic analysis for the global burden of disease study. JAMA Oncol. 2017;3:524–48. 27918777 10.1001/jamaoncol.2016.5688 PMC6103527

[29] Ogana HA, Hurwitz S, Wei N, Lee E, Morris K, Parikh K, Kim YM. Targeting integrins in drug-resistant acute myeloid leukaemia. Br J Pharmacol. 2024;181:295–316. 37258706 10.1111/bph.16149

[30] Herrity E, Pereira MP, Kim DDH. Acute myeloid leukaemia relapse after allogeneic haematopoietic stem cell transplantation: mechanistic diversity and therapeutic directions. Br J Haematol. 2023;203:722–35. 37787151 10.1111/bjh.19121

[31] Chen P, Liu X, Zhao Y, Hu Y, Guo J, Wang H. Global, national, and regional burden of acute myeloid leukemia among 60–89 years-old individuals: insights from a study covering the period 1990 to 2019. Front Public Health. 2023;11:1329529. 38274540 10.3389/fpubh.2023.1329529 PMC10808630

[32] Ou Z, Yu D, Liang Y, He W, Li Y, Zhang M, et al. Analysis of the global burden of disease study highlights the trends in death and disability-adjusted life years of leukemia from 1990 to 2017. Cancer Commun (Lond). 2020;40:598–610. 32936522 10.1002/cac2.12094 PMC7668511

[33] Shallis RM, Wang R, Davidoff A, Ma X, Zeidan AM. Epidemiology of acute myeloid leukemia: recent progress and enduring challenges. Blood Rev. 2019;36:70–87. 31101526 10.1016/j.blre.2019.04.005

[34] Kasim K, Levallois P, Abdous B, Auger P, Johnson KC. Lifestyle factors and the risk of adult leukemia in Canada. Cancer Causes Control. 2005;16:489–500. 15986104 10.1007/s10552-004-7115-1

[35] Byrne MM, Halman LJ, Koniaris LG, Cassileth PA, Rosenblatt JD, Cheung MC. Effects of poverty and race on outcomes in acute myeloid leukemia. Am J Clin Oncol. 2011;34:297–304. 20562587 10.1097/COC.0b013e3181dea934

[36] Guadamuz JS, Wang X, Ryals CA, Miksad RA, Snider J, Walters J, Calip GS. Socioeconomic status and inequities in treatment initiation and survival among patients with cancer, 2011–2022. JNCI Cancer Spectr. 2023;7. 10.1093/jncics/pkad058 PMC10582690 37707536

[37] Mahmood MN, Dhakal SP. Ageing population and society: a scientometric analysis. Qual Quant. 2022:1–18. 10.1007/s11135-022-01509-3 PMC9403228 36039154

[38] Onyije FM, Olsson A, Erdmann F, Magnani C, Petridou E, Clavel J, et al. Parental occupational exposure to combustion products, metals, silica and asbestos and risk of childhood leukaemia: findings from the Childhood Cancer and Leukaemia International Consortium (CLIC). Environ Int. 2022;167:107409. 35908390 10.1016/j.envint.2022.107409 PMC9376807

[39] Liao M, Chen R, Yang Y, He H, Xu L, Jiang Y, et al. Aging-elevated inflammation promotes DNMT3A R878H-driven clonal hematopoiesis. Acta Pharm Sin B. 2022;12:678–91. 35256939 10.1016/j.apsb.2021.09.015 PMC8897035

[40] Belizaire R, Wong WJ, Robinette ML, Ebert BL. Clonal haematopoiesis and dysregulation of the immune system. Nat Rev Immunol. 2023;23:595–610. 36941354 10.1038/s41577-023-00843-3 PMC11140722

[41] Hasserjian RP, Steensma DP, Graubert TA, Ebert BL. Clonal hematopoiesis and measurable residual disease assessment in acute myeloid leukemia. Blood. 2020;135:1729–38. 32232484 10.1182/blood.2019004770 PMC7225688

[42] Desai P, Hassane D, Roboz GJ. Clonal hematopoiesis and risk of acute myeloid leukemia. Best Pract Res Clin Haematol. 2019;32:177–85. 31203999 10.1016/j.beha.2019.05.007

[43] Short NJ, Konopleva M, Kadia TM, Borthakur G, Ravandi F, DiNardo CD, Daver N. Advances in the treatment of acute myeloid leukemia: new drugs and new challenges. Cancer Discov. 2020;10:506–25. 32014868 10.1158/2159-8290.CD-19-1011

[44] Morsia E, McCullough K, Joshi M, Cook J, Alkhateeb HB, Al-Kali A, et al. Venetoclax and hypomethylating agents in acute myeloid leukemia: Mayo Clinic series on 86 patients. Am J Hematol. 2020;95:1511–21. 32833294 10.1002/ajh.25978

[45] Pollyea DA, Winters A, McMahon C, Schwartz M, Jordan CT, Rabinovitch R, et al. Venetoclax and azacitidine followed by allogeneic transplant results in excellent outcomes and may improve outcomes versus maintenance therapy among newly diagnosed AML patients older than 60. Bone Marrow Transpl. 2022;57:160–6. 10.1038/s41409-021-01476-7 34645926

[46] Daver NG, Dail M, Garcia JS, Jonas BA, Yee KWL, Kelly KR, et al. Venetoclax and idasanutlin in relapsed/refractory AML: a nonrandomized, open-label phase 1b trial. Blood. 2023;141:1265–76. 36265087 10.1182/blood.2022016362 PMC10651777

[47] Duong VH, Ruppert AS, Mims AS, Borate U, Stein EM, Baer MR, et al. Entospletinib with decitabine in acute myeloid leukemia with mutant TP53 or complex karyotype: a phase 2 substudy of the beat AML master trial. Cancer. 2023;129:2308–20. 37078412 10.1002/cncr.34780 PMC11225573

[48] Kantarjian HM, Kadia TM, DiNardo CD, Welch MA, Ravandi F. Acute myeloid leukemia: treatment and research outlook for 2021 and the MD Anderson approach. Cancer. 2021;127:1186–207. 33734442 10.1002/cncr.33477 PMC12084862

[49] Gurnari C, Pagliuca S, Visconte V. Deciphering the therapeutic resistance in acute myeloid leukemia. Int J Mol Sci. 2020;21. 10.3390/ijms21228505 PMC7697160 33198085

[50] Zhang J, Gu Y, Chen B. Mechanisms of drug resistance in acute myeloid leukemia. Onco Targets Ther. 2019;12:1937–45. 30881045 10.2147/OTT.S191621 PMC6417008

[51] Shallis RM, Weiss JJ, Deziel NC, Gore SD. Challenging the concept of de novo acute myeloid leukemia: environmental and occupational leukemogens hiding in our midst. Blood Rev. 2021;47:100760. 32988660 10.1016/j.blre.2020.100760

[52] Colamesta V, D’Aguanno S, Breccia M, Bruffa S, Cartoni C, La Torre G. Do the smoking intensity and duration, the years since quitting, the methodological quality and the year of publication of the studies affect the results of the meta-analysis on cigarette smoking and acute myeloid leukemia (AML) in adults? Crit Rev Oncol Hematol. 2016;99:376–88. 26830008 10.1016/j.critrevonc.2016.01.003

[53] Cox LA Jr., Thompson WJ, Mundt KA. Interventional probability of causation (IPoC) with epidemiological and partial mechanistic evidence: benzene vs. formaldehyde and acute myeloid leukemia (AML). Crit Rev Toxicol. 2024;54:252–89. 38753561 10.1080/10408444.2024.2337435

[54] Li S, Chen L, Jin W, Ma X, Ma Y, Dong F, et al. Influence of body mass index on incidence and prognosis of acute myeloid leukemia and acute promyelocytic leukemia: a meta-analysis. Sci Rep. 2017;7:17998. 29269861 10.1038/s41598-017-18278-x PMC5740068

[55] Shallis RM, Weiss JJ, Deziel NC, Gore SD. A clandestine culprit with critical consequences: benzene and acute myeloid leukemia. Blood Rev. 2021;47:100736. 32771228 10.1016/j.blre.2020.100736

[56] Linet MS, Gilbert ES, Vermeulen R, Dores GM, Yin SN, Portengen L, et al. Benzene exposure response and risk of myeloid neoplasms in Chinese workers: a multicenter case-cohort study. J Natl Cancer Inst. 2019;111:465–74. 30520970 10.1093/jnci/djy143 PMC6681472

[57] Checkoway H, Dell LD, Boffetta P, Gallagher AE, Crawford L, Lees PS, Mundt KA. Formaldehyde exposure and mortality risks from acute myeloid leukemia and other lymphohematopoietic malignancies in the US National Cancer Institute cohort study of workers in formaldehyde industries. J Occup Environ Med. 2015;57:785–94. 26147546 10.1097/JOM.0000000000000466 PMC4479664

[58] Adamović D, Čepić Z, Adamović S, Stošić M, Obrovski B, Morača S, Vojinović Miloradov M. Occupational exposure to formaldehyde and cancer risk assessment in an anatomy laboratory. Int J Environ Res Public Health. 2021;18. 10.3390/ijerph182111198 PMC8583012 34769715

[59] Bi X, French Z, Palmisiano N, Wen KY, Wilde L. The prognostic impact of cigarette smoking on survival in acute myeloid leukemia with TP53 mutations and/or 17p deletions. Ann Hematol. 2022;101:1251–9. 35288759 10.1007/s00277-022-04812-z

[60] Shi H, Shao X, Hong Y. Association between cigarette smoking and the susceptibility of acute myeloid leukemia: a systematic review and meta-analysis. Eur Rev Med Pharmacol Sci. 2019;23:10049–57. 31799675 10.26355/eurrev_201911_19572

[61] Fircanis S, Merriam P, Khan N, Castillo JJ. The relation between cigarette smoking and risk of acute myeloid leukemia: an updated meta-analysis of epidemiological studies. Am J Hematol. 2014;89:E125–32. 24753145 10.1002/ajh.23744

[62] Ugai T, Matsuo K, Oze I, Ito H, Wakai K, Wada K, et al. Smoking and subsequent risk of acute myeloid leukaemia: a pooled analysis of 9 cohort studies in Japan. Hematol Oncol. 2018;36:262–8. 28681440 10.1002/hon.2457

[63] Global age-sex. -specific mortality, life expectancy, and population estimates in 204 countries and territories and 811 subnational locations, 1950–2021, and the impact of the COVID-19 pandemic: a comprehensive demographic analysis for the global burden of Disease Study 2021. Lancet. 2024;403:1989–2056. 38484753 10.1016/S0140-6736(24)00476-8 PMC11126395

