# Supplementary material for: Global, regional, and national burden of acute myeloid leukemia, 1990–2021: a systematic analysis for the global burden of disease study 2021
Source: Biomark Res. 2024 Sep 11;12:101. doi: 10.1186/s40364-024-00649-y (PMC11389310; doi:10.1186/s40364-024-00649-y)
Supplement: Supplementary file 1 — Supplementary Material 1 [file 40364_2024_649_MOESM1_ESM.docx]

Table S1 The incidence cases and ASIR of AML in 1990 and 2021, and its temporal trends from 1990 to 2021 in 204 countries or territories

| location | | Num_1990 | | ASIR_1990 | Num_2021 | ASIR_2021 | Num_change | | EAPC_CI | |
| --- | --- | --- | --- | --- | --- | --- | --- | --- | --- | --- |
| Afghanistan | | 277.6 (114.3-572.1) | | 3.6 (1.5-7) | 610.9 (297.5-1121) | 3.9 (1.8-6.9) | 1.2% (0.6-2.2) | | 0.31 (0.25-0.38) | |
| Albania | | 39 (27.8-59.1) | | 1.6 (1.1-2.4) | 55.3 (34.6-81.7) | 1.4 (0.9-2.1) | 0.42% (-0.09-1.09) | | -0.06 (-0.22-0.1) | |
| Algeria | | 208.3 (129.5-290.7) | | 1 (0.6-1.4) | 339.5 (224.1-537.3) | 0.9 (0.6-1.4) | 0.63% (0.12-1.49) | | -0.28 (-0.35 to -0.21) | |
| American Samoa | | 0.8 (0.5-1) | | 2.3 (1.2-3) | 0.6 (0.4-1) | 1.2 (0.9-2) | -0.26% (-0.52-0.55) | | -2.84 (-3.19 to -2.48) | |
| Andorra | | 2 (1.3-3.1) | | 3.5 (2.2-5.5) | 3.7 (2.2-5.4) | 2.6 (1.5-3.8) | 0.84% (0.09-2.09) | | -0.67 (-0.85 to -0.49) | |
| Angola | | 50.5 (22.6-106.7) | | 0.7 (0.4-1.1) | 136.8 (73.7-202) | 0.7 (0.4-1) | 1.71% (0.26-3.82) | | -0.16 (-0.23 to -0.09) | |
| Antigua and Barbuda | | 0.8 (0.7-0.9) | | 1.4 (1.3-1.6) | 1.9 (1.8-2) | 1.9 (1.8-2) | 1.34% (1.03-1.65) | | 1.17 (0.93-1.4) | |
| Argentina | | 629.5 (574.8-693.5) | | 1.9 (1.8-2.1) | 1027.6 (940.1-1135.9) | 1.9 (1.8-2.1) | 0.63% (0.44-0.85) | | 0.3 (0.02-0.57) | |
| Armenia | | 30.3 (24-37.4) | | 0.9 (0.8-1.2) | 53.1 (44.2-64.3) | 1.4 (1.1-1.6) | 0.75% (0.29-1.43) | | 2.05 (1.33-2.78) | |
| Australia | | 616.8 (571.1-666.1) | | 3.2 (3-3.5) | 2145.2 (1901.3-2393) | 4.9 (4.4-5.5) | 2.48% (2.07-2.89) | | 1.14 (0.82-1.47) | |
| Austria | | 216.1 (199.6-231.3) | | 2 (1.9-2.2) | 437.7 (386.5-484.3) | 2.5 (2.3-2.8) | 1.03% (0.82-1.26) | | 1.19 (0.93-1.45) | |
| Azerbaijan | | 107.3 (74.3-164.9) | | 1.6 (1.1-2.4) | 145.6 (97.5-232.5) | 1.4 (0.9-2.2) | 0.36% (-0.08-1) | | -0.5 (-0.64 to -0.36) | |
| Bahrain | | 7 (4.6-9.3) | | 2.8 (1.7-3.7) | 20.7 (13.4-32.5) | 2 (1.3-2.9) | 1.96% (1-3.36) | | -1.49 (-1.69 to -1.29) | |
| Bangladesh | | 868.1 (455.9-1726.2) | | 1 (0.6-1.6) | 1308.1 (823-1932) | 0.9 (0.5-1.3) | 0.51% (-0.39-1.71) | | -0.39 (-0.46 to -0.33) | |
| Barbados | | 4.9 (4.5-5.4) | | 1.9 (1.7-2) | 10 (7.7-12.4) | 2.4 (1.9-3) | 1.02% (0.54-1.52) | | 1.42 (1.23-1.62) | |
| Belarus | | 143.8 (119.1-182.1) | | 1.2 (1-1.5) | 278.9 (224.6-339.1) | 2.1 (1.7-2.5) | 0.94% (0.45-1.55) | | 1.53 (1-2.06) | |
| Belgium | | 342.9 (309.9-374.8) | | 2.4 (2.2-2.6) | 699.9 (605.6-787.5) | 3.2 (2.8-3.5) | 1.04% (0.78-1.36) | | 1 (0.71-1.29) | |
| Belize | | 0.5 (0.5-0.6) | | 0.3 (0.3-0.4) | 1.9 (1.6-2.1) | 0.5 (0.4-0.6) | 2.54% (1.85-3.32) | | 1.72 (1.33-2.11) | |
| Benin | | 11.9 (5.9-25.2) | | 0.3 (0.2-0.5) | 33.2 (16-45.8) | 0.3 (0.2-0.5) | 1.79% (0.33-3.68) | | 0.65 (0.55-0.74) | |
| Bermuda | | 1.7 (1.5-1.9) | | 2.8 (2.5-3.2) | 2.3 (1.9-2.7) | 2.2 (1.8-2.6) | 0.33% (0.07-0.63) | | -0.58 (-0.75 to -0.41) | |
| Bhutan | | 4.2 (1.9-7.8) | | 0.9 (0.5-1.6) | 6.4 (3.9-10.7) | 1 (0.6-1.6) | 0.51% (-0.3-1.84) | | 0.05 (-0.03-0.13) | |
| Bosnia and Herzegovina | | 62.6 (47.5-89.1) | | 1.5 (1.1-2.1) | 91.9 (59.1-127) | 1.6 (1-2.2) | 0.47% (0.02-1.17) | | 0.61 (0.47-0.76) | |
| Botswana | | 8.9 (5.4-14.1) | | 1.2 (0.8-1.8) | 20.3 (11.8-30.1) | 1.1 (0.7-1.7) | 1.28% (0.54-2.45) | | -0.19 (-0.32 to -0.06) | |
| Brazil | | 2102.7 (2032.1-2178.5) | | 1.8 (1.7-1.8) | 4529.7 (4242.7-4752.5) | 1.9 (1.7-2) | 1.15% (1.04-1.26) | | 0.28 (0.15-0.4) | |
| Bulgaria | | 162.7 (149.9-177.2) | | 1.5 (1.4-1.7) | 312.6 (251-382.6) | 2.6 (2.1-3.2) | 0.92% (0.52-1.44) | | 2.36 (2.02-2.7) | |
| Burkina Faso | | 22.8 (10.8-45.7) | | 0.3 (0.2-0.4) | 61.6 (27-91.6) | 0.4 (0.2-0.5) | 1.7% (0.46-3.38) | | 0.98 (0.88-1.09) | |
| Burundi | | 24.9 (10-45.5) | | 0.5 (0.2-0.9) | 43.4 (18.1-69.8) | 0.5 (0.2-0.8) | 0.75% (-0.18-1.99) | | -0.42 (-0.5 to -0.33) | |
| Cambodia | | 172.4 (86.1-340.8) | | 2.5 (1.3-4.2) | 351.3 (210.8-493.3) | 2.5 (1.5-3.4) | 1.04% (0.14-2.33) | | -0.04 (-0.08-0) | |
| Cameroon | | 26.5 (14.2-50) | | 0.3 (0.2-0.5) | 82.6 (43-119.6) | 0.4 (0.2-0.5) | 2.12% (0.66-3.97) | | 0.66 (0.56-0.76) | |
| Canada | | 838.4 (787-891.4) | | 2.7 (2.5-2.8) | 2142.3 (1923.8-2325.8) | 3.1 (2.8-3.4) | 1.56% (1.32-1.79) | | 1.06 (0.86-1.26) | |
| Central African Republic | | 14.3 (6.7-29.6) | | 0.8 (0.4-1.3) | 25.1 (13.1-40.6) | 0.7 (0.4-1.1) | 0.76% (0.22-1.56) | | -0.36 (-0.4 to -0.32) | |
| Chad | | 11.7 (5.7-25.3) | | 0.2 (0.1-0.4) | 40.6 (17.3-67.8) | 0.3 (0.2-0.5) | 2.47% (1.11-4.36) | | 1.45 (1.32-1.58) | |
| Chile | | 171.8 (160.8-182.4) | | 1.5 (1.4-1.6) | 391.9 (355.9-428.3) | 1.7 (1.5-1.8) | 1.28% (1.06-1.53) | | 0.68 (0.49-0.87) | |
| China | | 15309.3 (8205.9-24141.8) | | 1.5 (0.8-2.2) | 17835.2 (11876.2-24800.4) | 1 (0.7-1.4) | 0.16% (-0.31-1.01) | | -1.51 (-1.7 to -1.32) | |
| Colombia | | 336.9 (314.7-359.5) | | 1.3 (1.2-1.4) | 877.2 (727.5-1036) | 1.7 (1.4-2) | 1.6% (1.15-2.1) | | 0.66 (0.49-0.84) | |
| Comoros | | 2 (0.8-2.9) | | 0.5 (0.3-0.8) | 3.4 (1.9-5.1) | 0.5 (0.3-0.8) | 0.74% (0.02-1.76) | | -0.16 (-0.36-0.04) | |
| Congo | | 13.3 (8-22.3) | | 0.8 (0.5-1.3) | 28.3 (16.8-40.1) | 0.7 (0.4-1) | 1.13% (0.25-2.4) | | -0.55 (-0.66 to -0.45) | |
| Cook Islands | | 0.1 (0.1-0.2) | | 0.9 (0.5-1.2) | 0.2 (0.1-0.2) | 0.7 (0.4-1) | 0.19% (-0.12-0.65) | | -0.76 (-0.86 to -0.67) | |
| Costa Rica | | 39.3 (36.5-43.9) | | 1.7 (1.6-1.9) | 128.6 (113.5-144.3) | 2.4 (2.2-2.7) | 2.27% (1.82-2.72) | | 1.11 (0.88-1.34) | |
| Croatia | | 98 (82.2-115.7) | | 1.7 (1.4-2) | 208.1 (166.9-254.2) | 2.6 (2.1-3.1) | 1.12% (0.64-1.74) | | 1.77 (1.6-1.94) | |
| Cuba | | 178.4 (166.3-193.9) | | 1.7 (1.6-1.8) | 273.5 (234.8-316.6) | 1.7 (1.5-2) | 0.53% (0.28-0.82) | | 0.17 (-0.03-0.37) | |
| Cyprus | | 24.8 (18.6-37.4) | | 3.2 (2.4-5.1) | 57.4 (35.4-74.1) | 2.8 (1.8-3.6) | 1.31% (0.35-2.33) | | 0.07 (-0.11-0.25) | |
| Democratic Republic of the Congo | | 164.3 (80.8-277) | | 0.7 (0.3-1) | 334.6 (188.3-472.2) | 0.6 (0.3-0.9) | 1.04% (0.18-2.2) | | -0.22 (-0.36 to -0.09) | |
| Denmark | | 240.7 (225.7-256.5) | | 3.3 (3.1-3.5) | 303.5 (267.9-340.3) | 2.7 (2.4-3) | 0.26% (0.11-0.44) | | -0.51 (-0.73 to -0.29) | |
| Djibouti | | 1.5 (0.7-2.3) | | 0.5 (0.2-0.8) | 5 (2.5-8.3) | 0.5 (0.3-0.9) | 2.27% (1.11-3.91) | | 0.21 (0.07-0.35) | |
| Dominica | | 1.1 (0.8-1.4) | | 1.7 (1.3-2.3) | 1.3 (1-1.9) | 1.8 (1.3-2.5) | 0.26% (-0.08-0.7) | | 0.22 (0.15-0.28) | |
| Dominican Republic | | 75.6 (53.1-110) | | 1.2 (0.8-1.8) | 130.5 (92.5-197.3) | 1.2 (0.9-1.9) | 0.73% (0.22-1.41) | | 0.6 (0.46-0.73) | |
| Ecuador | | 97.9 (90.1-108.1) | | 1.3 (1.2-1.4) | 310.5 (245.9-384.5) | 1.8 (1.4-2.3) | 2.17% (1.48-3.02) | | 1.88 (1.42-2.35) | |
| Egypt | | 730.6 (463.6-1401.7) | | 1.9 (1.2-3.7) | 2200.6 (1481.4-3159.5) | 2.9 (1.9-4.5) | 2.01% (0.77-3.65) | | 2.08 (1.78-2.39) | |
| El Salvador | | 57.8 (38.7-91.1) | | 1.3 (0.9-1.9) | 97.3 (59.5-128) | 1.5 (0.9-2) | 0.68% (0.16-1.33) | | 0.74 (0.64-0.84) | |
| Equatorial Guinea | | 2.1 (1-3.9) | | 0.7 (0.4-1.1) | 6.4 (3.3-10.3) | 0.7 (0.4-1.1) | 2.01% (0.38-4.33) | | -0.11 (-0.2 to -0.01) | |
| Eritrea | | 11.9 (5.3-21.4) | | 0.5 (0.2-0.8) | 26.4 (14.2-37.8) | 0.6 (0.3-0.8) | 1.21% (0.12-2.76) | | 0.36 (0.28-0.45) | |
| Estonia | | 27.2 (24-30.8) | | 1.6 (1.4-1.8) | 46.7 (39.2-55) | 2.2 (1.9-2.5) | 0.71% (0.43-1.07) | | 1 (0.52-1.48) | |
| Ethiopia | | 458 (185.3-930.1) | | 1.2 (0.5-2.2) | 738.7 (396-1164.5) | 1 (0.5-1.6) | 0.61% (-0.39-2.02) | | -0.92 (-1.06 to -0.78) | |
| Fiji | | 23.3 (14.2-30.5) | | 4.3 (2.6-5.7) | 33.6 (18.6-46) | 4.1 (2.3-5.7) | 0.44% (0.07-1.01) | | -0.08 (-0.25-0.1) | |
| Finland | | 132.7 (121.9-144.2) | | 2 (1.9-2.2) | 247.8 (215.8-280.3) | 2.2 (2-2.5) | 0.87% (0.61-1.15) | | 0.37 (0.22-0.51) | |
| France | | 1678.8 (1573.6-1795.4) | | 2.2 (2.1-2.3) | 3782.5 (3220-4247.7) | 2.8 (2.5-3.1) | 1.25% (0.96-1.56) | | 1.21 (1.01-1.41) | |
| Gabon | | 5.9 (3.3-8.3) | | 0.8 (0.5-1.2) | 10.4 (6.1-15.3) | 0.8 (0.5-1.1) | 0.75% (0.16-1.5) | | -0.28 (-0.37 to -0.19) | |
| Georgia | | 75 (61.7-94.8) | | 1.3 (1.1-1.7) | 95.3 (82.9-109.7) | 2 (1.7-2.3) | 0.27% (-0.04-0.62) | | 2.31 (1.57-3.07) | |
| Germany | | 2412.2 (2180-2634.8) | | 2.1 (2-2.3) | 5351.9 (4793.1-5888.1) | 2.9 (2.6-3.1) | 1.22% (0.95-1.52) | | 1.12 (1.02-1.22) | |
| Ghana | | 70.5 (33-103) | | 0.6 (0.3-0.8) | 96.8 (62.5-168.2) | 0.4 (0.2-0.7) | 0.37% (-0.27-2.3) | | -2.16 (-2.6 to -1.71) | |
| Greece | | 332 (313.1-353.4) | | 2.3 (2.2-2.5) | 778 (699.6-838.7) | 3.5 (3.2-3.7) | 1.34% (1.15-1.53) | | 1.21 (1.06-1.36) | |
| Greenland | | 0.7 (0.5-0.8) | | 1.8 (1.2-2.2) | 0.8 (0.5-1.1) | 1.3 (0.7-1.8) | 0.16% (-0.13-0.65) | | -1.12 (-1.19 to -1.05) | |
| Grenada | | 0.7 (0.6-0.9) | | 0.9 (0.7-1.1) | 1.3 (1.1-1.4) | 1.2 (1-1.3) | 0.76% (0.35-1.32) | | 1.08 (0.94-1.23) | |
| Guam | | 1.7 (1.3-2.3) | | 1.7 (1.3-2.3) | 3.2 (2.1-4) | 1.8 (1.2-2.2) | 0.89% (0.41-1.54) | | 0.33 (-0.14-0.8) | |
| Guatemala | | 66.1 (53.2-77.3) | | 0.9 (0.7-1) | 148.1 (126.6-171.8) | 1.1 (0.9-1.3) | 1.24% (0.76-1.96) | | 0.72 (0.48-0.96) | |
| Guinea | | 6.8 (3-14.8) | | 0.1 (0.1-0.2) | 10.7 (5.5-17.1) | 0.1 (0.1-0.2) | 0.57% (-0.15-1.58) | | -0.24 (-0.28 to -0.2) | |
| Guinea-Bissau | | 2.7 (1.3-6.1) | | 0.4 (0.2-0.6) | 5.3 (2.9-7.4) | 0.4 (0.2-0.6) | 0.97% (0.02-2.35) | | 0.77 (0.65-0.89) | |
| Guyana | | 1.6 (1.3-1.9) | | 0.3 (0.2-0.3) | 2.9 (2.1-3.8) | 0.4 (0.3-0.5) | 0.8% (0.23-1.58) | | 1.91 (1.66-2.16) | |
| Haiti | | 86.5 (43-185.3) | | 1.6 (0.9-3) | 157.1 (87.7-256.4) | 1.5 (0.9-2.3) | 0.82% (0.13-1.87) | | -0.05 (-0.11-0.02) | |
| Honduras | | 53.2 (29.8-83.7) | | 1.4 (0.9-2.1) | 116 (72.2-175.8) | 1.5 (0.9-2.3) | 1.18% (0.49-2.31) | | 0.14 (0.08-0.2) | |
| Hungary | | 290.6 (271.3-310.9) | | 2.1 (2-2.3) | 433.7 (365.7-518.3) | 2.5 (2.1-3) | 0.49% (0.23-0.8) | | 0.69 (0.54-0.84) | |
| Iceland | | 6.2 (5.5-6.7) | | 2.2 (2-2.4) | 14.9 (12.9-17) | 2.7 (2.4-3.1) | 1.41% (1.05-1.85) | | 0.73 (0.64-0.83) | |
| India | | 5289.5 (3302.3-8511.9) | | 0.8 (0.5-1.2) | 11040.3 (8211.1-15171) | 0.9 (0.6-1.2) | 1.09% (0.27-2.02) | | 0.17 (0.07-0.27) | |
| Indonesia | | 2773.2 (1801.3-4215.5) | | 2 (1.3-2.9) | 5554.8 (3964.3-7575) | 2.2 (1.6-3) | 1% (0.45-1.73) | | 0.22 (0.12-0.33) | |
| Iraq | | 224.6 (119-515.8) | | 1.8 (0.9-3.8) | 558.3 (353.6-1034.7) | 1.8 (1.1-3.4) | 1.49% (0.6-3.22) | | 0.04 (-0.05-0.13) | |
| Ireland | | 81.7 (77-86.8) | | 2 (1.9-2.1) | 163.8 (147-181.8) | 2.2 (2-2.4) | 1.01% (0.81-1.26) | | 0.51 (0.21-0.81) | |
| Israel | | 136.5 (124.2-149.2) | | 2.8 (2.5-3.1) | 358.5 (316.7-396) | 3 (2.6-3.3) | 1.63% (1.32-1.97) | | 0.49 (0.25-0.73) | |
| Italy | | 1588.9 (1501.6-1655.7) | | 2 (1.9-2.1) | 3947 (3493.9-4267) | 2.9 (2.7-3.1) | 1.48% (1.27-1.68) | | 1.28 (1.03-1.53) | |
| Jamaica | | 12.9 (11.1-15.3) | | 0.6 (0.5-0.7) | 32.2 (24.5-42.2) | 1.1 (0.8-1.4) | 1.48% (0.75-2.4) | | 2.39 (2.02-2.76) | |
| Japan | | 3503.1 (3347-3618.3) | | 2.3 (2.2-2.3) | 6506.4 (5635-7006.6) | 2 (1.8-2.1) | 0.86% (0.66-0.99) | | -0.06 (-0.3-0.19) | |
| Jordan | | 110.3 (82.6-144.8) | | 5 (3.7-6.5) | 338.3 (235.6-447.2) | 3.7 (2.6-4.9) | 2.07% (1.21-3.14) | | -1.14 (-1.34 to -0.93) | |
| Kazakhstan | | 234.2 (194.6-286.6) | | 1.5 (1.3-1.9) | 227.7 (186.9-272.7) | 1.2 (1-1.4) | -0.03% (-0.23-0.23) | | -0.56 (-0.88 to -0.25) | |
| Kenya | | 57.8 (33.3-86) | | 0.4 (0.2-0.6) | 160.1 (106.6-221.8) | 0.5 (0.3-0.7) | 1.77% (0.69-3.02) | | 1.08 (0.95-1.21) | |
| Kiribati | | 0.8 (0.3-1.4) | | 1.3 (0.5-2.2) | 1.5 (0.6-2.4) | 1.4 (0.6-2.3) | 0.75% (0.24-1.35) | | 0.15 (0.08-0.22) | |
| Kuwait | | 14.5 (13-16.3) | | 1.6 (1.4-1.8) | 47 (37.8-56.2) | 1.3 (1-1.6) | 2.24% (1.58-2.95) | | -0.34 (-0.89-0.22) | |
| Kyrgyzstan | | 33.9 (28-41.9) | | 0.8 (0.7-1) | 65.8 (54-79.1) | 1.1 (0.9-1.3) | 0.94% (0.44-1.53) | | 1.64 (1.31-1.96) | |
| Latvia | | 70.4 (61.7-80.7) | | 2.3 (2.1-2.6) | 57 (47.6-67) | 1.9 (1.6-2.2) | -0.19% (-0.33 to -0.01) | | -0.6 (-0.98 to -0.21) | |
| Lebanon | | 66.4 (44.3-112.3) | | 2.8 (1.9-4.6) | 156.8 (114.8-211) | 2.6 (1.9-3.5) | 1.36% (0.51-2.56) | | 0.07 (-0.05-0.19) | |
| Lesotho | | 9.6 (6.1-15.7) | | 1 (0.6-1.5) | 20.2 (12.8-37.3) | 1.5 (1-2.7) | 1.11% (0.32-2.3) | | 2.03 (1.76-2.3) | |
| Liberia | | 6.1 (2.7-14.7) | | 0.3 (0.2-0.5) | 12.3 (5.7-17.7) | 0.3 (0.2-0.5) | 1.02% (-0.1-2.85) | | 0.9 (0.68-1.13) | |
| Libya | | 76.9 (52.7-118.5) | | 3 (2-4.7) | 172.6 (103.6-293.5) | 2.9 (1.8-4.8) | 1.24% (0.33-2.47) | | 0.18 (0.05-0.31) | |
| Lithuania | | 55.7 (49.1-63) | | 1.4 (1.2-1.6) | 112.5 (94.9-130.1) | 2.4 (2.1-2.8) | 1.02% (0.69-1.42) | | 2.18 (1.81-2.55) | |
| Luxembourg | | 15.9 (14.9-16.9) | | 3.2 (3-3.4) | 37.1 (33.3-41.2) | 3.6 (3.3-4) | 1.33% (1.08-1.63) | | 0.57 (0.36-0.78) | |
| Madagascar | | 44.5 (21-66.4) | | 0.5 (0.2-0.7) | 86.5 (42.9-123.2) | 0.4 (0.2-0.6) | 0.94% (0.13-1.86) | | -0.27 (-0.38 to -0.16) | |
| Malawi | | 17.2 (5.4-31.9) | | 0.2 (0.1-0.3) | 22.6 (10.7-39) | 0.2 (0.1-0.2) | 0.32% (-0.3-1.72) | | -0.61 (-0.7 to -0.53) | |
| Malaysia | | 240.6 (148-312.9) | | 1.8 (1.2-2.4) | 555.6 (393.7-759.5) | 1.8 (1.3-2.6) | 1.31% (0.77-2.18) | | -0.02 (-0.14-0.11) | |
| Maldives | | 2.6 (1.5-5.3) | | 2 (1.2-3.4) | 5.2 (3.3-7.6) | 1.3 (0.9-1.8) | 0.97% (-0.13-2.51) | | -1.54 (-1.6 to -1.48) | |
| Mali | | 20 (7.9-40.8) | | 0.3 (0.1-0.4) | 36.9 (20.2-56.9) | 0.2 (0.1-0.3) | 0.84% (0.12-2.12) | | -0.52 (-0.58 to -0.46) | |
| Malta | | 8.8 (7.9-9.7) | | 2.1 (1.9-2.3) | 23.9 (20.8-27) | 2.7 (2.4-3.1) | 1.73% (1.34-2.18) | | 0.93 (0.7-1.16) | |
| Marshall Islands | | 0.6 (0.3-0.8) | | 1.8 (0.9-2.6) | 0.9 (0.5-1.3) | 2 (1-2.9) | 0.61% (0.16-1.16) | | 0.34 (0.27-0.4) | |
| Mauritania | | 4.5 (2.7-8.4) | | 0.3 (0.2-0.5) | 10.2 (5.7-14.1) | 0.3 (0.2-0.5) | 1.24% (0.25-2.44) | | 0.52 (0.42-0.61) | |
| Mauritius | | 6.2 (5.7-6.9) | | 0.7 (0.6-0.8) | 15.3 (13.9-16.4) | 0.9 (0.9-1) | 1.45% (1.12-1.77) | | 5.12 (1.91-8.43) | |
| Mexico | | 969.1 (939.1-1007.1) | | 1.4 (1.3-1.4) | 1988.3 (1753-2204.6) | 1.5 (1.4-1.7) | 1.05% (0.82-1.28) | | 0.19 (0.08-0.29) | |
| Mongolia | | 22.2 (13.4-36.9) | | 1.3 (0.8-2) | 38.5 (26.4-51.3) | 1.3 (0.9-1.7) | 0.73% (0.02-1.95) | | 0 (-0.11-0.11) | |
| Montenegro | | 12 (9-16.5) | | 1.9 (1.4-2.6) | 18.1 (12.7-25.3) | 2 (1.4-2.8) | 0.51% (0.1-1.11) | | 0.32 (0.22-0.42) | |
| Morocco | | 90 (59.7-145.8) | | 0.5 (0.3-0.8) | 180.2 (118.7-288.7) | 0.5 (0.3-0.8) | 1% (0.41-1.79) | | 0.23 (0.18-0.28) | |
| Mozambique | | 99.5 (34.1-190.1) | | 0.8 (0.3-1.3) | 171.6 (86.8-295.1) | 0.7 (0.4-1.1) | 0.72% (-0.13-2.64) | | 0.09 (0.01-0.18) | |
| Myanmar | | 810.7 (414.2-1635.3) | | 2.6 (1.4-4.8) | 1122.9 (796.7-1609.5) | 2.2 (1.6-3.1) | 0.39% (-0.22-1.44) | | -0.73 (-0.84 to -0.63) | |
| Namibia | | 7.8 (5-11.6) | | 0.9 (0.6-1.3) | 16.2 (10.4-24.9) | 0.9 (0.6-1.5) | 1.07% (0.45-2.14) | | 0.03 (-0.14-0.21) | |
| Nepal | | 134.8 (66.4-265.8) | | 0.9 (0.5-1.5) | 241.4 (165.2-370.4) | 0.9 (0.6-1.4) | 0.79% (0.01-2.09) | | 0.31 (0.15-0.46) | |
| Netherlands | | 468.3 (435.7-500.7) | | 2.5 (2.3-2.7) | 937.4 (826.8-1048.5) | 2.8 (2.5-3.1) | 1% (0.81-1.24) | | 0.55 (0.37-0.74) | |
| New Zealand | | 112.8 (102.4-121.4) | | 2.9 (2.7-3.2) | 188.3 (165.8-208.4) | 2.4 (2.2-2.7) | 0.67% (0.47-0.88) | | -0.57 (-0.93 to -0.21) | |
| Nicaragua | | 30 (18.8-46.4) | | 0.9 (0.6-1.3) | 53.4 (34.8-70.8) | 0.9 (0.6-1.2) | 0.78% (0.11-1.44) | | 0.27 (0.12-0.43) | |
| Niger | | 19.1 (7.3-49.4) | | 0.3 (0.1-0.5) | 48.9 (19.9-75.6) | 0.3 (0.1-0.5) | 1.57% (0.06-3.98) | | 0.48 (0.4-0.56) | |
| Nigeria | | 211.7 (106.2-423.3) | | 0.3 (0.2-0.5) | 456.1 (254.7-620.8) | 0.3 (0.2-0.4) | 1.15% (0.23-2.23) | | 0.07 (-0.01-0.14) | |
| Northern Mariana Islands | | 0.6 (0.3-0.9) | | 2.1 (1.1-2.9) | 0.4 (0.3-1) | 0.9 (0.6-2) | -0.32% (-0.66-0.6) | | -3.23 (-3.53 to -2.93) | |
| Norway | | 159.1 (150.4-166.9) | | 2.6 (2.5-2.7) | 241.2 (216.4-261.6) | 2.5 (2.2-2.7) | 0.52% (0.39-0.65) | | 0 (-0.13-0.14) | |
| Oman | | 24.7 (15-34.8) | | 1.9 (1-2.8) | 45.6 (30.5-61.3) | 1.5 (1-2) | 0.85% (0.23-1.82) | | -0.45 (-0.62 to -0.27) | |
| Pakistan | | 888.9 (551.7-1473.6) | | 1 (0.6-1.6) | 2069.4 (1416-3272.1) | 1.1 (0.8-1.8) | 1.33% (0.63-2.55) | | 0.3 (0.18-0.42) | |
| Palestine | | 12.1 (7.2-20) | | 0.9 (0.5-1.4) | 26.9 (17.1-40) | 0.8 (0.5-1.1) | 1.22% (0.42-2.38) | | -0.62 (-0.7 to -0.54) | |
| Panama | | 26.6 (24.3-29.3) | | 1.3 (1.2-1.4) | 80.7 (65.2-97.4) | 1.9 (1.5-2.2) | 2.03% (1.39-2.68) | | 1.16 (0.99-1.33) | |
| Papua New Guinea | | 49.7 (19.3-76.4) | | 1.6 (0.6-2.5) | 126.8 (61.3-194.2) | 1.6 (0.7-2.5) | 1.55% (0.83-2.52) | | -0.06 (-0.1 to -0.01) | |
| Paraguay | | 37.4 (26.6-52) | | 1.2 (0.9-1.7) | 100.9 (66.6-136.5) | 1.6 (1.1-2.2) | 1.7% (0.92-2.73) | | 1.12 (0.93-1.3) | |
| Peru | | 255.7 (170.1-386.6) | | 1.5 (1-2.1) | 549 (309-750.9) | 1.6 (0.9-2.2) | 1.15% (0.24-2.18) | | 0.48 (0.34-0.62) | |
| Philippines | | 1037.8 (758.8-1450) | | 2.3 (1.8-3) | 2060.9 (1643.8-2651.3) | 2.1 (1.7-2.9) | 0.99% (0.53-1.47) | | -0.25 (-0.33 to -0.18) | |
| Poland | | 780.8 (707.4-907) | | 1.9 (1.7-2.1) | 1361.9 (1230.2-1482.5) | 2.1 (1.9-2.2) | 0.74% (0.5-1.02) | | 0.74 (0.43-1.04) | |
| Portugal | | 245 (227.4-262.6) | | 2 (1.9-2.1) | 553.7 (483.4-618.5) | 2.5 (2.2-2.7) | 1.26% (0.98-1.54) | | 0.75 (0.56-0.94) | |
| Puerto Rico | | 86.5 (80.4-93.8) | | 2.4 (2.2-2.6) | 146.3 (119.3-173.7) | 2.6 (2.1-3.1) | 0.69% (0.37-1.06) | | 0.11 (-0.08-0.3) | |
| Qatar | | 4.6 (2.8-6.3) | | 2.3 (1.3-3.2) | 21.2 (13.9-33.3) | 1.4 (0.9-2.1) | 3.57% (1.99-6.17) | | -1.63 (-1.93 to -1.33) | |
| Romania | | 302.3 (274.1-334.1) | | 1.2 (1.1-1.3) | 576.2 (493.3-654.6) | 1.8 (1.6-2.1) | 0.91% (0.6-1.25) | | 1.62 (1.48-1.77) | |
| Russian Federation | | 2233.1 (2020.8-2440.4) | | 1.4 (1.2-1.5) | 2942.9 (2699.6-3189.6) | 1.4 (1.3-1.5) | 0.32% (0.16-0.48) | | 0.26 (0.04-0.48) | |
| Rwanda | | 37.3 (16.2-70.1) | | 0.6 (0.3-1.1) | 52.8 (26.6-80.3) | 0.5 (0.3-0.8) | 0.42% (-0.38-1.67) | | -1.03 (-1.18 to -0.88) | |
| Saint Kitts and Nevis | | 0.1 (0.1-0.1) | | 0.2 (0.2-0.3) | 0.2 (0.1-0.2) | 0.3 (0.2-0.3) | 0.98% (0.54-1.51) | | 1.12 (0.9-1.34) | |
| Saint Lucia | | 1.3 (1.2-1.5) | | 1.2 (1.1-1.4) | 2.6 (2.1-3.2) | 1.3 (1-1.5) | 0.93% (0.46-1.4) | | 0.08 (-0.1-0.27) | |
| Saint Vincent and the Grenadines | | 1.1 (1-1.2) | | 1 (0.9-1.1) | 1.5 (1.2-1.7) | 1.2 (1-1.4) | 0.35% (0.13-0.6) | | 0.78 (0.61-0.95) | |
| Samoa | | 3.1 (1.8-4.3) | | 2.5 (1.4-3.5) | 4 (2.5-5.6) | 2.4 (1.4-3.3) | 0.31% (0.01-0.71) | | -0.24 (-0.33 to -0.15) | |
| Sao Tome and Principe | | 0.3 (0.1-0.4) | | 0.2 (0.2-0.3) | 0.3 (0.2-0.6) | 0.2 (0.1-0.4) | 0.33% (-0.19-1.33) | | -0.27 (-0.4 to -0.15) | |
| Saudi Arabia | | 83.2 (52.5-141.7) | | 0.8 (0.6-1.5) | 359.2 (240.1-630.3) | 1.2 (0.8-2) | 3.32% (1.88-6.21) | | 1.49 (1.1-1.89) | |
| Senegal | | 18.2 (9.5-34.1) | | 0.3 (0.2-0.5) | 40.2 (22-56.2) | 0.4 (0.2-0.5) | 1.21% (0.09-2.76) | | 0.95 (0.79-1.11) | |
| Serbia | | 215.3 (155.5-287.7) | | 2.1 (1.5-2.8) | 315.8 (194.1-407.3) | 2.1 (1.3-2.7) | 0.47% (0.1-0.9) | | 0.21 (0.12-0.3) | |
| Seychelles | | 1.1 (0.7-1.4) | | 1.7 (1.1-2.2) | 1.5 (0.9-2) | 1.4 (0.8-1.8) | 0.4% (0.09-0.82) | | -0.37 (-0.48 to -0.25) | |
| Sierra Leone | | 10 (4.6-23) | | 0.3 (0.1-0.5) | 21.5 (10.1-30.6) | 0.3 (0.2-0.4) | 1.15% (0.03-2.85) | | 1.1 (0.95-1.25) | |
| Singapore | | 60.8 (56.6-64.8) | | 2.4 (2.2-2.6) | 156.7 (143-170.3) | 2 (1.9-2.2) | 1.58% (1.32-1.86) | | -0.21 (-0.49-0.08) | |
| Slovakia | | 133.8 (105.2-196.2) | | 2.3 (1.8-3.4) | 178.7 (105.2-238) | 2.1 (1.3-2.8) | 0.34% (-0.18-0.96) | | -0.05 (-0.14-0.03) | |
| Slovenia | | 31.6 (28.1-35.4) | | 1.3 (1.2-1.5) | 70.6 (57.4-84) | 1.7 (1.4-2.1) | 1.23% (0.8-1.74) | | 1.41 (1.14-1.67) | |
| Solomon Islands | | 4.3 (1.8-6.5) | | 1.8 (0.7-2.7) | 10.2 (5.3-14.4) | 2 (1-2.8) | 1.38% (0.77-2.5) | | 0.23 (0.16-0.3) | |
| Somalia | | 24.8 (9-55.5) | | 0.5 (0.2-0.9) | 57.9 (24.6-103.7) | 0.5 (0.2-0.7) | 1.34% (0.56-2.65) | | -0.03 (-0.1-0.04) | |
| South Africa | | 342.5 (214-474.1) | | 1.3 (0.8-1.8) | 671 (442.8-911.8) | 1.3 (0.9-1.8) | 0.96% (0.57-1.36) | | 0.16 (0.03-0.29) | |
| South Sudan | | 24.6 (10.1-46) | | 0.5 (0.2-0.9) | 38.1 (16.9-64.1) | 0.6 (0.3-0.9) | 0.55% (0.08-1.26) | | 0.18 (0.02-0.34) | |
| Spain | | 845.3 (789.7-898.6) | | 1.7 (1.6-1.8) | 2052.2 (1771.8-2305.4) | 2.3 (2-2.5) | 1.43% (1.13-1.73) | | 1 (0.83-1.17) | |
| Sri Lanka | | 236.4 (151-319.5) | | 1.7 (1.1-2.3) | 338.4 (205-479.1) | 1.3 (0.8-1.9) | 0.43% (-0.17-1.18) | | -0.79 (-0.95 to -0.63) | |
| Sudan | | 338.2 (164.8-657.3) | | 2.4 (1.2-4.2) | 716 (422.4-1106.6) | 2.5 (1.5-3.7) | 1.12% (0.21-2.67) | | 0.17 (0.11-0.23) | |
| Suriname | | 3.8 (2.5-5) | | 1.1 (0.8-1.5) | 7.2 (4.9-10) | 1.2 (0.8-1.6) | 0.92% (0.43-1.55) | | 0.51 (0.37-0.65) | |
| Sweden | | 296.5 (274-318.9) | | 2.2 (2-2.4) | 492 (425.4-556) | 2.3 (2-2.6) | 0.66% (0.44-0.89) | | 0.11 (-0.06-0.28) | |
| Switzerland | | 308.8 (281.7-339.1) | | 3.4 (3.1-3.8) | 546.7 (478.9-606.5) | 3.5 (3.1-3.9) | 0.77% (0.51-1) | | 0.52 (0.3-0.73) | |
| Taiwan (Province of China) | | 299.6 (262.4-350.9) | | 1.7 (1.5-2) | 921.9 (835.5-1004) | 2.6 (2.3-2.8) | 2.08% (1.61-2.66) | | 1.4 (1.11-1.7) | |
| Tajikistan | | 57.8 (34.7-85.6) | | 1.2 (0.8-1.7) | 88.7 (51.3-142.4) | 1 (0.6-1.5) | 0.53% (-0.14-1.46) | | -0.9 (-1.04 to -0.76) | |
| Thailand | | 1080.7 (748.5-1445.3) | | 2.5 (1.7-3.2) | 3025.3 (1413.1-4199.1) | 3.3 (1.6-4.5) | 1.8% (0.61-3.18) | | 0.78 (0.68-0.88) | |
| Timor-Leste | | 9.1 (4.2-17) | | 1.8 (0.9-2.8) | 19.3 (12.7-26.8) | 1.8 (1.2-2.5) | 1.11% (0.2-2.61) | | 0.04 (-0.18-0.25) | |
| Togo | | 7.1 (3.7-12.6) | | 0.3 (0.2-0.4) | 19.8 (10.1-28.1) | 0.3 (0.2-0.5) | 1.79% (0.46-3.66) | | 1.01 (0.92-1.09) | |
| Tokelau | | 0 (0-0) | | 1.9 (0.9-2.7) | 0 (0-0.1) | 3.4 (1.8-5.2) | 0.67% (0.21-1.39) | | 0.32 (-0.3-0.94) | |
| Tonga | | 1.2 (0.7-1.6) | | 1.6 (0.9-2.1) | 1.7 (1-2.3) | 1.8 (1-2.4) | 0.38% (0-0.88) | | 0.38 (0.27-0.49) | |
| Trinidad and Tobago | | 16.6 (15-18.3) | | 1.6 (1.5-1.8) | 32.9 (24.9-42.1) | 1.9 (1.5-2.5) | 0.98% (0.42-1.62) | | 0.78 (0.66-0.9) | |
| Tunisia | | 88.4 (63.4-142.7) | | 1.4 (1-2.3) | 169.5 (111.1-275.3) | 1.3 (0.9-2.1) | 0.92% (0.35-1.69) | | -0.32 (-0.35 to -0.29) | |
| Turkey | | 1769.3 (1129.2-2458.9) | | 4 (2.6-5.2) | 2605.4 (1826.5-3379.9) | 2.9 (2-3.7) | 0.47% (0.06-1.04) | | -1.15 (-1.37 to -0.93) | |
| Turkmenistan | | 42.4 (34.9-49.7) | | 1.2 (1-1.4) | 62.2 (46.9-81.5) | 1.2 (0.9-1.6) | 0.47% (0.08-0.96) | | 0.07 (-0.22-0.36) | |
| Tuvalu | | 0.2 (0.1-0.2) | | 1.9 (0.9-2.8) | 0.2 (0.1-0.3) | 1.8 (1-2.5) | 0.34% (0-0.85) | | -0.09 (-0.12 to -0.07) | |
| Uganda | | 48.8 (25-80.6) | | 0.4 (0.2-0.6) | 134.2 (72.5-206.8) | 0.4 (0.2-0.7) | 1.75% (0.41-3.42) | | 0.4 (0.28-0.51) | |
| Ukraine | | 1215.3 (1039.2-1391.7) | | 2.1 (1.8-2.4) | 768.4 (569-1011.2) | 1.2 (0.9-1.6) | -0.37% (-0.56 to -0.14) | | -2.2 (-2.45 to -1.95) | |
| United Arab Emirates | | 27.4 (16.8-38.3) | | 3.3 (1.8-4.6) | 133.2 (83.2-184.5) | 2.4 (1.5-3.2) | 3.87% (2.51-5.89) | | 0.13 (-0.2-0.46) | |
| United Kingdom | | 2235.1 (2158.2-2302.9) | | 2.7 (2.6-2.8) | 3580.1 (3282.4-3732.4) | 2.9 (2.7-3) | 0.6% (0.52-0.67) | | 0.26 (0.12-0.39) | |
| United States | 10694 (10075.1-11004.9) | | 3.5 (3.3-3.6) | | 21533 (19545.6-22516.3) | 3.8 (3.5-4) | | 1.01% (0.93-1.07) | | 0.37 (0.14-0.61) |
| Uruguay | | 71.6 (65.3-78.3) | | 2 (1.9-2.2) | 109.9 (97.3-122.4) | 2.4 (2.1-2.6) | 0.53% (0.33-0.75) | | 0.38 (0.24-0.52) | |
| Uzbekistan | | 257.9 (201.3-316.7) | | 1.4 (1.1-1.7) | 378.5 (310.2-467) | 1.1 (0.9-1.4) | 0.47% (0.09-1.14) | | -0.58 (-0.9 to -0.26) | |
| Vanuatu | | 1.7 (0.8-2.5) | | 1.7 (0.7-2.4) | 4.2 (2.2-5.8) | 1.7 (0.9-2.4) | 1.4% (0.86-2.2) | | 0.04 (-0.02-0.1) | |
| Yemen | | 167.7 (78.1-312.5) | | 2.2 (1-3.6) | 450.9 (239.1-671.2) | 2.3 (1.2-3.4) | 1.69% (0.6-3.62) | | 0.19 (0.12-0.27) | |
| Zambia | | 37.2 (16.8-65.1) | | 0.6 (0.3-0.9) | 94.5 (50.4-142.5) | 0.7 (0.4-1) | 1.54% (-0.01-3.73) | | 0.61 (0.5-0.72) | |
| Zimbabwe | | 65.8 (41.4-100.5) | | 1.2 (0.8-1.9) | 149.5 (91.2-234.3) | 1.5 (1-2.5) | 1.27% (0.54-2.27) | | 1.05 (0.66-1.45) | |
