# Supplementary material for: Global, regional, and national burden of acute myeloid leukemia, 1990–2021: a systematic analysis for the global burden of disease study 2021
Source: Biomark Res. 2024 Sep 11;12:101. doi: 10.1186/s40364-024-00649-y (PMC11389310; doi:10.1186/s40364-024-00649-y)
Supplement: Supplementary file 2 — Supplementary Material 2 [file 40364_2024_649_MOESM2_ESM.docx]

Table S2 The death cases and ASDR of AML in 1990 and 2021, and its temporal trends from 1990 to 2021 in 204 countries or territories

| location | Num_1990 | ASDR_1990 | Num_2021 | ASDR_2021 | Num_change | EAPC_CI |
| --- | --- | --- | --- | --- | --- | --- |
| Afghanistan | 281.1 (120.2-573.9) | 3.7 (1.6-7.1) | 597.1 (300.5-1092.2) | 3.9 (1.9-6.9) | 1.12% (0.53-2.06) | 0.08 (0.01-0.15) |
| Albania | 38.6 (27.1-58.7) | 1.6 (1.1-2.5) | 52.9 (33.5-79) | 1.3 (0.9-2) | 0.37% (-0.11-1.04) | -0.43 (-0.52 to -0.35) |
| Algeria | 204.1 (126.6-281.2) | 1.1 (0.6-1.5) | 321.1 (205.3-502.5) | 0.9 (0.6-1.3) | 0.57% (0.08-1.37) | -0.81 (-0.95 to -0.68) |
| American Samoa | 0.8 (0.5-1) | 2.3 (1.3-3.1) | 0.6 (0.4-0.9) | 1.2 (0.9-2) | -0.26% (-0.52-0.56) | -2.1 (-2.41 to -1.79) |
| Andorra | 1.9 (1.2-2.9) | 3.3 (2.1-5.2) | 3.3 (1.9-4.8) | 2.3 (1.3-3.3) | 0.76% (0.03-1.97) | -0.77 (-0.88 to -0.67) |
| Angola | 50 (22.4-104.2) | 0.8 (0.4-1.2) | 134.2 (72.2-201.7) | 0.7 (0.4-1.1) | 1.69% (0.24-3.8) | -0.17 (-0.21 to -0.13) |
| Antigua and Barbuda | 0.8 (0.7-0.9) | 1.4 (1.3-1.6) | 1.8 (1.7-2) | 1.9 (1.7-2) | 1.29% (0.98-1.6) | 1.25 (1.09-1.41) |
| Argentina | 624.6 (569.5-689.8) | 1.9 (1.7-2.1) | 1012.3 (923.6-1122.3) | 1.9 (1.7-2.1) | 0.62% (0.42-0.84) | 0.45 (0.27-0.64) |
| Armenia | 28.6 (22.6-35.2) | 0.9 (0.7-1.1) | 49.8 (41.2-60) | 1.3 (1.1-1.5) | 0.74% (0.28-1.42) | 1.01 (0.52-1.5) |
| Australia | 422.2 (392.2-454.2) | 2.2 (2.1-2.4) | 1351.5 (1198.8-1494.6) | 3 (2.7-3.3) | 2.2% (1.87-2.58) | 0.52 (0.28-0.76) |
| Austria | 209 (193-224.6) | 1.9 (1.8-2) | 399 (353.7-442.3) | 2.2 (2-2.4) | 0.91% (0.71-1.13) | 1.12 (0.98-1.27) |
| Azerbaijan | 102.9 (71-158.1) | 1.5 (1.1-2.3) | 135.8 (91.6-216.2) | 1.3 (0.9-2) | 0.32% (-0.11-0.95) | -0.32 (-0.42 to -0.21) |
| Bahrain | 6.7 (4.4-9) | 2.9 (1.7-3.8) | 18.3 (11.8-28.2) | 2 (1.2-2.8) | 1.71% (0.83-3.09) | -1.12 (-1.33 to -0.9) |
| Bangladesh | 865.5 (460.1-1711.4) | 1 (0.6-1.7) | 1297.7 (815-1920.1) | 0.9 (0.6-1.3) | 0.5% (-0.38-1.7) | -0.35 (-0.41 to -0.28) |
| Barbados | 4.9 (4.5-5.3) | 1.8 (1.7-2) | 9.8 (7.6-12.1) | 2.3 (1.8-2.9) | 1% (0.54-1.48) | 1.26 (1.12-1.39) |
| Belarus | 137 (112.9-174) | 1.2 (1-1.5) | 248.6 (199.9-301.6) | 1.8 (1.5-2.2) | 0.82% (0.34-1.39) | 0.78 (0.46-1.1) |
| Belgium | 333.9 (300.4-367.7) | 2.3 (2.1-2.5) | 646.2 (557.8-729.6) | 2.8 (2.5-3.1) | 0.94% (0.69-1.25) | 0.97 (0.78-1.17) |
| Belize | 0.5 (0.5-0.6) | 0.3 (0.3-0.4) | 1.8 (1.6-2) | 0.5 (0.4-0.5) | 2.45% (1.78-3.22) | 1.85 (1.59-2.1) |
| Benin | 12 (5.9-24.6) | 0.3 (0.2-0.5) | 33 (15.8-46.4) | 0.3 (0.2-0.5) | 1.76% (0.33-3.68) | 0.48 (0.41-0.56) |
| Bermuda | 1.6 (1.5-1.9) | 2.7 (2.4-3.1) | 2 (1.7-2.5) | 1.9 (1.5-2.2) | 0.24% (0-0.51) | -1.19 (-1.32 to -1.06) |
| Bhutan | 4.2 (1.9-7.7) | 1 (0.5-1.6) | 6.4 (3.9-10.8) | 1 (0.6-1.7) | 0.53% (-0.27-1.86) | 0.35 (0.25-0.46) |
| Bosnia and Herzegovina | 60.8 (46-86.5) | 1.5 (1.1-2.1) | 87.5 (56.8-121.7) | 1.5 (1-2.1) | 0.44% (0-1.15) | 0.11 (-0.01-0.23) |
| Botswana | 9 (5.4-14.3) | 1.3 (0.8-1.9) | 20.1 (11.6-29.9) | 1.2 (0.7-1.7) | 1.24% (0.54-2.41) | 0.07 (-0.04-0.18) |
| Brazil | 2061.9 (1990.6-2135.3) | 1.8 (1.7-1.9) | 4490.7 (4175.1-4722) | 1.9 (1.7-2) | 1.18% (1.06-1.29) | 0.29 (0.21-0.37) |
| Bulgaria | 157.3 (144.9-171.5) | 1.5 (1.3-1.6) | 301.6 (242.9-368.7) | 2.5 (2-3) | 0.92% (0.51-1.42) | 1.83 (1.59-2.07) |
| Burkina Faso | 22.9 (10.9-45.1) | 0.3 (0.2-0.5) | 61.4 (27.6-92.2) | 0.4 (0.2-0.5) | 1.69% (0.43-3.3) | 0.57 (0.44-0.69) |
| Burundi | 24.9 (9.9-44.5) | 0.6 (0.2-0.9) | 43.1 (17.9-69.6) | 0.5 (0.2-0.8) | 0.73% (-0.19-1.96) | -0.31 (-0.37 to -0.25) |
| Cambodia | 171.7 (86.7-332.8) | 2.6 (1.3-4.3) | 347.7 (207.8-490.1) | 2.5 (1.6-3.5) | 1.02% (0.15-2.25) | -0.05 (-0.07 to -0.02) |
| Cameroon | 26.5 (14.3-50.7) | 0.3 (0.2-0.6) | 81.4 (41.7-118.5) | 0.4 (0.2-0.6) | 2.07% (0.69-3.98) | 0.57 (0.5-0.63) |
| Canada | 722.3 (675.4-764.8) | 2.3 (2.1-2.4) | 1721.5 (1546.8-1884.1) | 2.4 (2.2-2.6) | 1.38% (1.18-1.59) | 0.45 (0.31-0.59) |
| Central African Republic | 14.2 (6.7-29.4) | 0.8 (0.4-1.3) | 24.8 (13-40.4) | 0.7 (0.4-1.1) | 0.75% (0.21-1.56) | -0.2 (-0.26 to -0.15) |
| Chad | 11.8 (5.8-25.4) | 0.2 (0.1-0.4) | 40.5 (17.2-67.7) | 0.4 (0.2-0.5) | 2.42% (1.1-4.28) | 1.24 (1.13-1.36) |
| Chile | 166.2 (155.6-176.9) | 1.4 (1.3-1.6) | 361.9 (328.2-394.8) | 1.5 (1.4-1.6) | 1.18% (0.96-1.42) | 0.3 (0.17-0.42) |
| China | 14853.1 (8014.1-23401.9) | 1.4 (0.8-2.2) | 15311.1 (10365.3-21401.3) | 0.9 (0.6-1.2) | 0.03% (-0.39-0.79) | -1.54 (-1.73 to -1.35) |
| Colombia | 327.1 (305.9-349.3) | 1.3 (1.2-1.4) | 837.8 (692.6-985) | 1.6 (1.3-1.9) | 1.56% (1.11-2.03) | 0.46 (0.35-0.56) |
| Comoros | 2 (0.8-2.9) | 0.6 (0.3-0.9) | 3.4 (1.9-5.1) | 0.6 (0.3-0.8) | 0.74% (0.03-1.79) | -0.03 (-0.15-0.08) |
| Congo | 13.2 (8-22.3) | 0.9 (0.5-1.3) | 27.5 (16.3-38.9) | 0.8 (0.4-1.1) | 1.08% (0.22-2.31) | -0.39 (-0.47 to -0.31) |
| Cook Islands | 0.1 (0.1-0.2) | 0.9 (0.5-1.2) | 0.2 (0.1-0.2) | 0.7 (0.4-0.9) | 0.18% (-0.13-0.64) | -1.03 (-1.08 to -0.97) |
| Costa Rica | 37.7 (34.9-41.9) | 1.7 (1.5-1.9) | 119.8 (105.6-134.3) | 2.3 (2-2.5) | 2.18% (1.74-2.64) | 0.87 (0.73-1) |
| Croatia | 92.6 (77.9-109.2) | 1.6 (1.3-1.9) | 183 (147.4-223) | 2.2 (1.8-2.6) | 0.98% (0.54-1.55) | 1.13 (0.99-1.26) |
| Cuba | 170.8 (159.9-185.7) | 1.6 (1.5-1.7) | 257.9 (219.8-296.8) | 1.6 (1.3-1.8) | 0.51% (0.26-0.79) | -0.01 (-0.13-0.12) |
| Cyprus | 25 (18.7-38.4) | 3.3 (2.5-5.3) | 51.7 (31.8-67.7) | 2.6 (1.6-3.3) | 1.06% (0.2-2.03) | -0.6 (-0.7 to -0.51) |
| Democratic Republic of the Congo | 163.5 (78.8-282.3) | 0.7 (0.3-1) | 330.1 (186.4-469.5) | 0.6 (0.4-0.9) | 1.02% (0.17-2.15) | -0.42 (-0.51 to -0.33) |
| Denmark | 246.3 (230.3-263.5) | 3.3 (3.1-3.5) | 304.5 (269.8-343.2) | 2.6 (2.3-2.9) | 0.24% (0.09-0.41) | -0.83 (-0.99 to -0.67) |
| Djibouti | 1.5 (0.7-2.4) | 0.5 (0.2-0.8) | 4.9 (2.4-8.1) | 0.5 (0.3-0.9) | 2.23% (1.09-3.91) | 0.49 (0.38-0.61) |
| Dominica | 1.1 (0.9-1.5) | 1.8 (1.4-2.4) | 1.4 (1-1.9) | 1.8 (1.3-2.5) | 0.24% (-0.1-0.66) | 0.41 (0.33-0.49) |
| Dominican Republic | 73.8 (51.8-106.9) | 1.2 (0.8-1.8) | 127.7 (90.7-194.7) | 1.2 (0.9-1.9) | 0.73% (0.24-1.42) | 0.21 (0.08-0.34) |
| Ecuador | 98.2 (90.6-108.1) | 1.3 (1.2-1.5) | 305.5 (243.4-377) | 1.8 (1.4-2.2) | 2.11% (1.45-2.93) | 1.79 (1.52-2.06) |
| Egypt | 715.3 (448.8-1390.5) | 1.9 (1.2-3.8) | 2089.4 (1434.3-3066.2) | 2.9 (1.9-4.6) | 1.92% (0.72-3.55) | 1.57 (1.34-1.8) |
| El Salvador | 56.8 (37.7-88.9) | 1.2 (0.9-1.9) | 92.6 (56.8-121.4) | 1.5 (0.9-1.9) | 0.63% (0.12-1.25) | 0.39 (0.29-0.49) |
| Equatorial Guinea | 2.1 (1-3.9) | 0.8 (0.4-1.2) | 6.2 (3.1-9.9) | 0.7 (0.4-1.1) | 1.89% (0.35-4.08) | -0.15 (-0.21 to -0.09) |
| Eritrea | 11.8 (5.1-20.8) | 0.5 (0.2-0.8) | 25.9 (13.7-37) | 0.6 (0.3-0.8) | 1.2% (0.12-2.71) | 0.46 (0.4-0.52) |
| Estonia | 25.6 (22.4-29) | 1.5 (1.3-1.7) | 41.9 (34.6-49.7) | 1.9 (1.6-2.2) | 0.64% (0.36-0.97) | 1.32 (0.97-1.68) |
| Ethiopia | 455.8 (185.1-931.9) | 1.3 (0.5-2.2) | 731.2 (391.9-1161.5) | 1 (0.6-1.7) | 0.6% (-0.39-1.97) | -0.83 (-0.91 to -0.74) |
| Fiji | 22.8 (13.9-29.9) | 4.5 (2.7-5.9) | 33.3 (18.1-45.8) | 4.3 (2.3-5.8) | 0.46% (0.07-1.03) | -0.18 (-0.28 to -0.08) |
| Finland | 116.2 (106.5-125.8) | 1.7 (1.6-1.9) | 189.4 (165-214) | 1.6 (1.4-1.8) | 0.63% (0.41-0.87) | -0.27 (-0.37 to -0.17) |
| France | 1647.6 (1539.2-1767.7) | 2.1 (2-2.3) | 3487.8 (2964.5-3905.6) | 2.5 (2.2-2.7) | 1.12% (0.84-1.39) | 0.57 (0.42-0.73) |
| Gabon | 6 (3.3-8.4) | 0.9 (0.5-1.2) | 10.1 (5.9-14.9) | 0.8 (0.5-1.1) | 0.69% (0.12-1.44) | 0.01 (-0.12-0.13) |
| Georgia | 70.4 (57.5-89.9) | 1.2 (1-1.6) | 91.7 (80.1-105.7) | 1.9 (1.6-2.1) | 0.3% (-0.03-0.65) | 1.12 (0.56-1.68) |
| Germany | 2352.1 (2127.4-2576.7) | 2 (1.9-2.2) | 4944 (4380.3-5434.7) | 2.6 (2.3-2.8) | 1.1% (0.85-1.38) | 0.7 (0.61-0.79) |
| Ghana | 70.1 (32.6-100.6) | 0.6 (0.3-0.9) | 95.1 (60.7-167.8) | 0.4 (0.2-0.7) | 0.36% (-0.29-2.25) | -1.59 (-1.9 to -1.28) |
| Greece | 315.2 (297-335.4) | 2.2 (2.1-2.3) | 740.8 (670.3-795.9) | 3.2 (2.9-3.4) | 1.35% (1.16-1.54) | 1.07 (0.97-1.17) |
| Greenland | 0.7 (0.4-0.8) | 1.9 (1.3-2.3) | 0.8 (0.5-1.1) | 1.3 (0.7-1.8) | 0.15% (-0.14-0.65) | -1.14 (-1.2 to -1.07) |
| Grenada | 0.7 (0.6-0.9) | 0.9 (0.7-1.1) | 1.2 (1-1.4) | 1.1 (1-1.3) | 0.73% (0.33-1.28) | 0.7 (0.56-0.85) |
| Guam | 1.6 (1.2-2.2) | 1.7 (1.3-2.3) | 3.1 (2-3.9) | 1.7 (1.1-2.1) | 0.95% (0.42-1.6) | -0.37 (-0.7 to -0.05) |
| Guatemala | 65.2 (52.5-76.2) | 0.9 (0.7-1) | 143.4 (121.8-165.6) | 1.1 (0.9-1.2) | 1.2% (0.73-1.9) | 0.72 (0.56-0.87) |
| Guinea | 6.9 (3-14.7) | 0.1 (0.1-0.2) | 10.7 (5.6-17.5) | 0.1 (0.1-0.2) | 0.56% (-0.16-1.61) | -0.26 (-0.28 to -0.23) |
| Guinea-Bissau | 2.7 (1.4-6) | 0.4 (0.2-0.7) | 5.3 (2.8-7.3) | 0.4 (0.2-0.6) | 0.95% (0.02-2.29) | 0.62 (0.54-0.71) |
| Guyana | 1.6 (1.3-1.9) | 0.3 (0.2-0.3) | 2.8 (2.1-3.6) | 0.4 (0.3-0.5) | 0.79% (0.23-1.54) | 1.84 (1.69-1.99) |
| Haiti | 85.8 (42.5-182.5) | 1.6 (0.9-3) | 155 (85.6-251.3) | 1.5 (0.9-2.3) | 0.81% (0.12-1.82) | -0.04 (-0.1-0.02) |
| Honduras | 52.3 (29.2-82.5) | 1.4 (0.9-2.1) | 114.3 (70.4-173.9) | 1.5 (1-2.3) | 1.19% (0.49-2.29) | 0.38 (0.31-0.44) |
| Hungary | 284.6 (265.7-304.2) | 2.1 (1.9-2.2) | 397.8 (335.4-475) | 2.2 (1.9-2.7) | 0.4% (0.15-0.69) | 0.41 (0.32-0.5) |
| Iceland | 5.7 (5.1-6.2) | 2 (1.8-2.2) | 12.7 (11-14.5) | 2.3 (2-2.5) | 1.22% (0.89-1.64) | 0.04 (-0.09-0.17) |
| India | 5249.8 (3288.7-8407.7) | 0.8 (0.5-1.2) | 10981.1 (8158.9-15119.9) | 0.9 (0.7-1.2) | 1.09% (0.28-2.03) | 0.23 (0.16-0.29) |
| Indonesia | 2737.5 (1784.1-4170.4) | 2.1 (1.4-3) | 5453.2 (3927.9-7455.9) | 2.2 (1.6-3) | 0.99% (0.45-1.69) | 0.62 (0.49-0.75) |
| Iraq | 219.4 (115.9-499.4) | 1.8 (0.9-3.7) | 514.4 (325-939.4) | 1.7 (1.1-3.2) | 1.34% (0.52-2.93) | -0.01 (-0.07-0.05) |
| Ireland | 80 (75.2-85.1) | 2 (1.8-2.1) | 142.1 (127.4-157.8) | 1.8 (1.7-2) | 0.78% (0.6-0.99) | 0.43 (0.22-0.64) |
| Israel | 134.4 (121.4-147.2) | 2.8 (2.5-3) | 335.6 (294.6-370.8) | 2.7 (2.4-3) | 1.5% (1.2-1.84) | 0.31 (0.18-0.45) |
| Italy | 1496.3 (1410.7-1560.5) | 1.9 (1.8-2) | 3522.5 (3097.1-3803.9) | 2.5 (2.2-2.6) | 1.35% (1.15-1.51) | 1.22 (1.06-1.39) |
| Jamaica | 12.6 (10.9-15.1) | 0.6 (0.5-0.7) | 31.1 (23.7-40.3) | 1 (0.8-1.3) | 1.46% (0.72-2.39) | 1.61 (1.3-1.91) |
| Japan | 3019.9 (2906.3-3088.7) | 1.9 (1.9-2) | 5578.7 (4835.2-6001) | 1.6 (1.5-1.7) | 0.85% (0.65-0.96) | -0.49 (-0.64 to -0.34) |
| Jordan | 106.4 (79.9-141.7) | 5 (3.8-6.7) | 305.6 (216.2-406) | 3.5 (2.5-4.7) | 1.87% (1.07-2.94) | -1.15 (-1.29 to -1.01) |
| Kazakhstan | 223.2 (185.6-274.5) | 1.5 (1.2-1.8) | 209.9 (171.5-250.9) | 1.1 (0.9-1.3) | -0.06% (-0.25-0.19) | -0.6 (-0.8 to -0.4) |
| Kenya | 57.6 (33.5-85.3) | 0.4 (0.2-0.6) | 157.8 (105.5-218.9) | 0.5 (0.3-0.7) | 1.74% (0.69-3) | 0.83 (0.73-0.92) |
| Kiribati | 0.8 (0.3-1.4) | 1.4 (0.5-2.2) | 1.4 (0.6-2.3) | 1.4 (0.6-2.3) | 0.75% (0.21-1.36) | 0.18 (0.13-0.22) |
| Kuwait | 13.1 (11.7-14.6) | 1.5 (1.3-1.7) | 38.6 (30.7-47) | 1.1 (0.9-1.4) | 1.96% (1.35-2.6) | -0.85 (-1.2 to -0.51) |
| Kyrgyzstan | 32.4 (26.8-40.1) | 0.8 (0.6-0.9) | 60.6 (50-73.1) | 1 (0.8-1.2) | 0.87% (0.38-1.45) | 0.78 (0.46-1.1) |
| Latvia | 66.8 (58.6-76.9) | 2.2 (1.9-2.5) | 53 (44.3-62.5) | 1.7 (1.4-1.9) | -0.21% (-0.35 to -0.04) | -0.76 (-0.97 to -0.54) |
| Lebanon | 66 (44-111.5) | 2.9 (2-4.7) | 148.6 (107.7-200.6) | 2.4 (1.8-3.3) | 1.25% (0.45-2.39) | -0.3 (-0.38 to -0.23) |
| Lesotho | 9.8 (6.3-16) | 1 (0.6-1.6) | 20.2 (12.8-36.5) | 1.6 (1-2.7) | 1.06% (0.29-2.26) | 1.62 (1.43-1.81) |
| Liberia | 6.2 (2.8-14.6) | 0.3 (0.2-0.5) | 12.1 (5.7-17.6) | 0.4 (0.2-0.5) | 0.97% (-0.11-2.67) | 0.48 (0.32-0.64) |
| Libya | 75.2 (51.3-117.3) | 3 (2.1-4.9) | 161.8 (98.2-274.2) | 2.9 (1.8-4.7) | 1.15% (0.28-2.35) | -0.06 (-0.14-0.02) |
| Lithuania | 52.2 (46.1-59.1) | 1.3 (1.1-1.5) | 107.3 (90.2-123.9) | 2.2 (1.9-2.6) | 1.05% (0.71-1.46) | 2.17 (1.94-2.4) |
| Luxembourg | 15.5 (14.6-16.5) | 3.1 (2.9-3.2) | 33.5 (30-37) | 3.2 (2.9-3.5) | 1.16% (0.91-1.43) | 0.32 (0.2-0.44) |
| Madagascar | 44.5 (20.8-66) | 0.5 (0.2-0.7) | 85 (41.8-120.9) | 0.4 (0.2-0.6) | 0.91% (0.1-1.83) | -0.44 (-0.52 to -0.35) |
| Malawi | 17.1 (5.4-31.5) | 0.2 (0.1-0.3) | 22.3 (10.4-38.7) | 0.2 (0.1-0.2) | 0.3% (-0.31-1.73) | -0.39 (-0.48 to -0.3) |
| Malaysia | 235.5 (145.1-308.4) | 1.9 (1.2-2.5) | 528.9 (370.3-739.3) | 1.8 (1.3-2.6) | 1.25% (0.72-2.11) | 0 (-0.09-0.09) |
| Maldives | 2.6 (1.5-5.2) | 2.1 (1.3-3.6) | 4.9 (3.1-7.1) | 1.3 (0.9-1.8) | 0.87% (-0.16-2.3) | -1.39 (-1.49 to -1.28) |
| Mali | 20 (8-40.2) | 0.3 (0.1-0.4) | 36.7 (20-57.4) | 0.2 (0.1-0.3) | 0.84% (0.13-2.09) | -0.54 (-0.57 to -0.5) |
| Malta | 8.6 (7.8-9.5) | 2.1 (1.9-2.3) | 22.1 (19.4-24.9) | 2.4 (2.1-2.7) | 1.55% (1.18-1.97) | 0.49 (0.33-0.64) |
| Marshall Islands | 0.6 (0.3-0.8) | 1.9 (0.9-2.7) | 0.9 (0.5-1.3) | 2.1 (1.1-3) | 0.6% (0.15-1.15) | 0.54 (0.47-0.6) |
| Mauritania | 4.6 (2.7-8.3) | 0.3 (0.2-0.5) | 10.1 (5.7-14.2) | 0.3 (0.2-0.5) | 1.18% (0.25-2.36) | 0.5 (0.44-0.56) |
| Mauritius | 6 (5.5-6.6) | 0.7 (0.6-0.7) | 14.9 (13.6-16.1) | 0.9 (0.8-1) | 1.48% (1.15-1.8) | 1.84 (-0.19-3.91) |
| Mexico | 944.3 (915.9-980.1) | 1.4 (1.3-1.4) | 1914.3 (1690.2-2120.3) | 1.5 (1.3-1.7) | 1.03% (0.8-1.26) | 0.24 (0.13-0.35) |
| Mongolia | 21.8 (13.2-36.2) | 1.3 (0.8-2) | 36.3 (25-48.5) | 1.2 (0.9-1.6) | 0.66% (-0.01-1.81) | 0 (-0.08-0.07) |
| Montenegro | 10.9 (8.2-15.1) | 1.8 (1.3-2.4) | 16.2 (11.4-23.1) | 1.8 (1.3-2.5) | 0.49% (0.08-1.08) | -0.01 (-0.09-0.08) |
| Morocco | 90.5 (60-145) | 0.5 (0.3-0.8) | 178.5 (118.5-288.1) | 0.5 (0.3-0.8) | 0.97% (0.4-1.76) | -0.01 (-0.08-0.05) |
| Mozambique | 99.8 (34-187.6) | 0.8 (0.3-1.3) | 170.6 (84.9-293.2) | 0.8 (0.4-1.2) | 0.71% (-0.14-2.59) | -0.19 (-0.28 to -0.11) |
| Myanmar | 807.1 (415.5-1615.1) | 2.6 (1.4-4.9) | 1119.3 (795.2-1605.3) | 2.2 (1.6-3.2) | 0.39% (-0.22-1.44) | -0.37 (-0.5 to -0.25) |
| Namibia | 7.8 (5.1-11.6) | 0.9 (0.6-1.4) | 15.9 (10.3-24.4) | 0.9 (0.6-1.5) | 1.04% (0.45-2.04) | 0.16 (0.04-0.27) |
| Nepal | 134.6 (65.9-262.6) | 0.9 (0.5-1.6) | 241.3 (163.5-371.2) | 0.9 (0.6-1.4) | 0.79% (0.01-2.17) | 0.01 (-0.1-0.13) |
| Netherlands | 437.7 (404.4-467.6) | 2.3 (2.1-2.4) | 824 (728.3-922.7) | 2.4 (2.1-2.6) | 0.88% (0.69-1.12) | 0.46 (0.34-0.57) |
| New Zealand | 103.7 (93.7-112.1) | 2.7 (2.4-2.9) | 167.6 (147.9-185.7) | 2.1 (1.9-2.3) | 0.62% (0.42-0.83) | -0.25 (-0.51-0) |
| Nicaragua | 29.2 (18.2-45.3) | 0.9 (0.7-1.3) | 50.6 (33-67) | 0.9 (0.6-1.2) | 0.73% (0.1-1.37) | 0.09 (-0.02-0.19) |
| Niger | 19 (7.3-47.7) | 0.3 (0.1-0.5) | 49 (19.9-75.3) | 0.3 (0.1-0.5) | 1.58% (0.07-4.01) | 0.28 (0.2-0.36) |
| Nigeria | 213.9 (108.9-420.1) | 0.3 (0.2-0.5) | 456.5 (255.4-617.8) | 0.3 (0.2-0.4) | 1.13% (0.23-2.14) | 0.08 (0.04-0.13) |
| Northern Mariana Islands | 0.6 (0.3-0.8) | 2.1 (1.1-2.9) | 0.4 (0.2-0.9) | 0.9 (0.6-2) | -0.3% (-0.66-0.65) | -2.4 (-2.7 to -2.09) |
| Norway | 155.9 (147.2-163.3) | 2.5 (2.3-2.6) | 230.5 (207.9-249.2) | 2.3 (2.1-2.5) | 0.48% (0.36-0.6) | -0.43 (-0.58 to -0.29) |
| Oman | 23.8 (14-33.4) | 2 (1-2.8) | 39.7 (26.3-53.6) | 1.4 (0.9-1.9) | 0.67% (0.13-1.59) | -0.42 (-0.54 to -0.3) |
| Pakistan | 892 (554.6-1476.8) | 1 (0.6-1.6) | 2039.4 (1399.6-3208.8) | 1.2 (0.8-1.8) | 1.29% (0.63-2.48) | 0.71 (0.57-0.85) |
| Palestine | 11.9 (7.1-20) | 0.9 (0.5-1.4) | 24.9 (15.9-37.1) | 0.7 (0.4-1.1) | 1.1% (0.35-2.17) | -0.56 (-0.63 to -0.48) |
| Panama | 25.8 (23.6-28.5) | 1.3 (1.2-1.4) | 76.2 (60.9-91.9) | 1.8 (1.4-2.1) | 1.96% (1.33-2.59) | 1.18 (1.07-1.3) |
| Papua New Guinea | 49 (18.3-75.4) | 1.6 (0.6-2.6) | 125 (60-192.9) | 1.6 (0.7-2.6) | 1.55% (0.8-2.56) | 0 (-0.03-0.03) |
| Paraguay | 37.1 (26.3-52.5) | 1.2 (0.9-1.7) | 99.3 (66-134.6) | 1.6 (1.1-2.2) | 1.68% (0.89-2.74) | 1.06 (0.94-1.18) |
| Peru | 253.9 (169.7-379.5) | 1.5 (1-2.2) | 523.9 (298.4-718.4) | 1.5 (0.9-2.1) | 1.06% (0.23-2.05) | 0.31 (0.23-0.4) |
| Philippines | 1017.5 (747.6-1413.1) | 2.4 (1.8-3) | 2020.1 (1621.9-2624.5) | 2.2 (1.8-2.9) | 0.99% (0.54-1.49) | -0.18 (-0.25 to -0.12) |
| Poland | 766.3 (695.1-890.2) | 1.8 (1.7-2.1) | 1287.7 (1171.9-1399.6) | 1.9 (1.7-2.1) | 0.68% (0.45-0.94) | 0.61 (0.44-0.78) |
| Portugal | 241.9 (224.4-259.2) | 1.9 (1.8-2.1) | 521.6 (453.2-579.3) | 2.2 (2-2.4) | 1.16% (0.9-1.43) | 0.79 (0.62-0.96) |
| Puerto Rico | 83.5 (77.4-90.5) | 2.3 (2.1-2.5) | 138.8 (113-165.3) | 2.3 (1.9-2.7) | 0.66% (0.35-1.01) | -0.16 (-0.27 to -0.04) |
| Qatar | 4.3 (2.6-5.9) | 2.3 (1.3-3.3) | 16.9 (11.1-25.9) | 1.3 (0.8-1.9) | 2.94% (1.57-5.24) | -1.27 (-1.57 to -0.97) |
| Romania | 290.5 (263.1-321.4) | 1.2 (1-1.3) | 543.5 (469.2-619.7) | 1.7 (1.4-1.9) | 0.87% (0.57-1.2) | 1.32 (1.23-1.41) |
| Russian Federation | 2097.5 (1897.9-2294.8) | 1.3 (1.2-1.4) | 2681.2 (2460.1-2906.2) | 1.3 (1.2-1.4) | 0.28% (0.12-0.43) | 0.15 (0.02-0.27) |
| Rwanda | 37.2 (16.1-69.8) | 0.7 (0.3-1.1) | 52 (26.6-79.9) | 0.5 (0.3-0.8) | 0.4% (-0.4-1.66) | -0.52 (-0.7 to -0.35) |
| Saint Kitts and Nevis | 0.1 (0.1-0.1) | 0.2 (0.2-0.3) | 0.2 (0.1-0.2) | 0.3 (0.2-0.3) | 0.92% (0.48-1.42) | 0.71 (0.53-0.89) |
| Saint Lucia | 1.3 (1.2-1.5) | 1.2 (1.1-1.4) | 2.5 (2-3) | 1.2 (1-1.4) | 0.9% (0.44-1.36) | 0.2 (0.07-0.34) |
| Saint Vincent and the Grenadines | 1.1 (1-1.2) | 1 (0.9-1.1) | 1.4 (1.2-1.6) | 1.1 (1-1.3) | 0.34% (0.12-0.59) | 0.83 (0.71-0.94) |
| Samoa | 3 (1.8-4.3) | 2.6 (1.4-3.6) | 4 (2.4-5.6) | 2.4 (1.4-3.4) | 0.3% (0.01-0.71) | -0.28 (-0.33 to -0.23) |
| Sao Tome and Principe | 0.3 (0.1-0.4) | 0.2 (0.2-0.3) | 0.3 (0.2-0.5) | 0.2 (0.1-0.4) | 0.3% (-0.22-1.27) | 0.12 (-0.04-0.27) |
| Saudi Arabia | 81.1 (52.1-137.1) | 0.8 (0.6-1.5) | 310.8 (207.3-545.5) | 1.1 (0.8-1.8) | 2.83% (1.54-5.43) | 1.35 (1.09-1.6) |
| Senegal | 18.3 (9.5-33.9) | 0.3 (0.2-0.5) | 40.1 (22.2-56.5) | 0.4 (0.2-0.5) | 1.19% (0.1-2.76) | 0.9 (0.8-1) |
| Serbia | 209.8 (151.9-282.5) | 2 (1.5-2.8) | 294 (184.7-377.8) | 1.9 (1.2-2.4) | 0.4% (0.04-0.81) | -0.15 (-0.2 to -0.09) |
| Seychelles | 1.1 (0.7-1.4) | 1.7 (1.1-2.2) | 1.5 (0.9-2) | 1.3 (0.8-1.8) | 0.36% (0.06-0.77) | -0.25 (-0.34 to -0.16) |
| Sierra Leone | 10.1 (4.8-23.3) | 0.3 (0.1-0.5) | 21.4 (10.1-30.6) | 0.3 (0.2-0.5) | 1.12% (0-2.82) | 0.69 (0.55-0.83) |
| Singapore | 56.4 (52.8-59.9) | 2.3 (2.1-2.5) | 127.4 (116.2-138.1) | 1.6 (1.5-1.8) | 1.26% (1.02-1.5) | -0.86 (-1.04 to -0.69) |
| Slovakia | 129.3 (101.8-190.3) | 2.2 (1.7-3.3) | 163.3 (93.8-220.1) | 1.9 (1.1-2.5) | 0.26% (-0.23-0.85) | -0.32 (-0.37 to -0.27) |
| Slovenia | 29.4 (26.2-33) | 1.2 (1.1-1.4) | 58.6 (48-69.6) | 1.4 (1.1-1.6) | 1% (0.63-1.47) | 0.74 (0.57-0.91) |
| Solomon Islands | 4.3 (1.8-6.5) | 1.9 (0.7-2.8) | 10.1 (5.2-14.3) | 2 (1-2.9) | 1.36% (0.77-2.49) | 0.25 (0.21-0.29) |
| Somalia | 24.5 (9-53.7) | 0.5 (0.2-0.9) | 57.3 (24.4-101.8) | 0.5 (0.2-0.8) | 1.34% (0.54-2.61) | -0.13 (-0.18 to -0.08) |
| South Africa | 339.1 (211.1-469.9) | 1.3 (0.8-1.8) | 665.1 (433.6-898) | 1.4 (0.9-1.8) | 0.96% (0.58-1.39) | 0.05 (-0.04-0.15) |
| South Sudan | 24.7 (10.1-46.1) | 0.6 (0.2-1) | 37.7 (16.6-62.9) | 0.6 (0.3-0.9) | 0.52% (0.06-1.24) | 0.06 (-0.04-0.16) |
| Spain | 799.7 (748.2-848.1) | 1.6 (1.5-1.7) | 1822.8 (1581.7-2045.8) | 1.9 (1.7-2.1) | 1.28% (1.01-1.57) | 0.78 (0.69-0.87) |
| Sri Lanka | 230.2 (147.2-312.6) | 1.7 (1.2-2.4) | 322.2 (195.5-453.7) | 1.3 (0.8-1.8) | 0.4% (-0.19-1.12) | -0.69 (-0.81 to -0.57) |
| Sudan | 337.3 (165.3-653.8) | 2.5 (1.3-4.3) | 690.4 (408-1076.8) | 2.5 (1.5-3.8) | 1.05% (0.16-2.48) | -0.04 (-0.09-0.01) |
| Suriname | 3.7 (2.5-5) | 1.1 (0.8-1.6) | 7.1 (4.8-9.9) | 1.2 (0.8-1.6) | 0.91% (0.43-1.54) | 0.44 (0.35-0.54) |
| Sweden | 284.1 (261.8-307.3) | 2 (1.9-2.2) | 444.4 (380.4-500.8) | 2 (1.7-2.3) | 0.56% (0.36-0.78) | -0.23 (-0.34 to -0.11) |
| Switzerland | 217.5 (196.6-237.2) | 2.3 (2.1-2.4) | 350.9 (303.9-392.6) | 2 (1.8-2.2) | 0.61% (0.39-0.84) | -0.9 (-1.24 to -0.55) |
| Taiwan (Province of China) | 275.7 (242.5-322.3) | 1.6 (1.4-1.9) | 812.7 (732.7-879.7) | 2.2 (2-2.4) | 1.95% (1.52-2.48) | 1.8 (1.53-2.07) |
| Tajikistan | 55.9 (33.8-82.6) | 1.2 (0.7-1.6) | 84.2 (48.5-136.6) | 0.9 (0.6-1.5) | 0.51% (-0.16-1.43) | -0.71 (-0.8 to -0.61) |
| Thailand | 1056.3 (738.5-1411.6) | 2.5 (1.7-3.3) | 2912.1 (1319.4-4091.3) | 3.1 (1.5-4.3) | 1.76% (0.55-3.13) | 0.42 (0.32-0.52) |
| Timor-Leste | 9 (4.1-17.1) | 1.9 (0.9-3) | 19.4 (12.6-27) | 1.9 (1.2-2.6) | 1.14% (0.2-2.64) | -0.14 (-0.26 to -0.02) |
| Togo | 7 (3.7-12.3) | 0.3 (0.2-0.4) | 19.5 (10-27.5) | 0.4 (0.2-0.5) | 1.77% (0.45-3.67) | 0.85 (0.78-0.92) |
| Tokelau | 0 (0-0) | 2 (0.9-2.8) | 0 (0-0.1) | 3.4 (1.7-5.1) | 0.61% (0.16-1.28) | -0.02 (-0.36-0.33) |
| Tonga | 1.2 (0.7-1.6) | 1.7 (0.9-2.2) | 1.7 (1-2.3) | 1.8 (1-2.5) | 0.39% (0-0.89) | 0.35 (0.28-0.42) |
| Trinidad and Tobago | 16.3 (14.7-17.9) | 1.6 (1.5-1.8) | 32.1 (24.4-40.8) | 1.9 (1.4-2.4) | 0.97% (0.43-1.59) | 0.47 (0.37-0.56) |
| Tunisia | 86.5 (62-140.9) | 1.4 (1-2.4) | 157.5 (101.7-259.8) | 1.2 (0.8-2) | 0.82% (0.26-1.55) | -0.55 (-0.61 to -0.5) |
| Turkey | 1743.6 (1119.6-2396.8) | 4 (2.7-5.2) | 2446.3 (1718.9-3192.7) | 2.7 (1.9-3.5) | 0.4% (0-0.96) | -1.25 (-1.39 to -1.11) |
| Turkmenistan | 40.7 (33.5-48.1) | 1.2 (1-1.4) | 58.3 (44.2-77) | 1.2 (0.9-1.5) | 0.43% (0.07-0.91) | -0.44 (-0.64 to -0.23) |
| Tuvalu | 0.2 (0.1-0.2) | 2 (0.9-2.9) | 0.2 (0.1-0.3) | 1.9 (1-2.6) | 0.34% (-0.01-0.85) | -0.09 (-0.11 to -0.08) |
| Uganda | 48.9 (25-80.4) | 0.4 (0.2-0.6) | 131.9 (70.7-205.2) | 0.4 (0.2-0.7) | 1.7% (0.39-3.37) | 0.5 (0.4-0.61) |
| Ukraine | 1130.9 (965.8-1293.3) | 1.9 (1.6-2.2) | 713.6 (527.7-930.4) | 1.1 (0.9-1.5) | -0.37% (-0.56 to -0.15) | -1.57 (-1.81 to -1.33) |
| United Arab Emirates | 26 (15.8-36.1) | 3.3 (1.8-4.8) | 118.9 (73.6-165.7) | 2.4 (1.5-3.2) | 3.57% (2.28-5.43) | -0.04 (-0.26-0.17) |
| United Kingdom | 1985.5 (1914.7-2049.1) | 2.3 (2.2-2.4) | 3430.7 (3130.3-3583.4) | 2.6 (2.4-2.7) | 0.73% (0.63-0.8) | 0.43 (0.36-0.51) |
| United States | 8954.1 (8426.5-9232.6) | 2.9 (2.7-2.9) | 16648.5 (15097-17423.6) | 2.9 (2.7-3) | 0.86% (0.78-0.91) | 0.12 (-0.01-0.24) |
| Uruguay | 70.6 (64.5-77.1) | 2 (1.8-2.2) | 106.7 (94.6-118.9) | 2.2 (2-2.5) | 0.51% (0.3-0.73) | 0.32 (0.24-0.4) |
| Uzbekistan | 246.2 (191.4-302.9) | 1.3 (1.1-1.6) | 354.4 (290.2-439.6) | 1.1 (0.9-1.4) | 0.44% (0.06-1.11) | -0.8 (-0.99 to -0.6) |
| Vanuatu | 1.7 (0.8-2.5) | 1.7 (0.7-2.5) | 4.1 (2.2-5.7) | 1.8 (0.9-2.5) | 1.4% (0.85-2.24) | 0.08 (0.04-0.13) |
| Yemen | 166.3 (78.3-309.2) | 2.3 (1.1-3.7) | 442.1 (233.5-656.8) | 2.3 (1.2-3.5) | 1.66% (0.62-3.61) | 0.07 (0.01-0.12) |
| Zambia | 37 (16.7-65) | 0.6 (0.3-0.9) | 92.1 (49-139.8) | 0.7 (0.4-1) | 1.49% (-0.03-3.65) | 0.63 (0.56-0.69) |
| Zimbabwe | 66.1 (41.6-102.1) | 1.2 (0.8-2) | 147.2 (90.8-234.7) | 1.6 (1-2.6) | 1.23% (0.52-2.21) | 0.87 (0.64-1.1) |
