# Supplementary material for: Global, regional, and national burden of acute myeloid leukemia, 1990–2021: a systematic analysis for the global burden of disease study 2021
Source: Biomark Res. 2024 Sep 11;12:101. doi: 10.1186/s40364-024-00649-y (PMC11389310; doi:10.1186/s40364-024-00649-y)
Supplement: Supplementary file 3 — Supplementary Material 3 [file 40364_2024_649_MOESM3_ESM.docx]

Table S3 The DALYs and age-standardized DALYs Rate of AML in 1990 and 2021, and its temporal trends from 1990 to 2021 in 204 countries or territories

| location | DALYs_1990 | | Age-standardized DALY rate_1990 | | DALYs_2021 | | | Age-standardized DALY rate_2021 | | Cases_change | | EAPC_CI |
| --- | --- | --- | --- | --- | --- | --- | --- | --- | --- | --- | --- | --- |
| Afghanistan | | 11300.3 (4673.9-25884.5) | | 130.9 (53.5-279.4) | | 29843.3 (15117-56310) | 137.9 (68.4-250.7) | | 1.64% (0.89-3) | | 0.27 (0.2-0.34) | |
| Albania | | 1715.9 (1175.1-2625.6) | | 57.5 (40.5-86.6) | | 1417.7 (895.7-2069.4) | 42.9 (28-60.8) | | -0.17% (-0.52-0.28) | | -0.78 (-0.98 to -0.57) | |
| Algeria | | 12364.8 (7635.9-17485) | | 46 (29-63.9) | | 13670.3 (9065.4-20751) | 32.6 (21.6-49.4) | | 0.11% (-0.27-0.77) | | -0.98 (-1.03 to -0.92) | |
| American Samoa | | 41.8 (26.5-53.9) | | 93.8 (55.1-120.9) | | 28.7 (20.7-42.8) | 57.2 (40.9-85.7) | | -0.31% (-0.54-0.25) | | -2.22 (-2.49 to -1.94) | |
| Andorra | | 56.1 (35.5-88) | | 100.4 (63.6-157.2) | | 79.1 (47-114.6) | 63.5 (37.7-91.4) | | 0.41% (-0.18-1.39) | | -1.12 (-1.26 to -0.97) | |
| Angola | | 2892.9 (1184.5-7193.4) | | 30 (13.8-57.8) | | 7268.9 (3869.1-10898.4) | 26.6 (14.5-39.9) | | 1.51% (0.04-4.04) | | -0.26 (-0.34 to -0.17) | |
| Antigua and Barbuda | | 32.9 (29.9-37.2) | | 56.5 (51.3-64) | | 62.7 (58.2-67) | 65.7 (61.1-70.2) | | 0.91% (0.65-1.18) | | 0.8 (0.53-1.07) | |
| Argentina | | 25579.4 (23627-28132.4) | | 77.1 (71.1-84.7) | | 31480.7 (29104.5-34481) | 64.2 (59.5-70.4) | | 0.23% (0.09-0.39) | | -0.31 (-0.55 to -0.07) | |
| Armenia | | 1428.8 (1092.6-1806.6) | | 42.1 (32.5-52.9) | | 1683.7 (1402.2-1987.1) | 48.4 (40.9-56.6) | | 0.18% (-0.14-0.65) | | 1.32 (0.55-2.09) | |
| Australia | | 12291 (11582.8-13070.8) | | 67.4 (63.6-71.6) | | 28143.6 (25620.5-30897.6) | 72.7 (66.8-79.7) | | 1.29% (1.06-1.54) | | 0.09 (-0.22-0.41) | |
| Austria | | 5871.4 (5516.9-6193.9) | | 63.7 (60.4-67.1) | | 8336.5 (7622.3-9076.9) | 59.1 (54.6-63.5) | | 0.42% (0.29-0.56) | | 0.18 (-0.05-0.42) | |
| Azerbaijan | | 6003.9 (4073.1-9120.9) | | 79.6 (53.9-122.1) | | 6542.3 (4290.3-10142.9) | 64.1 (41.7-98.1) | | 0.09% (-0.26-0.58) | | -0.89 (-1.03 to -0.74) | |
| Bahrain | | 321.7 (212.6-432.1) | | 90 (58.7-119.8) | | 718.3 (474.8-1146.5) | 56.6 (37.4-83) | | 1.23% (0.54-2.24) | | -1.92 (-2.09 to -1.76) | |
| Bangladesh | | 53770.6 (26868.8-120144.5) | | 45.5 (24.3-87.9) | | 57355.4 (36684.8-84317.6) | 35.9 (23-52.2) | | 0.07% (-0.61-1.17) | | -0.72 (-0.79 to -0.66) | |
| Barbados | | 210.4 (192.1-228.8) | | 83.2 (75.4-90.5) | | 305.5 (233.3-382.1) | 89.5 (68.6-112.8) | | 0.45% (0.1-0.83) | | 0.84 (0.64-1.05) | |
| Belarus | | 5447 (4539-6774.5) | | 51.2 (42.5-62.4) | | 7707.6 (6233.9-9369.7) | 66.8 (54-80.5) | | 0.42% (0.08-0.86) | | 0.53 (-0.06-1.12) | |
| Belgium | | 8811.1 (8130-9502.9) | | 71.2 (66.3-76.6) | | 13383.1 (12036.7-14785.4) | 74.1 (67.7-80.8) | | 0.52% (0.36-0.73) | | 0.16 (-0.08-0.41) | |
| Belize | | 33.6 (29.1-38.7) | | 15.8 (13.9-18.3) | | 82.5 (71.8-91.6) | 20 (17.4-22.3) | | 1.45% (1-2) | | 1.05 (0.72-1.38) | |
| Benin | | 775.1 (355-1815) | | 13.1 (6.8-25.3) | | 2034.5 (889.8-3019.6) | 14.4 (7-19.6) | | 1.62% (0.18-3.87) | | 0.53 (0.42-0.63) | |
| Bermuda | | 62.3 (55.3-71.5) | | 105.5 (93.2-121.2) | | 53.6 (44-64.9) | 68.5 (56.4-83.4) | | -0.14% (-0.31-0.06) | | -1.13 (-1.29 to -0.96) | |
| Bhutan | | 255.7 (105.9-516.2) | | 40.8 (18.9-73.7) | | 266.7 (158.5-463.3) | 37.8 (22.8-64.6) | | 0.04% (-0.58-1.12) | | -0.36 (-0.5 to -0.21) | |
| Bosnia and Herzegovina | | 2222.6 (1646.1-3092.7) | | 49.9 (37.3-70.6) | | 2255.7 (1439.1-3076.4) | 45.9 (29.7-62.2) | | 0.01% (-0.29-0.49) | | -0.04 (-0.18-0.1) | |
| Botswana | | 402.5 (232.8-643.7) | | 41.8 (25.3-66.6) | | 840.9 (508.1-1280) | 40.5 (24.8-60) | | 1.09% (0.44-2.19) | | -0.08 (-0.21-0.06) | |
| Brazil | | 103101.2 (98740.9-108127.4) | | 74.2 (71.4-77.4) | | 148940.1 (142073.6-155423.9) | 63.3 (60.3-66.2) | | 0.44% (0.36-0.54) | | -0.35 (-0.49 to -0.2) | |
| Bulgaria | | 5506.9 (5020.9-6045.2) | | 56.7 (51.3-63.2) | | 8177.2 (6517.2-9974.9) | 83.9 (66.8-102.4) | | 0.48% (0.15-0.89) | | 1.69 (1.34-2.04) | |
| Burkina Faso | | 1414.2 (623.6-3246.1) | | 12.6 (6.2-23.4) | | 3760.4 (1572.3-6191.9) | 15.2 (7-22.3) | | 1.66% (0.29-3.69) | | 1.02 (0.88-1.16) | |
| Burundi | | 1563.1 (555.9-3019.9) | | 25.3 (10.1-44.3) | | 2498.5 (980.7-4086.9) | 20.3 (8.5-32.5) | | 0.6% (-0.33-1.95) | | -0.55 (-0.67 to -0.42) | |
| Cambodia | | 9220 (4395.7-20534.6) | | 99.9 (50.5-188.7) | | 14843.8 (8840.6-21031.6) | 93.4 (56.2-131.1) | | 0.61% (-0.2-1.83) | | -0.29 (-0.34 to -0.25) | |
| Cameroon | | 1619.6 (790.8-3448) | | 14.1 (7.8-26.3) | | 4688.6 (2241.9-7137.4) | 15.6 (8-22.5) | | 1.89% (0.5-4.09) | | 0.64 (0.52-0.76) | |
| Canada | | 20118.3 (19056.6-21154.1) | | 66.7 (63.1-70) | | 36356.3 (33497.9-38965.6) | 61.1 (56.7-65.5) | | 0.81% (0.67-0.96) | | 0.12 (-0.02-0.27) | |
| Central African Republic | | 770.7 (334.6-1884.3) | | 31.4 (15.2-61.6) | | 1311.2 (654.2-2354.5) | 28 (14.7-45.1) | | 0.7% (0.11-1.6) | | -0.36 (-0.41 to -0.31) | |
| Chad | | 710.6 (323.6-1802.5) | | 10 (5-20.5) | | 2534.6 (1052.6-4759.2) | 13.8 (6.2-21.1) | | 2.57% (1.08-5) | | 1.37 (1.25-1.49) | |
| Chile | | 7410.1 (6953.1-7868.7) | | 58.3 (54.8-61.7) | | 10686.1 (9870.6-11594.3) | 49.2 (45.5-53.4) | | 0.44% (0.3-0.6) | | -0.31 (-0.47 to -0.15) | |
| China | | 851931.3 (419990.6-1454750.3) | | 75.1 (37.6-127.4) | | 548555.4 (373859.1-778262) | 37 (25.3-52.5) | | -0.36% (-0.65-0.18) | | -2.81 (-3.07 to -2.55) | |
| Colombia | | 17874.8 (16593.2-19339.3) | | 56.4 (52.5-60.3) | | 29889.9 (24745.7-35214.2) | 59 (48.7-69.8) | | 0.67% (0.38-1) | | 0.07 (-0.13-0.27) | |
| Comoros | | 120.5 (47-182.6) | | 24.3 (10.3-36.2) | | 172.2 (95.5-255.8) | 24 (13.5-35.3) | | 0.43% (-0.22-1.47) | | -0.31 (-0.58 to -0.04) | |
| Congo | | 682.7 (378.6-1302.4) | | 33.1 (20-54.7) | | 1317.1 (783-1867) | 28.3 (16.9-39.9) | | 0.93% (0.03-2.28) | | -0.6 (-0.71 to -0.49) | |
| Cook Islands | | 6.4 (3.6-9) | | 35.2 (19.7-49.4) | | 5.1 (2.8-6.7) | 27 (15.1-35.7) | | -0.19% (-0.43-0.18) | | -1.22 (-1.37 to -1.07) | |
| Costa Rica | | 1773.1 (1635.9-1966.4) | | 64.4 (59.8-71.2) | | 4227.9 (3742.1-4653.4) | 83.8 (74.4-92.2) | | 1.38% (1.06-1.71) | | 0.8 (0.55-1.05) | |
| Croatia | | 2763.8 (2334.6-3214.4) | | 49 (41.5-56.7) | | 4247.7 (3489-5131.8) | 63 (50.9-76.4) | | 0.54% (0.2-0.97) | | 1.06 (0.86-1.26) | |
| Cuba | | 7218.7 (6706.9-7839) | | 66.6 (61.9-72.3) | | 7893.4 (6808.2-9142.2) | 57 (49.1-66) | | 0.09% (-0.08-0.29) | | -0.41 (-0.64 to -0.19) | |
| Cyprus | | 685.5 (519.2-1003.3) | | 86.5 (64.7-128.7) | | 1181.9 (737.7-1519.7) | 63.1 (39.6-80.3) | | 0.72% (0.04-1.37) | | -0.64 (-0.77 to -0.5) | |
| Democratic Republic of the Congo | | 9010.1 (4196.6-18654) | | 25.8 (12.6-42.8) | | 16474.4 (8996.3-23154.6) | 22.7 (12.9-32.6) | | 0.83% (-0.04-2.16) | | -0.32 (-0.46 to -0.18) | |
| Denmark | | 6652.8 (6285.6-7077.4) | | 105.9 (100.5-112.2) | | 6329.4 (5667.2-7054.1) | 66.2 (60.3-72.8) | | -0.05% (-0.15-0.07) | | -1.37 (-1.59 to -1.14) | |
| Djibouti | | 98 (43.8-155.2) | | 22.9 (10.3-35.9) | | 252.4 (125.5-427.7) | 21.9 (10.9-37) | | 1.58% (0.56-3.08) | | -0.07 (-0.27-0.13) | |
| Dominica | | 38.7 (30-53.7) | | 58.5 (45.5-78.4) | | 46.3 (33-64.8) | 66.7 (46.3-94.1) | | 0.2% (-0.12-0.64) | | 0.55 (0.45-0.64) | |
| Dominican Republic | | 4629.1 (3053.5-6694.5) | | 60.1 (41.7-87.9) | | 5653.5 (3851.6-7927.3) | 52.2 (35.5-73.5) | | 0.22% (-0.13-0.73) | | 0.06 (-0.1-0.22) | |
| Ecuador | | 5056.8 (4590.3-5683.3) | | 52.9 (48.8-58.4) | | 11466 (9332.2-13966.5) | 65 (52.9-79.4) | | 1.27% (0.79-1.82) | | 1.33 (0.83-1.84) | |
| Egypt | | 36458.5 (22338.1-75766.2) | | 74 (46.3-143.6) | | 86317.5 (58714.2-123619.9) | 96.3 (65.8-141.9) | | 1.37% (0.25-2.85) | | 1.49 (1.21-1.77) | |
| El Salvador | | 3337.8 (2137.9-5225.2) | | 60.2 (39.4-94.7) | | 3963.8 (2429.8-5287) | 62 (38-82.4) | | 0.19% (-0.2-0.68) | | 0.16 (0.05-0.27) | |
| Equatorial Guinea | | 112.9 (51.8-244.9) | | 29.3 (14.1-50.9) | | 329 (162.8-540.2) | 26.8 (13.6-43.3) | | 1.91% (0.21-4.8) | | -0.32 (-0.41 to -0.23) | |
| Eritrea | | 750.8 (321.8-1481) | | 21.8 (9.5-36.9) | | 1467.3 (770.8-2131.3) | 23.7 (12.6-33.8) | | 0.95% (-0.09-2.67) | | 0.29 (0.17-0.4) | |
| Estonia | | 1118.7 (1002.9-1248.2) | | 73.1 (65.7-81.2) | | 1132.7 (962.2-1303.6) | 67.1 (57.9-76.3) | | 0.01% (-0.15-0.19) | | -0.38 (-0.76 to -0.01) | |
| Ethiopia | | 27446.7 (10564.6-61150.5) | | 54.1 (22.3-105.5) | | 40362.2 (21312.6-62698.8) | 40.7 (21.6-65) | | 0.47% (-0.46-2.05) | | -1.14 (-1.31 to -0.97) | |
| Fiji | | 1205.8 (744-1579.8) | | 173.7 (105.8-227.4) | | 1494.1 (820.3-2055.3) | 167.1 (92.2-229.5) | | 0.24% (-0.1-0.74) | | -0.03 (-0.22-0.16) | |
| Finland | | 3313.3 (3085.9-3558.6) | | 55.7 (52.2-59.9) | | 4048.6 (3626-4513.7) | 45.8 (41.3-50.4) | | 0.22% (0.07-0.38) | | -0.58 (-0.7 to -0.47) | |
| France | | 45775.1 (43340.1-48389.7) | | 67.9 (64.4-71.3) | | 69715.5 (61483.7-76542.7) | 64.3 (57.9-70) | | 0.52% (0.36-0.7) | | 0.16 (0-0.32) | |
| Gabon | | 274 (153.9-390.4) | | 31.5 (17.8-44) | | 457.7 (264.6-695.5) | 28.9 (16.8-43.1) | | 0.67% (0-1.57) | | -0.3 (-0.4 to -0.19) | |
| Georgia | | 3712.6 (3002.5-4715.3) | | 69.2 (55.7-87.4) | | 3105.7 (2720.5-3551.8) | 74.5 (64.8-84.9) | | -0.16% (-0.37-0.06) | | 1.1 (0.38-1.82) | |
| Germany | | 67214.5 (62046.2-72454.7) | | 68.9 (63.9-73.7) | | 100191 (91603.9-108577.5) | 65.9 (61.4-70.7) | | 0.49% (0.34-0.65) | | 0.1 (0.02-0.17) | |
| Ghana | | 4222.2 (1785.1-6905.6) | | 27.2 (12.9-38.7) | | 4956.7 (3047.9-8677.4) | 15.8 (10-27.7) | | 0.17% (-0.44-1.87) | | -2.35 (-2.8 to -1.89) | |
| Greece | | 8381 (7949.7-8826.4) | | 65.7 (62.4-68.8) | | 14941.4 (13873.6-15906.8) | 85.4 (80.6-90.1) | | 0.78% (0.66-0.91) | | 0.89 (0.78-1.01) | |
| Greenland | | 27.4 (17.3-34.4) | | 59.2 (38.1-72.5) | | 23.1 (14-32.4) | 35.5 (21.4-49.6) | | -0.16% (-0.38-0.28) | | -1.52 (-1.61 to -1.43) | |
| Grenada | | 38.9 (31.4-48.1) | | 43.3 (35.2-53.2) | | 51.3 (44.5-58.4) | 49.3 (43-55.7) | | 0.32% (0.01-0.74) | | 0.74 (0.59-0.9) | |
| Guam | | 81.1 (58.6-110) | | 64.4 (47.9-86.8) | | 118.8 (76.6-148.1) | 71.4 (45.8-89.1) | | 0.47% (0.05-0.96) | | 0.44 (-0.04-0.92) | |
| Guatemala | | 4433.1 (3479.5-5279.5) | | 44.1 (36.2-51) | | 6906.4 (5837.6-8025.5) | 45.9 (38.8-53.2) | | 0.56% (0.21-1.12) | | 0.14 (-0.07-0.36) | |
| Guinea | | 442 (161.2-1071.4) | | 5.6 (2.4-12) | | 647.1 (287.5-1127.7) | 4.6 (2.5-7.5) | | 0.46% (-0.3-1.78) | | -0.48 (-0.53 to -0.42) | |
| Guinea-Bissau | | 169.3 (78-424) | | 15.5 (8.1-33.1) | | 294.9 (146.9-417.4) | 16.4 (9-23.1) | | 0.74% (-0.19-2.16) | | 0.46 (0.31-0.61) | |
| Guyana | | 88.2 (71.9-107.8) | | 11.7 (9.7-13.9) | | 121.5 (89.1-156.9) | 16 (11.9-20.7) | | 0.38% (-0.06-0.99) | | 1.71 (1.43-1.98) | |
| Haiti | | 5120.8 (2361.3-12713.3) | | 76 (37.9-161.2) | | 8341.9 (4502.6-14943.5) | 67.2 (36.7-113.4) | | 0.63% (-0.03-1.74) | | -0.17 (-0.26 to -0.08) | |
| Honduras | | 3142.5 (1655.7-5256.6) | | 66.2 (37.4-103.1) | | 4855.3 (2903.5-7519.8) | 54.8 (34.3-82.8) | | 0.55% (-0.08-1.49) | | -0.7 (-0.76 to -0.63) | |
| Hungary | | 8796 (8238-9383) | | 71 (66.5-75.8) | | 9995.4 (8345.6-12072) | 68.4 (57.5-82.4) | | 0.14% (-0.07-0.39) | | 0.08 (-0.06-0.23) | |
| Iceland | | 167.3 (152.7-181.4) | | 62.7 (57.4-67.9) | | 290.1 (259.1-324.5) | 60.8 (54.3-67.9) | | 0.73% (0.5-1.02) | | -0.02 (-0.08-0.04) | |
| India | | 283628.2 (171035.9-502047.1) | | 34.4 (21.2-56.4) | | 439740 (320616.8-587232.2) | 32.7 (23.8-43.7) | | 0.55% (-0.17-1.33) | | -0.25 (-0.35 to -0.15) | |
| Indonesia | | 139949.7 (87371.2-233959.6) | | 82.3 (53-130.2) | | 222090.6 (156780.8-304319) | 81.2 (58.3-110.9) | | 0.59% (0.07-1.24) | | -0.06 (-0.19-0.06) | |
| Iraq | | 12034.3 (6211.1-29149.1) | | 71.7 (37.8-164) | | 23394.9 (15011-41997.5) | 64.4 (41.3-116.4) | | 0.94% (0.15-2.51) | | -0.34 (-0.39 to -0.28) | |
| Ireland | | 2226.4 (2104.5-2363.4) | | 57.7 (54.6-61.2) | | 3277.7 (3012.3-3590.4) | 48.5 (44.7-52.8) | | 0.47% (0.34-0.63) | | -0.36 (-0.68 to -0.04) | |
| Israel | | 4336.3 (3979.7-4708.1) | | 88.6 (81.5-96.4) | | 8075.4 (7405.8-8768.4) | 72.7 (67.2-78.8) | | 0.86% (0.67-1.08) | | -0.34 (-0.56 to -0.11) | |
| Italy | | 44585.4 (42799.4-46140) | | 66.6 (64.4-68.6) | | 71160.8 (64775.5-75569.7) | 66.4 (62.1-70.2) | | 0.6% (0.48-0.69) | | 0.11 (-0.1-0.31) | |
| Jamaica | | 723.9 (602.2-874.3) | | 28.8 (24.4-34.5) | | 1191.8 (896.4-1568.1) | 41.9 (31.7-54.8) | | 0.65% (0.17-1.3) | | 1.43 (1.13-1.73) | |
| Japan | | 101518.2 (99363.5-103152.3) | | 71.7 (70.3-72.8) | | 106701.5 (96598.3-112648.2) | 45.7 (43.4-47.2) | | 0.05% (-0.03-0.1) | | -1.1 (-1.31 to -0.9) | |
| Jordan | | 5649.8 (4226.9-7645.7) | | 185.2 (139.1-245.1) | | 13161.2 (9128-17559.5) | 121.7 (85.6-161.2) | | 1.33% (0.68-2.25) | | -1.58 (-1.79 to -1.38) | |
| Kazakhstan | | 11951 (9572-14892.2) | | 72.6 (58.8-90.2) | | 8863.3 (7276.2-10521.9) | 46.3 (38-54.9) | | -0.26% (-0.41 to -0.04) | | -1.31 (-1.69 to -0.93) | |
| Kenya | | 3374.4 (1970.8-5398.3) | | 15.3 (8.8-22.7) | | 7817.6 (5008.5-11398.7) | 18.3 (12.1-25.5) | | 1.32% (0.21-2.49) | | 0.99 (0.83-1.15) | |
| Kiribati | | 50.8 (18.5-84.2) | | 65.3 (23.7-106.6) | | 82.1 (31.6-137.8) | 67.2 (26.1-111) | | 0.62% (0.06-1.22) | | 0.04 (-0.05-0.12) | |
| Kuwait | | 620.6 (559.2-697.4) | | 50.1 (44.8-56.2) | | 1490 (1192.1-1811) | 35.6 (28.6-43.4) | | 1.4% (0.9-1.91) | | -0.99 (-1.55 to -0.42) | |
| Kyrgyzstan | | 1979 (1611.7-2471.6) | | 41.8 (34.5-51.9) | | 3009.5 (2470.6-3652.9) | 44.7 (36.7-54.1) | | 0.52% (0.11-1.01) | | 0.9 (0.57-1.23) | |
| Latvia | | 2684.5 (2372.1-3039.7) | | 99.3 (86.5-112.4) | | 1509.5 (1263.8-1756.8) | 60.2 (51.3-69.1) | | -0.44% (-0.53 to -0.32) | | -1.41 (-1.81 to -1.02) | |
| Lebanon | | 2557.7 (1676.3-4429.1) | | 95 (62.9-164.8) | | 4291.4 (3244.1-5959.8) | 74.7 (56.2-103.2) | | 0.68% (0.05-1.56) | | -0.52 (-0.62 to -0.43) | |
| Lesotho | | 396.3 (245.2-663.9) | | 32.5 (20.2-53.5) | | 840.7 (521.4-1565.4) | 54.3 (34.1-99.3) | | 1.12% (0.34-2.29) | | 2.22 (1.93-2.52) | |
| Liberia | | 384.1 (153.1-1118.9) | | 13 (6-29.8) | | 683.5 (307.6-1026.5) | 13.8 (6.5-20) | | 0.78% (-0.3-3.01) | | 0.65 (0.32-0.99) | |
| Libya | | 3508.1 (2416.9-5685.7) | | 105.2 (71.8-161) | | 6215.1 (3708.6-10867) | 96.7 (58.1-167.9) | | 0.77% (0.03-1.77) | | -0.08 (-0.19-0.04) | |
| Lithuania | | 2160.7 (1928.9-2408.6) | | 58.2 (52.1-64.8) | | 2854.6 (2428.9-3286.5) | 76.7 (66.1-86.7) | | 0.32% (0.11-0.57) | | 1.31 (0.96-1.66) | |
| Luxembourg | | 448.2 (423.2-473.8) | | 101 (95.5-106.7) | | 734.1 (666.3-807.7) | 81.8 (74.1-90.4) | | 0.64% (0.46-0.84) | | -0.68 (-0.84 to -0.53) | |
| Madagascar | | 2778.1 (1265.1-4649.9) | | 21.2 (9.9-31.1) | | 5024.9 (2415.9-7217.4) | 18.3 (9-26.1) | | 0.81% (-0.07-1.82) | | -0.35 (-0.47 to -0.23) | |
| Malawi | | 1202.4 (329.4-2365.1) | | 9.4 (3.1-16.7) | | 1351.3 (557-2507.5) | 7.1 (3.3-12.2) | | 0.12% (-0.47-1.67) | | -1 (-1.09 to -0.91) | |
| Malaysia | | 11609.5 (6862.6-15744.9) | | 73.2 (44.2-96.3) | | 20644.4 (14628.6-26372.2) | 65.3 (46.1-84.3) | | 0.78% (0.33-1.46) | | -0.43 (-0.54 to -0.32) | |
| Maldives | | 138.9 (73.2-314.1) | | 73.1 (44.8-140.4) | | 196.1 (122.3-296.7) | 43.2 (27.8-61.4) | | 0.41% (-0.46-1.77) | | -1.79 (-1.86 to -1.72) | |
| Mali | | 1297.1 (456.3-2927.4) | | 12.1 (5-23.7) | | 2246.2 (1107.7-3794.8) | 9 (5-14) | | 0.73% (0.03-2.22) | | -0.8 (-0.88 to -0.72) | |
| Malta | | 237.9 (217.3-258.4) | | 58.9 (53.9-63.8) | | 484.1 (427.7-539.1) | 69.2 (60.9-77.5) | | 1.04% (0.76-1.34) | | 0.6 (0.4-0.8) | |
| Marshall Islands | | 32.6 (18.1-44.5) | | 78.1 (41.8-109.4) | | 46.4 (24.9-67.3) | 85.9 (45-124) | | 0.42% (0.01-0.91) | | 0.29 (0.19-0.39) | |
| Mauritania | | 253.2 (133.2-502.4) | | 11.9 (7.1-21.9) | | 502.4 (270.3-713.6) | 12.7 (7.2-17.9) | | 0.98% (0.05-2.31) | | 0.27 (0.15-0.39) | |
| Mauritius | | 293.4 (268.1-321.8) | | 28.4 (26-31) | | 489.4 (443.3-522.3) | 33.3 (30.3-35.4) | | 0.67% (0.44-0.86) | | 3.49 (0.82-6.22) | |
| Mexico | | 54838.5 (52429.1-57617.9) | | 63.6 (61.5-66.2) | | 76402.1 (68552.4-84484) | 58.9 (52.8-65.1) | | 0.39% (0.25-0.55) | | -0.38 (-0.48 to -0.28) | |
| Mongolia | | 1269.1 (718-2225.7) | | 59.6 (35.9-97.9) | | 1731.1 (1159.9-2306.9) | 53.4 (36.2-71.5) | | 0.36% (-0.25-1.42) | | -0.32 (-0.47 to -0.16) | |
| Montenegro | | 393 (301.7-544) | | 62.7 (48-87) | | 442.9 (317.4-604.3) | 54.2 (38.6-72.6) | | 0.13% (-0.15-0.52) | | -0.31 (-0.48 to -0.13) | |
| Morocco | | 4200 (2617.3-7410.6) | | 18.6 (12.3-30.6) | | 6141 (4141-9989) | 16.8 (11.3-27) | | 0.46% (-0.08-1.19) | | -0.24 (-0.3 to -0.19) | |
| Mozambique | | 6948.2 (2039.6-14482.9) | | 38.9 (14.2-71.1) | | 11085.2 (4902.2-21477.4) | 33.6 (17.5-55.7) | | 0.6% (-0.31-3.08) | | -0.16 (-0.26 to -0.07) | |
| Myanmar | | 41699.4 (19777.5-96591.2) | | 109.1 (55.2-228.4) | | 47050.7 (33118.5-69564.4) | 85.6 (60.9-126.6) | | 0.13% (-0.41-1.1) | | -0.99 (-1.11 to -0.86) | |
| Namibia | | 365.8 (211.6-543.3) | | 32.5 (20.9-48.2) | | 702.5 (450.7-1070.3) | 33.8 (22-51.8) | | 0.92% (0.31-2.03) | | 0.07 (-0.08-0.22) | |
| Nepal | | 8019.8 (3823.3-17807.8) | | 38.9 (19-75.3) | | 10466.7 (7122.5-16316.2) | 35.4 (24.2-54.7) | | 0.31% (-0.38-1.49) | | -0.15 (-0.29-0) | |
| Netherlands | | 12373.7 (11673.1-13044.5) | | 72.6 (68.8-76.4) | | 17458.1 (15857.4-19099.2) | 62.1 (57.4-67.1) | | 0.41% (0.29-0.55) | | -0.45 (-0.62 to -0.27) | |
| New Zealand | | 2982.6 (2747-3183.7) | | 80.7 (74.7-86.1) | | 4082.4 (3678.3-4444.7) | 59.7 (54.3-64.8) | | 0.37% (0.22-0.53) | | -0.93 (-1.25 to -0.62) | |
| Nicaragua | | 1908 (1108-2943.5) | | 44.4 (28.2-67.9) | | 2260.7 (1408.1-3074.5) | 35.6 (22.6-48.1) | | 0.18% (-0.33-0.69) | | -0.44 (-0.58 to -0.3) | |
| Niger | | 1333 (481.2-3775.6) | | 12.7 (5.4-29.1) | | 2991.3 (1124.3-4783.1) | 11.9 (5.1-18.2) | | 1.24% (-0.11-3.91) | | -0.06 (-0.18-0.06) | |
| Nigeria | | 12832.7 (5665.7-30435) | | 12.3 (6.3-24.2) | | 27992.4 (13830.6-42813) | 11.6 (6.6-15.6) | | 1.18% (0.15-2.46) | | 0.03 (-0.07-0.14) | |
| Northern Mariana Islands | | 30.4 (16.8-43.9) | | 75.7 (42.4-106.4) | | 18.1 (12-35.3) | 37.2 (25.1-70.8) | | -0.4% (-0.68-0.26) | | -2.68 (-2.87 to -2.49) | |
| Norway | | 4066.4 (3910.9-4220.7) | | 77.5 (75-80.2) | | 4755.7 (4387.3-5068.1) | 57.2 (53.7-60.4) | | 0.17% (0.09-0.25) | | -0.74 (-0.87 to -0.6) | |
| Oman | | 1341.8 (807-1891) | | 77.6 (45.5-109.1) | | 1869 (1266.4-2545.2) | 49.5 (33.2-65.7) | | 0.39% (-0.07-1.12) | | -0.87 (-1.1 to -0.65) | |
| Pakistan | | 50892.5 (30921.7-91086.2) | | 44 (27.2-72.5) | | 111512.7 (74133.3-175781) | 49.2 (33.3-77.2) | | 1.19% (0.43-2.33) | | 0.37 (0.25-0.49) | |
| Palestine | | 647.8 (379.9-1144.7) | | 34.6 (20.6-58.2) | | 1176.9 (762.6-1785) | 26.8 (17.1-39.8) | | 0.82% (0.05-1.83) | | -0.91 (-1 to -0.82) | |
| Panama | | 1386.1 (1246.1-1548.7) | | 58.2 (52.8-64.7) | | 3108.3 (2530.7-3756.6) | 72.5 (59.2-87.7) | | 1.24% (0.79-1.72) | | 0.74 (0.57-0.91) | |
| Papua New Guinea | | 2989.7 (1180.1-4639.3) | | 71.9 (26.8-110.6) | | 7333.3 (3769.2-10797.8) | 70.7 (34.3-108.2) | | 1.45% (0.72-2.42) | | -0.1 (-0.16 to -0.05) | |
| Paraguay | | 1842.7 (1286.4-2552.5) | | 48.9 (34.5-68.1) | | 3747.8 (2467-5196) | 55.6 (36.6-76.5) | | 1.03% (0.46-1.93) | | 0.64 (0.45-0.84) | |
| Peru | | 13984.8 (8806.5-21431.6) | | 64.6 (42.5-97) | | 20258.2 (11178.6-27436.6) | 57.3 (31.6-77.3) | | 0.45% (-0.25-1.21) | | -0.15 (-0.26 to -0.03) | |
| Philippines | | 54925.1 (38544.5-83799.4) | | 93.9 (68.9-131) | | 90271.4 (71183.3-113857.1) | 84.1 (67-107.2) | | 0.64% (0.11-1.09) | | -0.27 (-0.33 to -0.21) | |
| Poland | | 26775.4 (24410.6-30107) | | 66.9 (61-74.2) | | 30837.1 (28189.4-33494.2) | 53.4 (49-58) | | 0.15% (0.01-0.32) | | -0.31 (-0.62 to -0.01) | |
| Portugal | | 8025.1 (7545.2-8510.8) | | 72.6 (68-77) | | 10923.2 (9764-11943.4) | 62.2 (57.1-67.1) | | 0.36% (0.22-0.51) | | -0.46 (-0.64 to -0.28) | |
| Puerto Rico | | 3244.5 (3019.2-3508.4) | | 89.7 (83.4-96.6) | | 3569.6 (2934.7-4234) | 79 (65.5-93.5) | | 0.1% (-0.11-0.33) | | -0.56 (-0.76 to -0.36) | |
| Qatar | | 224.3 (138.6-305.7) | | 74 (44.8-102.1) | | 786.4 (523-1225.8) | 38.7 (26.1-57) | | 2.51% (1.33-4.5) | | -2.12 (-2.42 to -1.83) | |
| Romania | | 12516.8 (11177.5-13766.3) | | 53.3 (47.4-58.6) | | 14611.6 (12578.7-16699.4) | 54.3 (47-61.9) | | 0.17% (-0.02-0.4) | | 0.32 (0.18-0.46) | |
| Russian Federation | | 89380.2 (81250.2-97820.2) | | 58.2 (53.1-63.8) | | 81954.2 (75433.8-88443) | 45.1 (41.8-48.2) | | -0.08% (-0.2-0.02) | | -0.86 (-1.02 to -0.7) | |
| Rwanda | | 2374.7 (946.1-4653.2) | | 30.3 (13.2-55.4) | | 2867.6 (1415.1-4507.5) | 22.7 (11.6-35) | | 0.21% (-0.52-1.46) | | -1.21 (-1.33 to -1.08) | |
| Saint Kitts and Nevis | | 4.8 (4.1-5.5) | | 11.3 (9.7-13) | | 7.3 (5.9-8.8) | 13 (10.7-15.8) | | 0.53% (0.22-0.96) | | 0.76 (0.55-0.98) | |
| Saint Lucia | | 67.1 (59.9-77.4) | | 51.7 (46.5-59.1) | | 96.6 (78.4-116.5) | 52 (42-62.9) | | 0.44% (0.08-0.81) | | 0.03 (-0.19-0.24) | |
| Saint Vincent and the Grenadines | | 62.9 (56.3-70.6) | | 53.4 (47.9-59.4) | | 62.4 (53.5-72.1) | 54.7 (46.6-63.5) | | -0.01% (-0.17-0.19) | | 0.45 (0.29-0.61) | |
| Samoa | | 158.2 (103.4-225.7) | | 100.3 (60-142.2) | | 175.4 (113.1-250.8) | 88.9 (56.4-127.1) | | 0.11% (-0.14-0.5) | | -0.41 (-0.52 to -0.3) | |
| Sao Tome and Principe | | 16.5 (8.3-25.8) | | 11.3 (6.3-16.5) | | 17.6 (9.9-30) | 8.8 (5.4-14.2) | | 0.06% (-0.45-1.2) | | -0.75 (-0.89 to -0.62) | |
| Saudi Arabia | | 4426.3 (2630.8-7468.6) | | 32.2 (20.9-55.1) | | 13844.7 (9190.3-24177) | 39 (26.7-63.5) | | 2.13% (0.94-4.43) | | 0.86 (0.51-1.21) | |
| Senegal | | 1154.7 (552.1-2407.6) | | 13.1 (7-23.6) | | 2074.6 (1112.1-3054.8) | 14.2 (7.9-20.1) | | 0.8% (-0.21-2.26) | | 0.64 (0.44-0.83) | |
| Serbia | | 7302.8 (5278.9-9816.8) | | 71.3 (50.3-99.2) | | 7446.3 (4827.2-9576.6) | 55.7 (37-71.2) | | 0.02% (-0.27-0.34) | | -0.73 (-0.84 to -0.62) | |
| Seychelles | | 49.3 (34.5-63.4) | | 70.3 (47.5-90.4) | | 55 (34.9-71.8) | 49.7 (31.9-64.9) | | 0.12% (-0.14-0.49) | | -0.56 (-0.7 to -0.42) | |
| Sierra Leone | | 645.4 (269.7-1699.3) | | 12.1 (5.7-27.8) | | 1260.6 (546.1-1963.2) | 14.1 (6.6-20.2) | | 0.95% (-0.14-2.87) | | 0.91 (0.73-1.09) | |
| Singapore | | 2198.3 (2062.2-2335.4) | | 79.2 (74.3-84) | | 3484.3 (3205.3-3756.8) | 50.8 (46.4-55.2) | | 0.58% (0.44-0.75) | | -1.14 (-1.35 to -0.92) | |
| Slovakia | | 4364.7 (3424.4-6351.8) | | 78.3 (61.2-113.5) | | 4501.5 (2714.2-5984.6) | 61.1 (38.1-81) | | 0.03% (-0.35-0.49) | | -0.55 (-0.64 to -0.46) | |
| Slovenia | | 918.8 (825.4-1027.4) | | 41.4 (37.5-46) | | 1295.7 (1066-1535.8) | 37.9 (31.2-44.4) | | 0.41% (0.16-0.75) | | 0.16 (-0.08-0.4) | |
| Solomon Islands | | 252.2 (107.9-379.7) | | 78.1 (31.8-118.6) | | 552.3 (290.1-793.7) | 84.5 (43.4-120.1) | | 1.19% (0.62-2.26) | | 0.26 (0.17-0.36) | |
| Somalia | | 1539 (523.8-3662.8) | | 20.1 (7.7-42) | | 3354.1 (1381.1-5994.9) | 18.3 (7.8-31.6) | | 1.18% (0.34-2.71) | | -0.12 (-0.24 to -0.01) | |
| South Africa | | 15311.2 (9363.4-22291) | | 48.3 (30-67) | | 24709.7 (16721.6-34697.3) | 45.8 (30.5-63.6) | | 0.61% (0.2-1) | | -0.16 (-0.3 to -0.03) | |
| South Sudan | | 1483 (563-3085.1) | | 23.6 (9.7-43.4) | | 2208.6 (923.8-4018) | 23.6 (10.6-38.8) | | 0.49% (0.01-1.23) | | 0.2 (-0.03-0.43) | |
| Spain | | 25344.8 (24040.5-26783.3) | | 58.2 (55.2-61.3) | | 38523.5 (34535.3-42033.6) | 53 (48.1-57.5) | | 0.52% (0.37-0.68) | | -0.19 (-0.3 to -0.08) | |
| Sri Lanka | | 11003.4 (6581.7-15073) | | 68.1 (41.9-93.3) | | 10670.8 (6340.3-15177.7) | 43.8 (26.6-61.9) | | -0.03% (-0.44-0.58) | | -1.55 (-1.72 to -1.39) | |
| Sudan | | 17612.5 (8133.2-40794) | | 96.7 (47.5-183.8) | | 33985.2 (19391.5-53152.2) | 93 (54.8-143.9) | | 0.93% (-0.04-2.59) | | 0.02 (-0.04-0.09) | |
| Suriname | | 199.9 (125.2-259.4) | | 51.9 (34.3-67.5) | | 305.5 (209.4-414.7) | 52.4 (35.9-70.7) | | 0.53% (0.15-1.08) | | 0.24 (0.09-0.39) | |
| Sweden | | 7100.6 (6658.1-7607) | | 62.8 (59.3-67) | | 8670.4 (7616.3-9726.6) | 50.6 (45.3-56.5) | | 0.22% (0.07-0.38) | | -0.61 (-0.75 to -0.47) | |
| Switzerland | | 6146 (5664.8-6591.4) | | 75.3 (69.6-80.6) | | 7410 (6624.5-8160.7) | 53.7 (48.7-59.2) | | 0.21% (0.07-0.33) | | -0.63 (-0.92 to -0.34) | |
| Taiwan (Province of China) | | 12237.5 (10553.6-14276.1) | | 62.7 (54.3-73.3) | | 22398.9 (20670.8-24153.1) | 74.2 (68.5-79.8) | | 0.83% (0.55-1.2) | | 0.6 (0.43-0.77) | |
| Tajikistan | | 3540.3 (2004.2-5674.7) | | 60.3 (36.8-88.2) | | 4819.3 (2641.2-8137.1) | 46.4 (26.4-76.5) | | 0.36% (-0.31-1.32) | | -1.09 (-1.28 to -0.89) | |
| Thailand | | 48985.9 (33305.4-68091.5) | | 94.3 (64.6-129.8) | | 88627.1 (42069.4-124213.4) | 110.1 (55.6-151.9) | | 0.81% (0.01-1.75) | | 0.28 (0.12-0.43) | |
| Timor-Leste | | 523.2 (230.8-1168.9) | | 73.7 (34.1-131.8) | | 887.2 (592.4-1259.4) | 69.5 (45.8-96.9) | | 0.7% (-0.14-2.11) | | -0.2 (-0.5-0.09) | |
| Togo | | 448.8 (215.8-887.6) | | 11.6 (6.4-19.3) | | 1017.6 (497.4-1425.4) | 13.6 (6.9-19.2) | | 1.27% (0.1-3.08) | | 0.75 (0.66-0.84) | |
| Tokelau | | 1.3 (0.6-1.9) | | 82.3 (38.2-118.3) | | 2.7 (1.4-4.1) | 204.9 (103.4-327.1) | | 1.01% (0.38-1.88) | | 0.52 (-0.43-1.48) | |
| Tonga | | 62.1 (36.2-83.1) | | 65.5 (36.7-86.9) | | 74.6 (43.3-104) | 72.4 (41.7-100) | | 0.2% (-0.13-0.72) | | 0.35 (0.2-0.5) | |
| Trinidad and Tobago | | 795.9 (709.7-885.5) | | 68.4 (61.4-75.5) | | 1135 (859-1454) | 73.5 (55.7-93.9) | | 0.43% (0.03-0.9) | | 0.45 (0.32-0.59) | |
| Tunisia | | 3981.4 (2841.6-6459.6) | | 52.7 (37.8-84.9) | | 5323.6 (3616.1-8524.9) | 41.8 (28.4-67.5) | | 0.34% (-0.07-0.93) | | -0.78 (-0.81 to -0.74) | |
| Turkey | | 83803.7 (47873.7-123061) | | 158.4 (96-224.7) | | 79560.3 (54765.5-101096) | 88.9 (61.3-113) | | -0.05% (-0.34-0.4) | | -2.03 (-2.2 to -1.87) | |
| Turkmenistan | | 2660.7 (2082.3-3211.9) | | 64.1 (52.8-75.5) | | 3141.8 (2409.3-4146.9) | 60.3 (46.2-79.7) | | 0.18% (-0.12-0.61) | | -0.24 (-0.56-0.09) | |
| Tuvalu | | 8.1 (4.3-12.6) | | 86.2 (45.2-130) | | 9.4 (5.4-12.9) | 76.9 (44-105.5) | | 0.16% (-0.19-0.69) | | -0.34 (-0.38 to -0.31) | |
| Uganda | | 3143.1 (1482.6-5379.9) | | 15.8 (8.1-24.9) | | 8239.3 (4252.4-13271.6) | 18.9 (10.3-29.2) | | 1.62% (0.22-3.48) | | 0.46 (0.34-0.58) | |
| Ukraine | | 50403.9 (41908.3-57897.3) | | 97.3 (78.2-113.6) | | 23798.1 (17636.3-30945.1) | 45.8 (34.7-58) | | -0.53% (-0.67 to -0.35) | | -3.01 (-3.32 to -2.71) | |
| United Arab Emirates | | 1322.6 (835.8-1887.9) | | 111.6 (64.3-156.7) | | 5129 (3224.5-7092.5) | 72.9 (47.8-96.1) | | 2.88% (1.68-4.55) | | -0.53 (-0.79 to -0.27) | |
| United Kingdom | | 50621.5 (49244.9-51806) | | 69.8 (67.9-71.1) | | 69914 (65674.9-72142.3) | 65.8 (63.2-67.7) | | 0.38% (0.32-0.43) | | -0.12 (-0.27-0.04) | |
| United States | | 246541.9 (238234.7-251576.9) | | 85.9 (83.4-87.4) | | 366031.7 (342765.7-378862) | 74 (70.3-76.3) | | 0.48% (0.43-0.52) | | -0.49 (-0.67--0.32) | |
| Uruguay | | 2664.4 (2457.7-2890.2) | | 81.6 (75.4-88.5) | | 3174.2 (2847.4-3517.4) | 80.2 (72.3-88.9) | | 0.19% (0.03-0.36) | | -0.24 (-0.4 to -0.08) | |
| Uzbekistan | | 15358.5 (11218.7-19362.1) | | 68.6 (53.6-84.2) | | 19010.6 (15255.3-23653.3) | 54.9 (44.1-68.4) | | 0.24% (-0.12-0.92) | | -0.78 (-1.09 to -0.48) | |
| Vanuatu | | 97.4 (47.9-142.2) | | 69.6 (32.2-102.1) | | 215.9 (117.6-294.2) | 72.8 (39-101.4) | | 1.22% (0.69-1.99) | | 0.08 (-0.02-0.17) | |
| Yemen | | 8576.6 (3694.7-19011.1) | | 79.3 (37.2-139.4) | | 20153.6 (10809.7-29915) | 79.7 (42-118.9) | | 1.35% (0.25-3.37) | | 0.07 (-0.04-0.17) | |
| Zambia | | 2459.5 (1020.2-4665.4) | | 26.9 (12.5-45) | | 5463.5 (2777.6-8399.4) | 29.6 (16.1-44.4) | | 1.22% (-0.2-3.5) | | 0.52 (0.38-0.66) | |
| Zimbabwe | | 3040.4 (1721.4-4503.3) | | 40.2 (25.8-63.6) | | 7024.2 (4328.8-11077.7) | 55.9 (34.4-89.3) | | 1.31% (0.57-2.36) | | 1.46 (1.02-1.9) | |
