# Supplementary material for: Global, regional, and national burden of acute myeloid leukemia, 1990–2021: a systematic analysis for the global burden of disease study 2021
Source: Biomark Res. 2024 Sep 11;12:101. doi: 10.1186/s40364-024-00649-y (PMC11389310; doi:10.1186/s40364-024-00649-y)
Supplement: Supplementary file 4 — Supplementary Material 4 [file 40364_2024_649_MOESM4_ESM.docx]

**Table S4.1** Top 10 countries or territories with most incidence, death or DALYs cases in 2021.

| Countries | | Number of cases in 2021 | |
| --- | --- | --- | --- |
| Incidence | |  | |
| USA  China | | 21533  17835.2 | |
| India | | 11040.3 | |
| Japan | | 6506.4 | |
| Indonesia | | 5554.8 | |
| Germany | | 5351.9 | |
| Brazil | | 4529.7 | |
| Italy | | 3947 | |
| France | | 3782.5 | |
| United Kingdom | | 3580.1 | |
| Deaths | |  | |
| USA  China | | 16648  15311.1 | |
| India | | 10981.1 | |
| Japan | | 5578.7 | |
| Indonesia | | 5453.2 | |
| Germany | | 4944 | |
| Brazil | | 4490.7 | |
| Italy | | 3522.5 | |
| France | | 3487.8 | |
| United Kingdom | | 3430.7 | |
| Thailand | | 2912.1 | |
| DALYs | |  | |
| China | | 548555.4 | |
| India | | 439740 | |
| USA  Indonesia | | 366031  222090.6 | |
| Brazil | | 148940.1 | |
| Pakistan | | 111512.7 | |
| Japan | | 106701.5 | |
| Germany | | 100191 | |
| Philippines | | 90271.4 | |
| Thailand | | 88627.1 | |
| Egypt | | 86317.5 | |

**Table S4.2** Top 10 countries or territories with highest age-standardized rate of incidence, deaths, or DALYs in 2021.

| Countries | | Age-standardized rate in 2021 | |
| --- | --- | --- | --- |
| Incidence | |  | |
| Australia | | 4.9 | |
| Fiji | | 4.1 | |
| Afghanistan | | 3.9 | |
| USA  Jordan | | 3.8  3.7 | |
| Luxembourg | | 3.6 | |
| Greece | | 3.5 | |
| Switzerland | | 3.5 | |
| Tokelau | | 3.4 | |
| Thailand | | 3.3 | |
| Deaths | |  | |
| Fiji | | 4.3 | |
| Afghanistan | | 3.9 | |
| Jordan | | 3.5 | |
| Tokelau | | 3.4 | |
| Greece | | 3.2 | |
| Luxembourg | | 3.2 | |
| Thailand | | 3.1 | |
| Australia | | 3 | |
| USA  Egypt | | 2.9  2.9 | |
| DALYs | |  | |
| Tokelau | | 204.9 | |
| Fiji | | 167.1 | |
| Afghanistan | | 137.9 | |
| Jordan | | 121.7 | |
| Thailand | | 110.1 | |
| Libya | | 96.7 | |
| Egypt | | 96.3 | |
| Cambodia | | 93.4 | |
| Sudan | | 93 | |
| Barbados | | 89.5 | |

**Table S4.3** Top 5 countries or territories with the most increase and decrease in age-standardized rate of incidence, deaths, or DALYs from 1990 to 2021.

| Countries | | 1990–2021 EAPCs | |
| --- | --- | --- | --- |
| Incidence | |  | |
| Mauritius | | 5.12 | |
| Jamaica | | 2.39 | |
| Bulgaria | | 2.36 | |
| Georgia | | 2.31 | |
| Lithuania | | 2.18 | |
| Northern Mariana Islands | | -3.23 | |
| American Samoa | | -2.84 | |
| Ukraine | | -2.2 | |
| Ghana | | -2.16 | |
| Qatar | | -1.63 | |
| Deaths | |  | |
| Lithuania | | 2.17 | |
| Belize | | 1.85 | |
| Mauritius | | 1.84 | |
| Guyana | | 1.84 | |
| Bulgaria | | 1.83 | |
| Northern Mariana Islands | | -2.4 | |
| American Samoa | | -2.1 | |
| Ghana | | -1.59 | |
| Ukraine | | -1.57 | |
| China | | -1.54 | |
| DALYs | |  | |
| Mauritius | | 3.49 | |
| Lesotho | | 2.22 | |
| Guyana | | 1.71 | |
| Bulgaria | | 1.69 | |
| Egypt | | 1.49 | |
| Ukraine | | -3.01 | |
| China | | -2.81 | |
| Northern Mariana Islands | | -2.68 | |
| Ghana | | -2.35 | |
| American Samoa | | -2.22 | |
