# Supplementary material for: Global, regional, and national burden of acute myeloid leukemia, 1990–2021: a systematic analysis for the global burden of disease study 2021
Source: Biomark Res. 2024 Sep 11;12:101. doi: 10.1186/s40364-024-00649-y (PMC11389310; doi:10.1186/s40364-024-00649-y)

Figure S1 The incidence cases, death cases and DALY cases of AML of three age groups in different SDI quintiles from 1990 to 2021


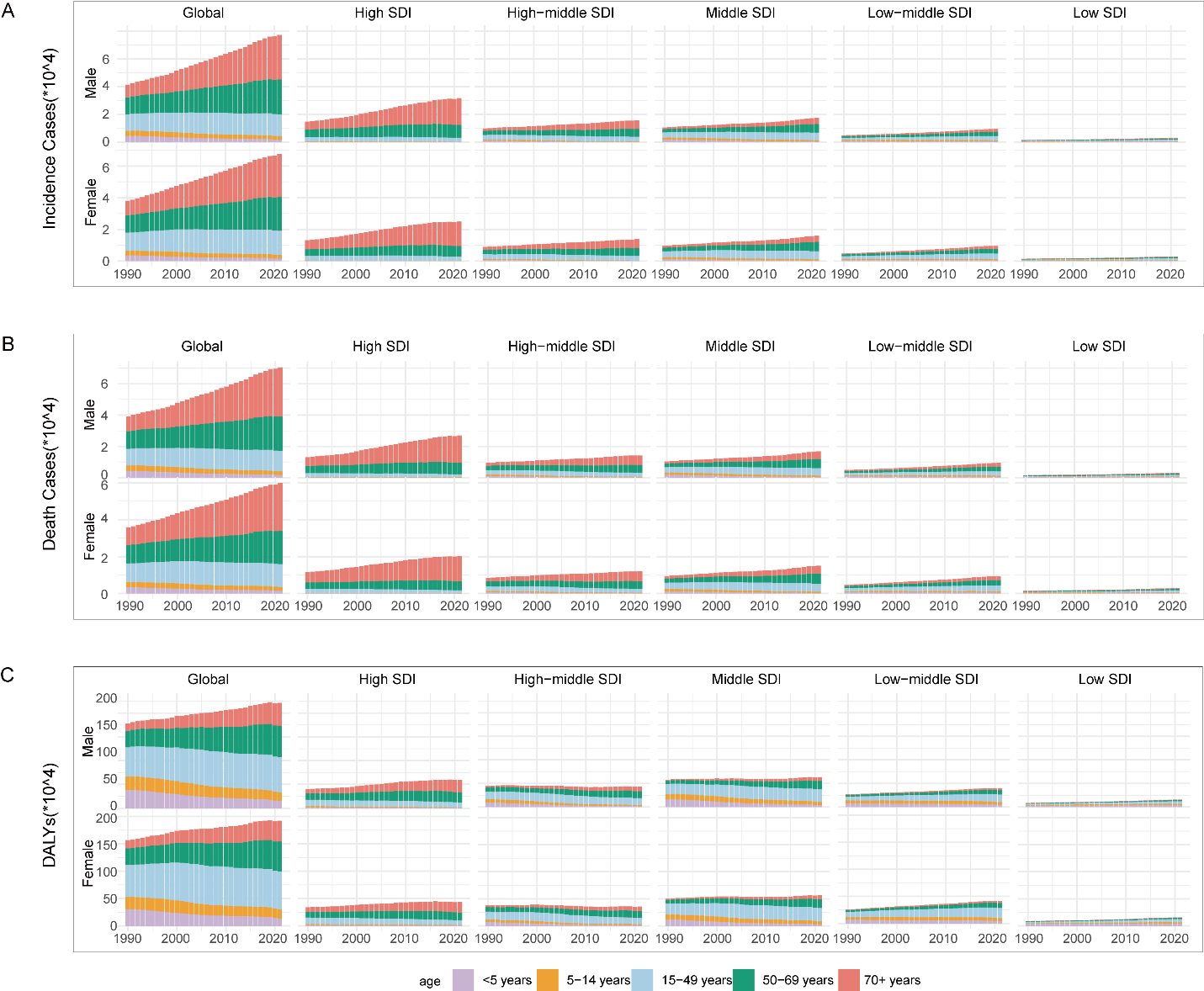

Supplement: Supplementary file 5 — Supplementary Material 5 [file 40364_2024_649_MOESM5_ESM.docx]
